# Antioxidants in Plant‐Based Food Matrices: From Structure–Activity and Degradation Kinetics to Formulation Design

**DOI:** 10.1111/1541-4337.70532

**Published:** 2026-06-18

**Authors:** Márcio Vargas‐Ramella, Carmen Silvia Favaro‐Trindade, Bárbara Miranda‐Vilela, Mariana Estefanuto‐Costa, Marina Franco‐Brito, Bibiana Alves dos Santos, Paulo Cezar Bastianello Campagnol

**Affiliations:** ^1^ Departamento de Ciências Biológicas, Centro de Educação Superior da Região Sul–CERES Universidade do Estado de Santa Catarina–UDESC Laguna Santa Catarina Brazil; ^2^ Departamento de Engenharia de Alimentos, Faculdade de Zootecnia e Engenharia de Alimentos (FZEA) Universidade de São Paulo (USP) Pirassununga São Paulo Brazil; ^3^ Departamento de Tecnologia e Ciência dos Alimentos, Centro de Ciências Rurais Universidade Federal de Santa Maria (UFSM) Santa Maria Rio Grande do Sul Brazil

## Abstract

Plant‐based antioxidants are widely incorporated into foods to retard oxidative deterioration and to deliver health‐related benefits. Yet, their in‐product and in vivo performance frequently diverges from predictions based on solution‐phase chemical assays, because matrix interactions, processing history, and host metabolism reshape both stability and bioactivity. This review integrates molecular mechanisms, structure–activity relationships (SARs), and degradation kinetics with food‐matrix and human‐relevance considerations to explain when, where, and how plant‐based antioxidants act. Evidence is collated across polyphenols, carotenoids, tocopherols and tocotrienols, and selected alkaloids, linking hydrogen‐atom, single‐electron, and proton‐coupled transfer pathways. Together, with those evidences, transition‐metal chelation, interactions with proteins and polysaccharides, interfacial partitioning in emulsions, and metal‐catalyzed oxidation are discussed. The influence of conventional and emerging processing, storage, and delivery systems (Pickering and double emulsions, spray drying/chilling, complex coacervation, ionic gelation) on antioxidant stability, localization, and bioaccessibility is also examined. Across systems, efficacy is governed less by intrinsic reactivity or nominal polarity than by effective interfacial concentration, partitioning behavior, and metal management. Encapsulation, when matched to matrix and process, improves antioxidant retention. Combinations of antioxidants may act cooperatively under one set of conditions and become antagonistic, or even pro‐oxidant, under high oxygen availability or at suboptimal molar ratios. In this review, solution‐phase rankings (DPPH, ABTS, FRAP, ORAC) are interpreted as descriptors of intrinsic reactivity. They cannot, on their own, predict performance in real food matrices or the post‐digestion metabolite pool reaching systemic circulation. Effective use of plant antioxidants in foods therefore requires that formulation choices be informed by interfacial kinetics, food‐component interactions during digestion, gut–microbiota metabotypes, and biomarker‐validated estimates of dietary intake.

## 1 Introduction

Oxidative reactions are a principal cause of sensory deterioration and nutrient loss in foods, and reactive oxygen species (ROS) are implicated in diverse pathophysiological processes in humans, reinforcing longstanding interest in dietary antioxidants and their technological roles in food systems (Gulcin 2025; Rudrapal et al. 2022; Y. Wu et al. 2024). However, predicting antioxidant performance from the food matrix to the consumer requires the integration of three traditionally disjoint domains: (i) the chemical/mechanistic domain (reactivity rules, structure–activity relationships [SARs], interfacial kinetics); (ii) the food‐matrix domain (digestion and absorption, which restructure polyphenol [PP]–biopolymer complexes and determine the systemic fraction) (Di Pede et al. 2023; Parmenter et al. 2023; Pidgeon et al. 2025); and (iii) the human‐relevance domain, including colonic microbial biotransformation, in which gut bacteria convert parent PPs into low‐molecular‐weight metabolites (phenyl‐γ‐valerolactones, hydroxyphenyl‐propionic acids, urolithins, enterolignans) that dominate systemic exposure and display distinct reactivities (Parmenter et al. 2023; Pidgeon et al. 2025).

The present review is not intended as a compilation of in vitro chemical‐assay results. Its aim is to interpret those results in the context of interfacial kinetics, food‐matrix and microbiota interactions, and biomarker‐validated dietary exposure. Solution‐phase rankings (DPPH, ABTS, FRAP, ORAC) are therefore treated as descriptors of intrinsic reactivity under homogeneous conditions; they capture neither matrix partitioning and interfacial localization in real foods nor the post‐digestion microbial‐metabolite pool that dominates systemic exposure and cannot by themselves support claims about efficacy in humans (Di Pede et al. 2023; Lomozová et al. 2021; Pidgeon et al. 2025). Recent evidence indicates that PP bioactivity in humans depends on dose, gastrointestinal interactions, and microbial conversion to absorbable phenolic catabolites, which together determine the systemic exposure profile.

Mechanistically, phytochemicals act through complementary chain‐breaking and preventive pathways. Chain‐breaking actions suppress radical propagation via hydrogen‐atom transfer (HAT), single‐electron transfer (SET), proton‐coupled electron transfer (PCET), sequential proton loss electron transfer (SPLET), or radical‐adduct formation. In contrast, preventive actions inhibit radical generation by chelating transition metals or decomposing hydroperoxides (Barouh et al. 2022; Bensid et al. 2022; Bešlo et al. 2023). SARs govern these processes: catechol/pyrogallol motifs, conjugation, and planarity (e.g., the Bors criteria for flavonols); and local steric/electronic environments modulate bond‐dissociation enthalpies and radical stabilization (Bešlo et al. 2023; Bors et al. 1990). Carotenoids excel at singlet‐oxygen quenching but may switch to pro‐oxidant behavior at high oxygen tension or excessive doses. In contrast, tocopherols/tocotrienols terminate lipid peroxidation via chromanol H‐donation, with side‐chain features and interfacial positioning shaping mobility and efficacy (Barouh et al. 2022; Muñoz and Munné‐Bosch 2019; Szewczyk et al. 2021; Zhuang et al. 2022).

In multiphase foods, where antioxidants reside is as decisive as what they can do. Oxidation often initiates at oil–water interfaces; thus, effective interfacial concentration, emulsifier identity, and barrier architecture (e.g., protein films or Pickering particles) frequently override the classical “polar paradox,” which predicts performance based solely on bulk polarity (Berton‐Carabin and Villeneuve 2023; Farooq et al. 2021; McClements and Decker 2018). Complexation of PPs with proteins or polysaccharides (PSs) can densify interfacial layers and hinder pro‐oxidant ingress; in other configurations, the same interactions sequester antioxidants away from the reactive species they should scavenge (Z. Ma, Zhou, et al. 2025; Manzoor et al. 2024; Xue et al. 2024). Transition metals (Fe, Cu) catalyze hydroperoxide breakdown and radical formation, so chelation near the interface can be decisive. In some systems, however, chelators mobilize bound metals or accelerate H_2_O_2_ decomposition, which is why chelator selection must be considered jointly with matrix composition (Gulcin and Alwasel 2022; Mertens et al. 2022; L. Zhang et al. 2023).

Processing and storage further modulate stability and function. Conventional thermal operations (pasteurization, sterilization/UHT, drying, cooking, frying) can degrade heat‐sensitive compounds or alter isomer ratios. In contrast, novel thermal (microwave, infrared, radiofrequency [RF], ohmic) and nonthermal (high‐pressure processing [HPP], pulsed electric fields [PEFs], cold plasma, ultrasound, supercritical CO_2_, ozone, ultraviolet [UV]) technologies aim to reduce thermal load and preserve bioactivity (Ağagündüz et al. 2025; Leong and Oey 2022; Y. Wu et al. 2024). Hurdle strategies that combine moderate treatments often achieve additive or supra‐additive stabilization with fewer quality trade‐offs, provided that process windows are aligned with antioxidant degradation kinetics and with the microenvironments where oxidation initiates (Bigi et al. 2023; Liao et al. 2020; C. P. Wu et al. 2022). Nevertheless, most studies evaluate processing effects in isolation, without integrating kinetic constraints, matrix interactions, and interfacial localization, which limits the transferability of results to real food systems.

Delivery systems connect chemistry to place and time. Encapsulation and interfacial engineering, including double/Pickering emulsions, spray drying/chilling, complex coacervation, ionic gelation, and biosorption, can protect actives during processing, position them at reactive interfaces, and tune release and bioaccessibility during storage and digestion (Cheng et al. 2023; Hossen et al. 2024; L. Zhang, Yu, et al. 2021). Multi‐antioxidant systems may outperform single molecules when regeneration networks (e.g., PPs restoring oxidized α‐tocopherol) and complementary functions (radical scavenging plus metal chelation) are tuned to dose, location, and oxygen availability; the same blend, however, may flip to antagonism or pro‐oxidant behavior under suboptimal ratios or high O_2_ (Bayram and Decker 2024; Parra‐Escudero et al. 2025; Zhong et al. 2025).

In this context, synergistic and antagonistic outcomes are increasingly recognized as emergent properties of spatial organization and reaction kinetics rather than intrinsic properties of individual compounds. Dietary exposure is heterogeneous even before food‐matrix and digestion effects are considered. Biomarker‐anchored cohort and intervention data show that urinary flavan‐3‐ol biomarkers track acute rather than habitual intake (Almanza‐Aguilera et al. 2021), that plasma carotenoid responses are nonlinear and adverse signals for β‐carotene segregate to supplemental rather than dietary doses (Böhm et al. 2021), and that guideline‐level intakes for flavan‐3‐ols are associated with cardiometabolic benefit (Crowe‐White et al. 2022; Lagou et al. 2025). Two consequences for food‐design research follow: Nominal‐composition or food‐frequency estimates are an unreliable proxy for systemic exposure, and the dose window relevant to dietary exposure is narrower than the in vitro literature implicitly assumes. Antioxidant performance is therefore evaluated here against realistic dietary doses and validated biomarkers rather than nominal in vitro activity.

Despite extensive literature, the three domains introduced above remain operationally fragmented. The chemical domain (DPPH/ABTS/FRAP/ORAC values and SAR rules) describes intrinsic reactivity in homogeneous solution but does not predict performance in real matrices, in which partitioning, interfacial localization, and metal accessibility govern the observed kinetics (Berton‐Carabin and Villeneuve 2023; Losada‐Barreiro et al. 2024). The food‐matrix domain (processing, storage, packaging, encapsulation) is well developed in quantitative kinetic terms (compound‐specific *E_a_*, *Q*_10_, oxygen transmission rate [OTR]‐coupled headspace dynamics) but is rarely connected to the chemical mechanisms operating in those same matrices (Suhag et al. 2025). The human‐relevance domain (daily‐intake estimates, validated plasma and urinary biomarkers, microbiota‐dependent metabotypes) has advanced largely outside the food‐science literature, so conclusions drawn from in vitro chemical assays often misrepresent the bioactive pool that actually reaches systemic circulation (Di Pede et al. 2023; Pidgeon et al. 2025).

Even within the human‐relevance domain, food‐component interactions further modulate exposure: Dairy proteins, dietary fibers, and lipid emulsifiers measurably alter the kinetic profile of flavan‐3‐ol absorption and microbial conversion, and whole‐food matrices in some cases protect flavan‐3‐ols and flavonols from upper GI degradation rather than restricting their release relative to the corresponding extracts (Cattivelli et al. 2025; Shukla et al. 2025). These matrix‐level effects, together with metabotype‐dependent ring‐cleavage capacity now resolved at the operon level for ellagitannin metabolism (Pidgeon et al. 2025), shape the metabolite pool that drives biological response, a translational gap that food‐design research has only recently begun to address. For this reason, food‐science conclusions about antioxidant efficacy in humans are reliable only when the chemical, food‐matrix, and human‐relevance domains are considered together.

Addressing these gaps requires integrating molecular mechanisms and SAR with matrix mapping, process/packaging set points derived from fitted degradation kinetics, and in situ validation under both accelerated and real‐time storage with sensory endpoints (Suhag et al. 2025; W. Zhang, Luo, et al. 2021). Although earlier reviews have examined antioxidant chemistry and applications from a broad structural perspective (Abeyrathne et al. 2022) or from the standpoint of processing effects and food applications (Suhag et al. 2025), the present review brings these threads together within a single analytical scheme. The review (i) summarizes antioxidant mechanisms and structure–activity rules across the main phytochemical classes; (ii) describes how antioxidants localize and interact within food microenvironments; (iii) analyzes how processing, storage, and packaging modulate stability and efficacy; (iv) appraises encapsulation and multi‐antioxidant strategies; and (v) proposes practical criteria for selecting antioxidant systems, processing windows, and packaging conditions under realistic dietary exposure in plant‐based foods.

## 2 Antioxidant Mechanisms

The antioxidant activity of phytochemicals is multifunctional and is commonly classified, based on their role in oxidative processes, into two main categories: primary antioxidants (Type I) and secondary antioxidants (Type II) (Dintcheva and D'anna 2019). This capacity is determined by the compound's molecular structure, which governs the transfer of reducing equivalents and the formation of stable complexes.

However, in real food systems, antioxidant mechanisms do not operate in isolation or under idealized conditions. The physicochemical environment, including phase distribution, polarity, pH, oxygen availability, and the presence of pro‐oxidants, strongly influences their effectiveness. As a result, the dominant mechanism depends on both molecular structure and the microenvironment in which the antioxidant is located.

### 2.1 Primary (Chain‐Breaking) Antioxidants (Type I): Radical Scavengers

**FIGURE 1:**
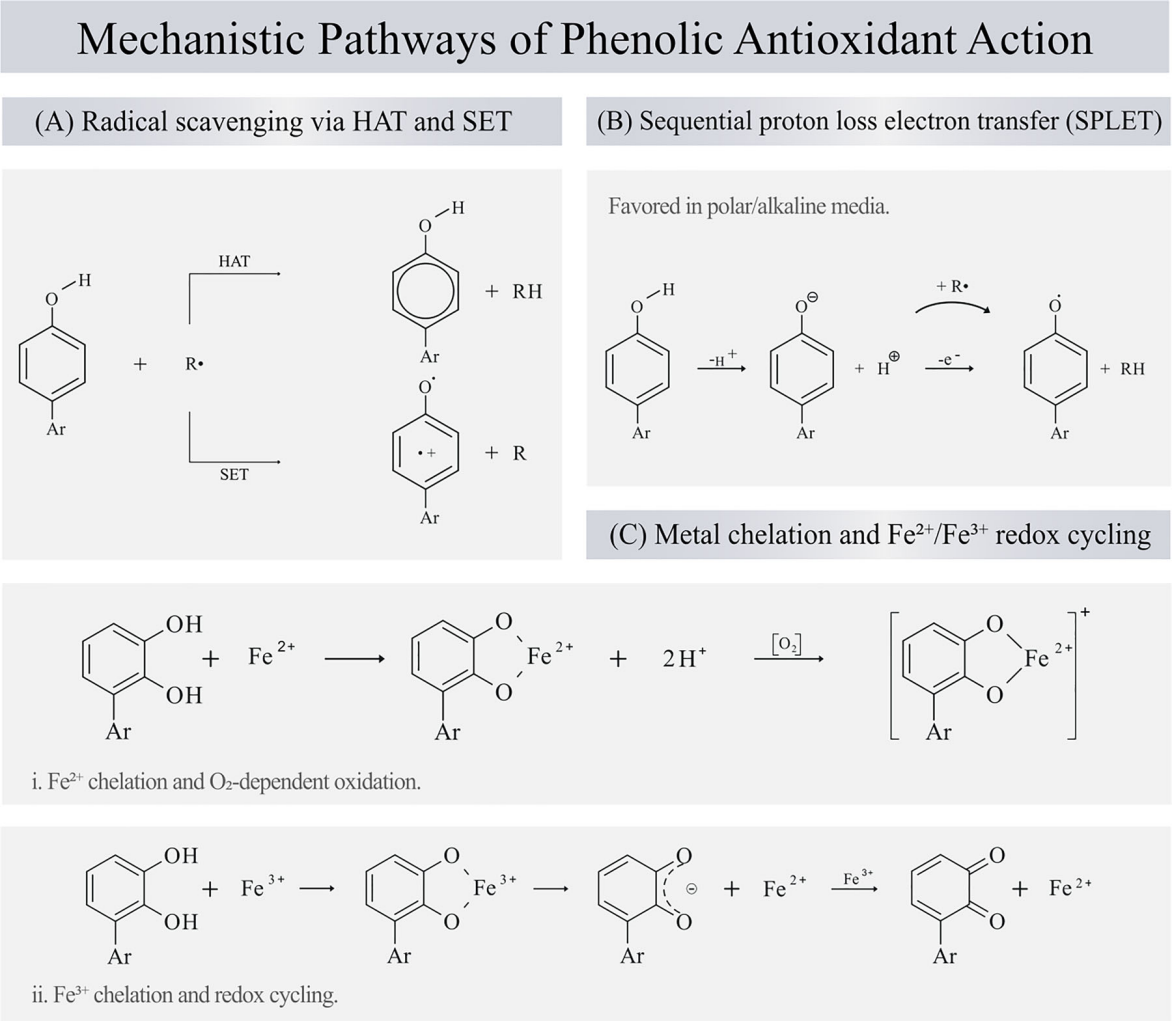
Mechanistic pathways of phenolic antioxidant action. (A) Radical‐scavenging mechanisms involving hydrogen‐atom transfer (HAT) and single‐electron transfer (SET), leading to the formation of stabilized phenoxyl radicals. (B) Sequential proton loss electron transfer (SPLET), in which deprotonation precedes electron transfer, a pathway favored under polar and alkaline conditions. (C) Metal chelation and Fe^2+^/Fe^3+^ redox cycling: (i) coordination of Fe^2+^ by phenolic ligands followed by O_2_‐dependent oxidation, and (ii) chelation of Fe^3+^ and subsequent redox cycling involving semiquinone and quinone formation. Ar denotes the aromatic moiety of phenolic compounds, and R• represents a generic radical species.

Primary antioxidants interrupt radical‐chain propagation by converting reactive species (R•) into stable products (Rudrapal et al. 2024). This action is mediated by transfer of reducing equivalents (electrons or hydrogen atoms) and can proceed via five thermodynamically governed pathways (Figure 1) (Bensid et al. 2022; Zeb 2020) as follows (i–v):

- HAT: HAT involves direct donation of a hydrogen atom from the antioxidant (ArOH) to the free radical (R•), thereby reducing it to RH and forming a less reactive ArO• phenoxyl radical (Bešlo et al. 2023):
ArOH+R•→ArO•+R−H

Efficiency is determined by the bond dissociation enthalpy (BDE) of the O–H bond and is typically higher for phenolic compounds and conjugated aromatic rings (Amic et al. 2007; Olvera‐Aguirre et al. 2023).

In food systems, HAT is often the dominant mechanism in lipid‐rich environments, such as bulk oils or fat phases of emulsions, where proton availability is limited and direct hydrogen donation becomes kinetically favorable. Under these conditions, reaction rates and diffusion toward lipid radicals become critical determinants of antioxidant performance.

ii. SET: In the first step of SET, the antioxidant (ArOH) donates an electron (e^−^) to R•, forming an antioxidant radical cation (ArOH•^+^) and a stable anion (R^−^) (Bešlo et al. 2023; Zeb 2020):
ArOH+R•→ArOH•+R−

This mechanism is favored by low ionization potentials (IP) (Rudrapal et al. 2024). SET mechanisms are more relevant in polar environments, such as aqueous phases or protein‐rich systems, where solvation stabilizes charged intermediates. However, in heterogeneous food matrices, the effectiveness of SET may be limited by phase separation and restricted interaction with lipid radicals.

iii. SPLET: SPLET involves deprotonation of the antioxidant followed by electron transfer. It is thermodynamically favored in polar and alkaline environments (Zeb 2020). Although SPLET is frequently described as a dominant mechanism for phenolics in solution, its relevance in real foods is often constrained by limited water activity, restricted proton mobility, and the typically near‐neutral pH of many food systems. In lipid‐rich or low‐moisture matrices, this pathway may be suppressed, shifting the mechanism toward HAT or interfacial PCET processes.
iv. PCET: PCET describes the concerted transfer of an electron and proton through a single transition state (Bešlo et al. 2023):
$$\mathrm{ArO}-H\;+R^{•}\to \;\mathrm{Ar}O^{•}+\;R-H$$

This mechanism is particularly relevant in heterogeneous systems, such as oil–water interfaces, where hydrogen‐bond networks and interfacial organization facilitate coupled proton–electron movement. In such environments, PCET can dominate over purely stepwise mechanisms, especially when antioxidants are localized at interfaces where both polar and non‐polar species coexist.

v. Radical adduct formation (RAF): RAF involves the addition of R• to the aromatic ring or double bonds of the antioxidant, forming a stable adduct (Dintcheva and D'anna 2019):
$$\mathrm{ArOH}\;+R^{•}\to {\left[\mathrm{ArOH}-R\right]}^{•}\to \mathrm{metabolites}\;\mathrm{or}\;\mathrm{Ar}O^{•}+\mathrm{RH}$$

Although less frequently discussed, RAF may become relevant under conditions of high radical flux or when structural features favor addition reactions. However, its contribution to food systems is generally secondary to that of HAT and PCET pathways.

Importantly, in real food matrices these mechanisms coexist and compete. Their relative contributions are governed by kinetic factors (rate of H‐atom abstraction, electron‐transfer barrier, diffusion to the oxidation locus) rather than by thermodynamic preference alone. Solution‐phase assays (DPPH, ABTS) probe only intrinsic thermodynamic feasibility and therefore give no quantitative indication of which mechanism will dominate in heterogeneous food matrices, a translational caveat that recurs throughout Sections 3 and 6.

### 2.2 Secondary Antioxidants (Type II): Preventive

Secondary antioxidants do not act directly on radicals; rather, they prevent the formation of new reactive species or regenerate oxidized primary antioxidants (Dintcheva and D'anna 2019). The major mechanisms include two pathways:

- Transition‐metal chelation (TMC): Formation of stable complexes with transition‐metal ions (Fe^2+^, Cu^2+^) inhibits catalysis of pro‐oxidant reactions (Gulcin and Alwasel 2022):
$${\left(Fe^{2+}+H_{2}O_{2}\to Fe^{3+}+OH^{-}+OH^{•}\right)}^{1}$$
$${\left(O^{•-2}+H_{2}O_{2}\to O_{2}+H_{2}O+OH^{•}\right)}^{2}$$

Where “^1^” is the Fenton reaction, and “^2^” is the Haber–Weiss reaction.

Chelation forms inert complexes (Gulcin and Alwasel 2022):

$$M^{n+}+L\;\to {\left[\mathrm{ML}\right]}_{\mathrm{inert}}$$

In food systems, metal chelation is particularly relevant in aqueous or protein‐rich phases, where transition metals are more soluble and catalytically active. The effectiveness of chelation depends on binding affinity, on the accessibility of metal ions within the matrix, and on competition with endogenous ligands, such as proteins or phosphates.

ii. Hydroperoxide decomposition: Compounds that decompose hydroperoxides convert ROOH into more stable products (ROH) (Bensid et al. 2022). This mechanism plays a critical role in preventing lipid oxidation propagation, particularly at advanced stages when hydroperoxides accumulate. Its effectiveness depends on the localization of antioxidants within lipid domains or interfaces, where hydroperoxides are generated and decomposed (Barouh et al. 2022).

Secondary mechanisms frequently act in concert with primary antioxidant pathways, forming integrated networks that combine radical scavenging, metal chelation, and regeneration. In complex food matrices, this interplay is essential for controlling oxidation, as no single mechanism is sufficient under all conditions.

## 3 Structural Classes: PPs, Carotenoids, Tocopherols and Tocotrienols, Alkaloids, and Other Phytochemicals (Tyrosol, Hydroxytyrosol, Vanillin)

### 3.1 PPs: Flavonoids, Stilbenes, Lignans, Tannins, and Phenolic Acids

**FIGURE 2:**
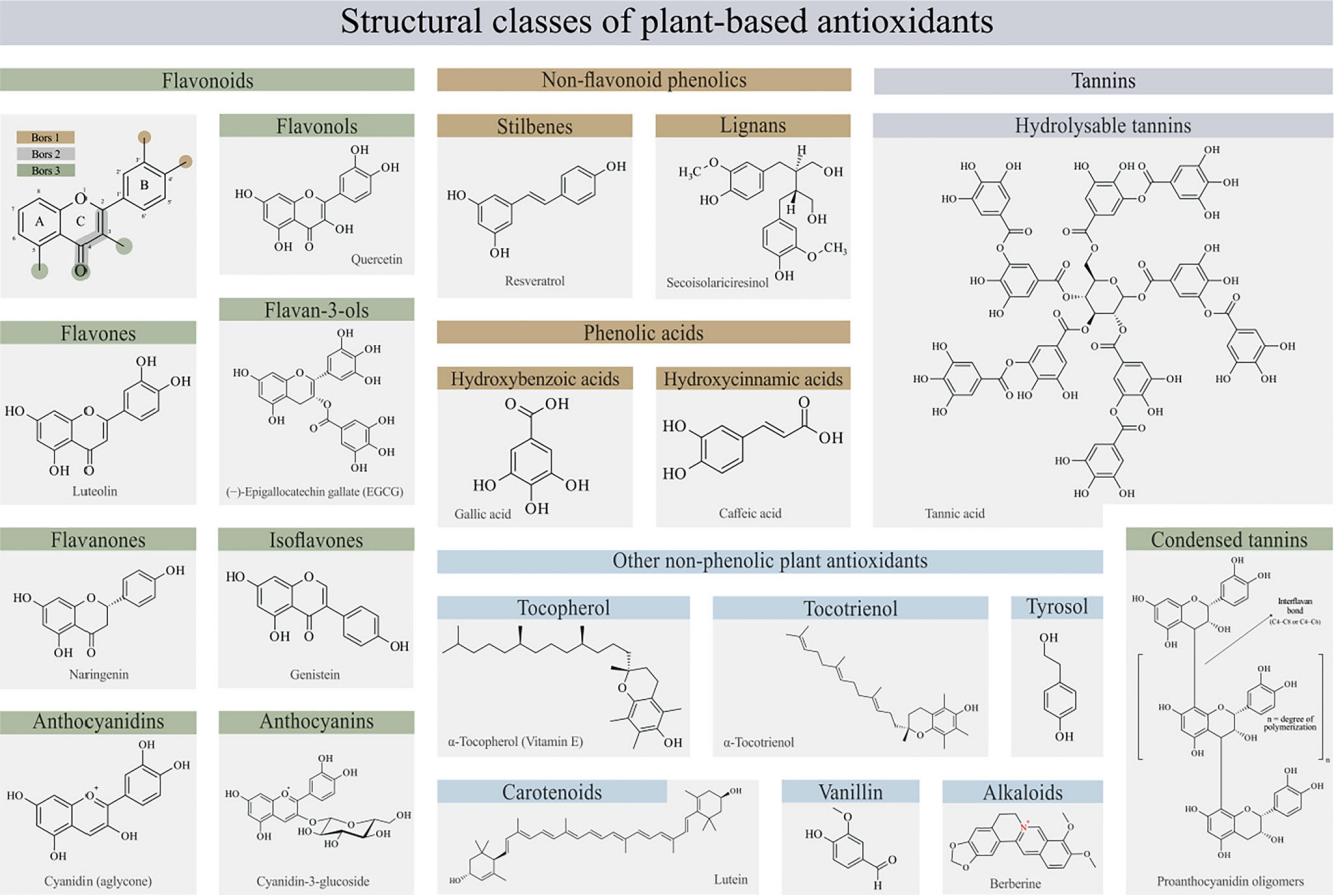
Structural classes of plant‐based antioxidants. The general C6–C3–C6 flavonoid backbone and key structural features associated with antioxidant activity (Bors criteria) are highlighted.

PPs comprise the largest and most diverse class of phytochemicals with antioxidant activity. They are defined as plant secondary metabolites derived from the shikimic‐acid and/or acetate–malonate pathways and characterized by multiple phenolic rings (Zagoskina et al. 2023). Their high antioxidant versatility arises from multiple hydroxylated aromatic rings, which enable HAT, SET, SPLET, and metal chelation in different environments (Ciupei et al. 2024; Rudrapal et al. 2024). SAR studies indicate that the position and number of hydroxyl groups, electronic conjugation, and molecular planarity determine phenoxyl radical stability and the potential to neutralize ROS (Figure 2) (Bešlo et al. 2023; Rasha and Khalid Mustafa 2025; Rosales and Fabi 2023; Zeb 2020).

However, the in‐matrix performance of PPs is governed less by intrinsic reactivity than by phase distribution, interfacial enrichment, and competitive binding to biopolymers. Effective interfacial concentrations of phenolic antioxidants in oil‐in‐water emulsions, quantified by the pseudophase kinetic model, are 20‐ to 200‐fold higher than the stoichiometric (bulk) concentration, and induction times for conjugated‐diene formation correlate linearly with the interfacial (not the bulk) value (M. Costa et al. 2021; Losada‐Barreiro et al. 2024). Conversely, PP–protein complexation can either anchor the active at the interfacial film and prolong induction times (e.g., the ovalbumin–procyanidin system, in which the Day‐6 emulsion oxidation degree decreased from 5.90% to 1.78%; Wen et al. 2022) or sequester it in dispersed aggregates that lower the effective free fraction (Tang et al. 2025). The matrix‐dependent quantitative basis of these effects is consolidated in Section 6.

A translational caveat applies to all SAR predictions for flavonoids. DPPH/ABTS/FRAP rankings consistently place quercetin above myricetin, yet in oil‐in‐water emulsions at near‐neutral pH, myricetin produces interaction indices of 3.00 and 3.63 with α‐tocopherol (at 2:1 and 1:1 ratios, respectively), whereas the closely related taxifolin is antagonistic under the same conditions, because redox‐potential‐governed regeneration of the α‐tocopheroxyl radical, not DPPH‐scavenging capacity, determines in‐matrix performance (Bayram et al. 2023; Bayram and Decker 2024). For flavan‐3‐ols the in vitro SAR is largely decoupled from in vivo outcome because parent compounds contribute marginally to systemic exposure (mean bioavailability 31% ± 23% across 49 human studies), whereas microbial phenyl‐γ‐valerolactones dominate plasma and urinary profiles, two specific PVLs accounting for more than 75% of urinary recovery in free‐living adults (Di Pede et al. 2023; Parmenter et al. 2023). SAR in this class should therefore be interpreted as a predictor of intrinsic chemistry, not of physiological efficacy.

#### 3.1.1 Flavonoids

Flavonoids constitute the most abundant and widely distributed class of PPs in nature, characterized by the C_6_–C_3_–C_6_ skeleton composed of two aromatic rings (A and B) linked by a three‐carbon unit that frequently gives rise to an oxygenated heterocycle (ring C) (Câmara et al. 2021; Kruk et al. 2022; Rudrapal et al. 2022; Zagoskina et al. 2023). Although the functional diversity of this group is vast, composed of flavonols, flavones, flavan‐3‐ols, flavanones, isoflavones, and anthocyanins, its maximum antioxidant efficiency obeys stringent structural requirements, as classically described by the Bors criteria (Bors et al. 1990). The literature supports that the superior radical‐scavenging capacity of flavonoids depends on the combination of three pharmacophoric elements (Bešlo et al. 2023; Zagoskina et al. 2023):

- *o*‐Dihydroxyl (catechol) arrangement on ring B: The presence of vicinal hydroxyl groups (3′,4′‐diOH) was described by Ncongwane et al. (2025) as the principal factor for antioxidant activity. This configuration lowers the bond dissociation enthalpy (BDE) via intramolecular hydrogen bonding and enables greater electron delocalization, thereby stabilizing the resulting semiquinone radical.
- Conjugation of ring C: The existence of a C2 = C3 double bond coupled to the 4‐oxo (carbonyl) group ensures molecular planarity. This allows the unpaired electron to be delocalized across rings A, B, and C, drastically reducing the energy of the formed radical.
- Hydroxyl groups at C3 and C5: The combination of 3‐OH and 5‐OH with the 4‐oxo group maximizes both TMC and radical capture, acting cooperatively.

Complementary to radical scavenging, flavonoid structures favor the formation of thermodynamically stable complexes with transition metals (Fe^2+^, Cu^2+^), thereby acting as preventive antioxidants (Gulcin and Alwasel 2022). In addition, literature also identifies three preferential coordination domains whose presence defines the chelating potency of each subclass (Amic et al. 2007; Gulcin and Alwasel 2022; Kruk et al. 2022; Zeb 2020):

- 3′,4′‐dihydroxyl site (ring B): The catechol arrangement is the principal binding site, common to flavonoids and phenolic acids. Fe^3+^ chelation at the catechol site is typically fast and yields inert complexes that do not subsequently oxidize the flavonoid.
- 4‐keto‐3‐hydroxyl site (ring C): Interaction between the C4 carbonyl and C3 hydroxyl forms a highly efficient coordination “pocket,” especially in classes such as flavonols.
- 4‐keto‐5‐hydroxyl site (rings A/C): The 5‐OH interacts with the C4 carbonyl, though with lower affinity than the C3–C4 site due to steric rigidity.

The coexistence of these sites, most notably in flavonols, enables cooperative chelation, suppressing Fenton‐type catalytic reactions. Flavonols constitute the class with the highest theoretical antioxidant potential. The simultaneous presence of 3‐OH, the C2 = C3 double bond, and the B‐ring catechol satisfies all Bors criteria and provides the three metal‐chelation sites described, making quercetin a structural model of multifunctional efficacy (Lang et al. 2024; Rudrapal et al. 2024). The C2 = C3 bond induces coplanarity among the rings and broadens electronic delocalization after hydrogen abstraction, reinforcing the multifunctional character of this subclass as both radical scavenger and chelating agent (Rudrapal et al. 2024). However, despite their high intrinsic reactivity, the effective antioxidant performance of flavonoids in food systems is strongly modulated by their distribution within the matrix and their interactions with macromolecules.

On the other hand, another category of flavonoids is the flavones. They differ from flavonols by lacking the C3‐OH group. This eliminates one of the principal chelation sites (4‐keto‐3‐OH) and slightly reduces electron‐donation capacity, while preserving planarity and maintaining high radical stability, especially when combined with the B‐ring catechol arrangement (Bešlo et al. 2023; Câmara et al. 2021; Rudrapal et al. 2024). In practical applications, the absence of the C3–OH group may also reduce their ability to form stable complexes with proteins and metals, thereby influencing their retention and functionality, depending on the composition and pH of the food matrix.

Another flavonoid is the flavan‐3‐ols (catechins). Flavan‐3‐ols are characterized by saturation of the C2–C3 bond (absence of a double bond) and lack of a C4 carbonyl (Kruk et al. 2022). This prevents electron delocalization through ring C (failing one Bors criterion) and eliminates carbonyl‐based chelation sites. Their activity depends almost exclusively on the high number of hydroxyls (especially the galloyl group in proanthocyanidins) for HAT mechanisms (Câmara et al. 2021; Kruk et al. 2022; Rudrapal et al. 2024; Zagoskina et al. 2023). In this subclass, the 3‐galloyl group acts as the primary structural determinant of activity, frequently surpassing the effect of the B‐ring pyrogallol arrangement and conferring greater HAT efficiency (W. Wang, Le, et al. 2023). However, their high polarity and tendency to interact with proteins may limit their mobility and availability in complex matrices, particularly in protein‐rich foods, where binding can reduce their effective antioxidant action.

Concerning the flavanones group, such as naringenin and hesperidin, they exhibit a saturated ring C and lack C3–OH. This nonplanar conformation reduces electronic conjugation, making them less potent antioxidants (with higher BDEs and weaker chelators relative to flavonols) (Kruk et al. 2022). In flavanones, the absence of conjugation limits radical stabilization, although *ortho*‐dihydroxyl patterns on ring A can partially recover antiradical capacity (Amic et al. 2007). In addition, their lower polarity compared to other flavonoids may favor different partitioning behavior, which can be advantageous or detrimental depending on the location of oxidation reactions within the food system.

Isoflavones, another category of flavonoids, differ from other flavonoids in their skeletal topology: Ring B is linked to C3 of ring C rather than C2 (Bešlo et al. 2023; Câmara et al. 2021). This structural modification confers similarity to estradiol, resulting in pseudo‐hormonal properties and determining specific molecular targets (e.g., estrogen receptors) (Manach et al. 2004). SAR studies highlight that greater molecular planarity is associated with higher antioxidant efficiency (Rudrapal et al. 2024). From a food system perspective, this altered geometry may also influence molecular interactions and partitioning behavior, affecting their functional performance compared to other flavonoid subclasses.

Lastly, considering the flavonoid group, the anthocyanins and anthocyanidins: These compounds typically occur as flavylium cations under acidic pH, conferring electronic stability due to 3‐OH and multiple hydroxyl groups on ring B (Ji et al. 2024). Glycosylation at C3 promotes copigmentation, a supramolecular phenomenon that stabilizes the flavylium cation and protects the molecule against thermal and photooxidative degradation, thereby broadening antioxidant functionality in aqueous matrices (J. Deng et al. 2018; Vinha et al. 2018). Additionally, copigmentation stabilizes pigment color by forming noncovalent supramolecular complexes with other phenolics and, occasionally, with metal ions (Vinha et al. 2018). However, their stability is highly dependent on pH, temperature, and the presence of oxygen, and structural transformations (e.g., hydration, cleavage, or polymerization) during processing and storage may significantly reduce their antioxidant effectiveness in real food systems.

#### 3.1.2 Stilbenes

Stilbenes are low‐molecular‐weight phenolics based on the C_6_–C_2_–C_6_ skeleton, in which two aromatic rings are linked by an ethylenic bridge (Câmara et al. 2021; Pecyna et al. 2020; Rasha and Khalid Mustafa 2025; Rudrapal et al. 2022). The *trans* form maintains planarity and full conjugation between the rings, a condition that supports electronic stabilization of the phenoxyl radical formed after hydrogen abstraction. *Cis* isomerization, favored by UV light, disrupts this conjugation and reduces antioxidant efficiency (Lang et al. 2024; Navarro‐Orcajada et al. 2023). Resveratrol (3,4′,5‐trihydroxystilbene) represents the structural archetype of the class. Its reactivity is associated with preferential deprotonation of 4′‐OH and semiquinone stabilization by resonance along the conjugated aromatic system (Lang et al. 2024; Navarro‐Orcajada et al. 2023; Pecyna et al. 2020). The C = C bridge functions as a delocalization axis, lowering the energy of the formed radical. This arrangement explains resveratrol's performance in oxidative systems where radical stabilization is the rate‐limiting step (Navarro‐Orcajada et al. 2023; Pecyna et al. 2020). However, in real food systems, the antioxidant performance of stilbenes is strongly influenced by their physicochemical environment and distribution within the matrix.

In addition, stilbenes are particularly sensitive to environmental factors such as light, oxygen, and temperature, which can induce isomerization (*trans*–*cis* conversion) and oxidative degradation during processing and storage. These transformations not only reduce their intrinsic antioxidant capacity but may also alter their partitioning behavior and interactions with other matrix components, thereby impacting their overall effectiveness in food systems.

Variation in the number and position of hydroxyls directly alters reactivity. Piceatannol, which contains an additional catechol motif on ring B, exhibits lower BDE and greater radical stability, yielding higher efficiency than resveratrol (Navarro‐Orcajada et al. 2023). Conversely, methoxylation reduces hydrogen‐donating and electron‐transfer capacity; pterostilbene exemplifies this tendency, with lower intrinsic activity due to reduced available electron density (Lang et al. 2024). However, methoxylation also increases lipophilicity, which may enhance partitioning into lipid phases and improve stability against oxidation and metabolic degradation, partially compensating for the reduced intrinsic reactivity in different food matrices.

#### 3.1.3 Lignans

Lignans derive from the dimerization of two phenylpropanoid (C_6_–C_3_) units via C8–C8′ coupling, forming the C_6_–C_3_–C_3_–C_6_ skeleton that distinguishes the class from neolignans (Câmara et al. 2021; Plaha et al. 2022; Rasha and Khalid Mustafa 2025). Structural diversity arises from hydroxylation/methoxylation patterns of the rings, the presence of lactones (e.g., dibenzylbutyrolactones), and glycosylation; SECO, SDG, pinoresinol, and matairesinol are among the most‐studied representatives (Lang et al. 2024; Plaha et al. 2022). Mechanistically, antioxidant activity derives primarily from the hydrogen‐transfer capacity of phenolic groups, because the dimerized system does not present extended electronic conjugation between rings, limiting semiquinone stabilization compared with flavonoids and stilbenes (Rosales and Fabi 2023; Rudrapal et al. 2024; L. X. Wang et al. 2022). Metal chelation tends to be modest because most lignans lack catechol or pyrogallol motifs; when present, these motifs increase affinity for Fe^3+^ and Cu^2+^, though they are less frequent in the class (Amic et al. 2007; Lang et al. 2024; Zeb 2020). Glycosylation, as observed in lignans like SDG, reduces acidity and the ease of phenolic deprotonation, diminishing direct reactivity, but increases physicochemical stability (Plaha et al. 2022). In food systems, these structural characteristics result in antioxidant behavior that is generally less immediate but potentially more stable over time.

Interactions with macromolecules further modulate lignan functionality. Compared to highly hydroxylated flavonoids, lignans tend to exhibit weaker binding to proteins and PSs, which may preserve the accessibility of phenolic groups but also reduce their retention in structured matrices. Moreover, their modest metal‐chelating capacity suggests that their antioxidant contribution in food systems may rely more on radical‐scavenging mechanisms than on inhibition of metal‐catalyzed oxidation pathways.

These forms act as metabolic precursors that, upon microbial conversion in the intestine, yield enterodiol and enterolactone, compounds with greater antioxidant activity due to the presence of free phenols and the electronic reorganization associated with the lactone ring (Plaha et al. 2022; Rudrapal et al. 2024). This transformation highlights that, although lignans may exhibit limited effectiveness within the food matrix itself, their contribution to oxidative balance may become more relevant after digestion, emphasizing the importance of considering both in situ functionality and post‐digestive bioactivity.

#### 3.1.4 Tannins

Considering the tannins, there is a structural distinction between condensed tannins (CTs) and hydrolyzable tannins (HTs). These groups entail differences in phenolic motifs that directly modulate antioxidant efficacy and TMC. HTs derive from esters of phenolic acids, especially gallic acid or HHDP, linked to a glucose core (Chiorcea‐Paquim et al. 2020; L. Zhang et al. 2023). The galloyl group is the most reactive motif for electron donation and metal complexation; tannic acid, for example, displays high galloyl density, favoring multiple coordination that often leads to precipitation when complexed with Fe^3+^ or Al^3+^ (Duan et al. 2025; L. Zhang et al. 2023). CTs (proanthocyanidins) are polymers of flavan‐3‐ol units linked by interflavan C–C bonds, with variable degrees of polymerization (Chiorcea‐Paquim et al. 2020; Watrelot and Norton 2020). The catechol motif on ring B is the principal site for redox reactions and chelation. The capacity to complex metals and neutralize reactive species increases with the hydroxylation level of ring B, following the order: tri‐hydroxylated > di‐hydroxylated (L. Zhang et al. 2023). Recent literature reinforces three additional points relevant here:

- Conformation‐ and polymerization‐dependent reactivity. CTs show greater oxidative stability and lower hydrolysis susceptibility than HTs. Effective antioxidant activity depends on access to phenolic sites, which is reduced in more extensive polymers (Watrelot and Norton 2020).
- Electrochemical mechanism and redox potentials. Voltammetric studies indicate that HTs exhibit lower oxidation potentials than CTs, consistent with the greater ease of oxidation of galloyl groups (Chiorcea‐Paquim et al. 2020). These data support the mechanistic distinction between the two classes and aid in predicting redox behavior in food matrices.
- Interaction with proteins and aggregation effects. CTs have a greater propensity to form protein–tannin aggregates owing to their polymeric architecture and relatively higher hydrophobicity. HTs interact via mechanisms more dominated by metal–tannin coordination (Watrelot and Norton 2020). Such differences influence the availability of phenolic sites and, therefore, the measurable antioxidant activity. In real food systems, these structural features result in highly matrix‐dependent antioxidant behavior.

Additionally, the high molecular weight and polymeric nature of CTs can restrict their mobility and diffusion within the food matrix, reducing their ability to reach sites of lipid oxidation, especially in heterogeneous systems. Conversely, HTs, due to their smaller molecular size and ester‐linked structure, may exhibit greater accessibility but also be more susceptible to hydrolysis and degradation during processing and storage.

Metal chelation by tannins may also have dual effects depending on the system. Although complexation with transition metals can inhibit pro‐oxidant catalytic cycles, the formation of insoluble metal–tannin complexes may lead to precipitation and removal from the reactive environment, thereby reducing their effective antioxidant contribution.

Therefore, the antioxidant performance of tannins in food systems is governed jointly by their intrinsic redox properties and by their tendency to interact, aggregate, and partition within complex matrices, which can either enhance or limit their functional effectiveness depending on the specific conditions.

#### 3.1.5 Phenolic Acids

Phenolic acids form the principal class of non‐flavonoid phenols and share a benzene ring substituted with a carboxyl group and hydroxyl or methoxy groups. The main category of phenolic acids is hydroxybenzoic acids and hydroxycinnamic acids. They derive from two basic cores: hydroxybenzoic acids (C_6_–C_1_) and hydroxycinnamic acids (C_6_–C_3_) (Câmara et al. 2021). Variation in hydroxylation pattern and extent of conjugation defines redox reactivity, influencing both HAT and metal chelation (Lang et al. 2024). In addition, their relatively low molecular weight and higher polarity compared to other PPs strongly influence their solubility, mobility, and distribution within food matrices, which are key determinants of their effective antioxidant performance.

Hydroxybenzoic acids exhibit simpler structures and less conjugation, which limits stabilization of the phenoxyl radical formed after HAT. Gallic acid is the exception due to its pyrogallol arrangement (3,4,5‐tri‐OH), which lowers the O–H bond energy and supports strong reducing activity (Câmara et al. 2021; Lang et al. 2024). However, their high polarity generally favors localization in the aqueous phase, which may limit direct interaction with lipid radicals in multiphase systems, thereby reducing their effectiveness in inhibiting lipid oxidation despite their high intrinsic reactivity.

In hydroxycinnamic acids, the Cα = Cβ double bond conjugated to the aromatic ring promotes effective radical delocalization, reducing the O–H bond dissociation enthalpy and increasing HAT efficiency (Zeb 2020). The presence of catechol units, as in caffeic acid (3′,4′‐diOH), intensifies this effect through resonance stabilization and formation of intramolecular hydrogen bonds (Rashmi and Negi 2020). Partial methoxylation, as in ferulic acid, decreases available electron density and reduces relative reactivity, though significant activity is retained (Rudrapal et al. 2024). Nevertheless, moderate increases in hydrophobicity associated with methoxylation may favor partitioning at interfaces or within lipid phases, potentially enhancing their functional effectiveness in certain food systems where oxidation occurs in non‐aqueous regions.

Metal chelation follows the structural pattern observed in other phenols: Catechol and pyrogallol motifs show higher affinity for Fe^3+^ and Cu^2+^, inhibiting steps associated with Fenton/Haber–Weiss chemistry (Belščak‐Cvitanović et al. 2018). Gallic acid forms stable ferric complexes, whereas caffeic acid acts as an efficient catecholate ligand. Compared with the literature, the following general order of reducing power is reported: gallic > caffeic > ferulic > vanillic (Zeb 2020). Some of these acids also appear as metabolites derived from intestinal degradation of anthocyanins, retaining relevant antioxidant capacity (Lang et al. 2024). In food systems, their relatively small size and high diffusivity may facilitate rapid interaction with reactive species but also make them more susceptible to leaching, degradation, or transformation during processing and storage. Therefore, their effectiveness is governed by a balance between intrinsic reactivity, mobility, and retention within the matrix.

### 3.2 Carotenoids

Carotenoids are lipophilic tetraterpenoids defined by a polyene chain with conjugated single and double bonds, usually terminated by cyclic or acyclic groups (Vinha et al. 2018). The presence or absence of oxygen differentiates carotenes (hydrocarbons such as lycopene and β‐carotene) from xanthophylls (lutein, zeaxanthin) (Zhuang et al. 2022). Their extensive conjugated system underpins redox reactivity, determining both quenching capacity and susceptibility to *cis*/*trans* isomerization. Due to their hydrophobic nature, carotenoids are predominantly located within lipid phases or associated with hydrophobic domains in food matrices, which strongly influences their accessibility to reactive species and their overall antioxidant performance.

Antioxidant activity derives primarily from singlet‐oxygen (^1^O_2_) quenching and interception of peroxyl radicals. Quenching of ^1^O_2_ may proceed physically, with non‐reactive energy transfer that preserves the carotenoid, or chemically, in which the polyene is oxidized. Efficiency correlates directly with the number of conjugated double bonds (*n*): Second‐order rate constants for ^1^O_2_ quenching rise from ∼10^9^ M^−1^ s^−1^ for short‐chain carotenoids (*n* ≈ 7) to 1.3 × 10^10^ M^−1^ s^−1^ for β‐carotene (*n* = 11) and 3.1 × 10^10^ M^−1^ s^−1^ for lycopene (*n* = 11, acyclic), with astaxanthin reaching ∼2.4 × 10^10^ M^−1^ s^−1^, values originally established in the seminal quantitative study of Di Mascio et al. (1989) and reaffirmed in recent compilations (Meléndez‐Martínez et al. 2022). These rate constants are about three orders of magnitude higher than those of tocopherols (*k*_*q*(^1^O_2_) ∼10^8^ M^−1^ s^−1^) and six orders higher than those of water‐soluble flavonoids (∼10^6^–10^7^ M^−1^ s^−1^), mechanistically justifying the non‐substitutable role of carotenoids in ^1^O_2_‐rich microenvironments (chlorophyll‐containing foods, photo‐exposed lipid phases).

The unsaturated chain also makes these compounds sensitive to *E*/*Z* photoisomerization, intensified by light, heat, and acidity (Fotouhi et al. 2025). The all‐*trans* conformation is more stable, yet *cis* isomers can exhibit distinct behavior. In lycopene, the 5*Z*, 9*Z*, 13*Z*, and 15*Z* forms account for more than half of the circulating pool and may be preferentially absorbed or metabolized (Han et al. 2012; Meléndez‐Martínez et al. 2023). This isomerization influences stability, reactivity, and technological performance (Fotouhi et al. 2025). In food processing and storage, these structural transformations can lead to significant losses in antioxidant capacity, particularly under high temperatures, light exposure, and oxygen availability.

Antioxidant properties vary with polarity. Nonpolar carotenes are located in the hydrophobic region of membranes, whereas xanthophylls, bearing hydroxyl or carbonyl groups, anchor at the lipid/water interface, modulating supramolecular organization and resistance to oxidation (Han et al. 2012). This spatial distribution favors interfacial synergies with α‐tocopherol and ascorbate, in which xanthophylls act at the aqueous–lipid boundary where oxidative initiation is concentrated, whereas carotenes operate within the bulk lipid phase; the kinetic basis of these synergies is examined in the regeneration‐cycle paragraph below (Han et al. 2012).

β‐Carotene reacts with peroxyl radicals via reversible radical‐adduct formation (β‐Car + ROO• ⇌ ROO–β‐Car•). Quantitatively, the rate constant for ROO• addition to β‐carotene is several orders of magnitude lower than for ^1^O_2_ quenching (*k*_*q*(^1^O_2_) ≈ 1.3 × 10^10^ M^−1^ s^−1^; Section 3.2), so under conditions where the ROO• flux outweighs the ^1^O_2_ flux, elevated *p*O_2_, prolonged storage, photo‐stressed lipid phases, and the peroxyl‐radical pathway, with its *p*O_2_‐ and concentration‐dependent inversion, become rate‐limiting (El‐Agamey and McGarvey 2003; Zhuang et al. 2022).

Whether the chain‐breaking or the propagation regime dominates is set by the relative rates of two competing reactions of the β‐carotenyl adduct: unimolecular decomposition back to parent β‐carotene (rate *k*_–O_2_), and bimolecular reaction with ^3^O_2_ to generate a propagating Car–OO• peroxyl radical (rate *k*_O_2_[^3^O_2_]). At low *p*O_2_, *k*_–O_2_ ≫ *k*_O_2_[^3^O_2_], and the system is chain‐breaking; at higher *p*O_2_, *k*_O_2_[^3^O_2_] outcompetes *k*_–O_2_, and the same molecule propagates the chain. This *p*O_2_‐dependent inversion was located by Burton and Ingold (1984) at a *p*O_2_ clearly below ambient air and has been repeatedly reaffirmed (Zhuang et al. 2022). A second, photochemical route reinforces the inversion under light: Triplet β‐carotene (^3^β‐carotene) reacts non‐concertedly with ^3^O_2_ to yield β‐carotene endoperoxides (βCar‐EPOs), whose photo‐induced breakdown initiates a free‐radical avalanche that re‐couples carotenoid chemistry to lipid‐phase propagation. The pro‐oxidant trajectory of carotenoids is therefore a matrix‐defined kinetic boundary condition (*p*O_2_, light flux, co‐antioxidant pool), not a chemical property of the molecule (Dávalos et al. 2018; Zbyradowski et al. 2022).

This kinetic framework is consistent with the ATBC and CARET supplementation outcomes, in which 20 mg/day β‐carotene (often co‐administered with retinol) was associated with increased lung‐cancer incidence in smokers whose lung tissue *p*O_2_ and oxidative environment favor the pro‐oxidant regime (Dávalos et al. 2018). The antioxidant → pro‐oxidant transition has been mapped quantitatively by microplate kinetic assays (Zhuang et al. 2022): In cellular and lipid model systems, lycopene affords full radiation protection at 0% O_2_ but null protection at 100% O_2_, locating the antioxidant‐to‐pro‐oxidant crossover at a *p*O_2_ clearly below ambient air. Likewise, β‐carotene quenching efficiency falls monotonically with rising *p*O_2_ above ∼150 Torr (the O_2_ pressure of normal air) (Edge and Truscott 2018). The transition concentration is therefore matrix‐ and *p*O_2_‐dependent, but the local lipid‐phase concentrations required are reached only under high‐dose supplementation, not under habitual dietary exposure (typical plasma β‐carotene <700 nmol/L (Böhm et al. 2021)), consistent with the absence of an adverse signal in dietary cohort studies and its emergence at supplemental ≥20 mg/day in the ATBC and CARET trials.

The pro‐oxidant transition described above is partially counterbalanced by regeneration cycles when reducing co‐antioxidants are colocalized with carotenoid radical species. Pulse‐radiolysis and laser‐flash‐photolysis data have established that one‐electron‐oxidized carotenoid radical cations (Car•^+^) of β‐carotene, lycopene, zeaxanthin, canthaxanthin, and astaxanthin are reduced by ascorbate at second‐order rate constants in the 10^6^–10^8^ M^−1^ s^−1^ range, with the precise value depending on carotenoid protonation state and aggregation environment (Edge and Truscott 2018). These rates are several orders of magnitude higher than the O_2_‐addition step to neutral β‐carotenyl radicals, providing the kinetic basis for ascorbate‐mediated rescue from the pro‐oxidant trajectory whenever ascorbate is locally available.

Tocopherols and tocotrienols can analogously regenerate carotenes from neutral carotene radicals in lipid phases, an interaction that mechanistically underlies the carotene–vitamin E synergy observed in liposomes and in lipid‐rich matrices and that locates carotenoids upstream of tocopherols in the antioxidant hierarchy of biological membranes (Edge and Truscott 2018; Han et al. 2012).

Mechanistically, the antioxidant‐versus‐pro‐oxidant outcome is set by the competition between regeneration of the one‐electron‐oxidized carotenoid radical cation (Car•^+^) by a co‐antioxidant and O_2_ addition to the neutral carotenyl radical to form a propagating Car–OO•:

$$k\_\mathrm{reg}[\mathrm{co}\;-\;\mathrm{AO}x]\to \mathrm{Car}{•}^{+}\to \;\mathrm{Car}(\mathrm{chain}\;-\;\mathrm{breaking})$$

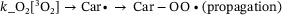




Pulse‐radiolysis and laser‐flash data place the second‐order rate constants for ascorbate‐mediated reduction of β‐carotene, lycopene, zeaxanthin, canthaxanthin, and astaxanthin radical cations at 10^6^–10^8^ M^−1^ s^−1^, against *k*_O_2_ ≈ 10^5^ M^−1^ s^−1^ for ^3^O_2_ addition to the neutral β‐carotenyl radical (Edge and Truscott 2018). The kinetic asymmetry is therefore three to four orders of magnitude in favor of regeneration, and even sub‐millimolar local ascorbate suppresses propagation provided ^3^O_2_ tension stays below the inversion threshold. In ascorbate‐ and tocopherol‐replete matrices at low *p*O_2_, *k*_reg dominates and the system remains chain‐breaking; in late‐storage refined oils, frying media after several cycles, or dispersed‐oil systems with high oxygen ingress, the local reductant pool collapses and *k*_O_2_ [^3^O_2_] outcompetes *k*_reg[co‐AOx], shifting the same nominal carotenoid dose toward propagation. This competition mirrors the *k*_reg–*k*_TMP framework developed in Section 3.3 for tocopherols and places carotenoid and tocopherol radical chemistry under a single matrix‐defined kinetic boundary condition (Edge and Truscott 2018; Mertens et al. 2022; Zhuang et al. 2022).

The *p*O_2_‐ and concentration‐dependence of this transition translates directly into food‐relevant degradation kinetics: In tomato pulp at 80–110°C, all‐*E*‐lycopene degradation follows first‐order kinetics with an Arrhenius activation energy of 22.7 kJ/mol, and lycopene retention drops to 72%, 61%, and 43% after 4 h at 80°C, 95°C, and 110°C, respectively, with β‐carotene showing biphasic, faster degradation in the same matrix (Sevindik Baç et al. 2023). In gac aril paste, lycopene exhibits substantially shorter half‐lives at 90°C (5.75 h) than at 70°C (12.81 h) and faster first‐order rate constants (*k* = 12.05 vs. 5.41 × 10^2^ h^−1^), with β‐carotene degrading more slowly under the same conditions (*t*_1_/_2_ 9.48–20.2 h) (Syawalluddin et al. 2024). These compound‐ and matrix‐specific kinetics explain why thermal windows that protect lycopene do not protect β‐carotene equivalently and why pro‐oxidant behavior is more readily entered for β‐carotene under oxygen‐replete, light‐exposed conditions. The translational corollary is that the high‐dose, high‐*p*O_2_ regimes that triggered adverse signals in supplementation trials (ATBC, CARET) are not produced by habitual dietary intake of carotenoid‐rich foods, in which plasma β‐carotene typically remains below 700 nmol/L and lycopene plasma concentrations remain below 1 µmol/L even in tomato‐rich diets (Böhm et al. 2021). Food‐system pro‐oxidant risk, therefore, arises principally during processing, fortified‐product formulation, and storage, not during consumption of unmodified plant‐based foods.

### 3.3 Tocopherols and Tocotrienols

Vitamin E encompasses eight liposoluble compounds, four tocopherols (α, β, γ, δ) and four tocotrienols, that share a chromanol ring with a phenolic hydroxyl linked to a C_16_ side chain (Muñoz and Munné‐Bosch 2019; Szewczyk et al. 2021). Tocopherols possess a saturated phytyl chain, whereas tocotrienols possess an isoprenoid chain with three double bonds. The α‐δ variation derives from the number and position of methyl groups on the chromanol ring (Barouh et al. 2022). Due to their amphiphilic character, these compounds are typically located at the lipid–water interface or within lipid phases, where they play a critical role in protecting unsaturated lipids from oxidative degradation.

Antioxidant activity results from hydrogen donation (HAT) from the phenolic group, interrupting lipid peroxidation by converting peroxyl radicals to hydroperoxides and generating the tocopheroxyl radical (TocO•) (Muñoz and Munné‐Bosch 2019; Szewczyk et al. 2021). Intrinsic efficiency correlates with the O–H bond‐dissociation enthalpy (BDE) and radical resonance stabilization, influenced by the ring's methylation pattern. In homogeneous systems, reactivity typically follows α > β ≈ γ > δ for both tocopherols and tocotrienols (Barouh et al. 2022). However, in real food systems, antioxidant effectiveness depends on intrinsic reactivity together with spatial distribution and regeneration capacity within the matrix. Their positioning at lipid interfaces enables efficient interception of peroxyl radicals, but their activity is strongly influenced by co‐antioxidants that regenerate the tocopheroxyl radical back to its active form.

Tocotrienols exhibit comparable or superior reactivity in lipid media due to the greater mobility conferred by their unsaturated side chains, although the radical mechanism is identical to that of tocopherols (Szewczyk et al. 2021). This enhanced mobility may improve their ability to diffuse within lipid domains and access reactive sites, which can be particularly relevant in complex food matrices with heterogeneous lipid distribution. Under elevated O_2_ partial pressure, in Fe/Cu‐rich matrices, or at high local α‐tocopherol concentrations, α‐TocO• can shift from termination to a propagation step known as tocopherol‐mediated peroxidation (TMP), in which it abstracts an H from an additional lipid substrate rather than being regenerated.

Whether the chain‐breaking or the TMP pathway dominates is set by the relative rates of two competing α‐TocO• reactions:

$$\begin{array}{ccc} & & k\_\mathrm{reg}\left[\mathrm{co}-\mathrm{AOx}\right]\;\to \alpha -\mathrm{TocO}•\to \alpha \\ & & -\mathrm{TocOH}\;\left(\mathrm{regenerated},\;\mathrm{chain}-\mathrm{breaking}\right) \end{array}$$

$$\begin{array}{ccc} & & k\_\mathrm{TMP}\left[\mathrm{LH}\right]\;\;\to \;\;\alpha -\mathrm{TocO}•\to \;\alpha -\mathrm{TocOH}\;\\ & & +\;L•\;\left(\mathrm{propagation}\;\mathrm{via}\;\mathrm{TMP}\right) \end{array}$$

Regeneration is thermodynamically downhill: The standard one‐electron reduction potential of α‐TocO•/α‐TocOH (*E*° ≈ +480 − 500 mV vs. NHE) lies above those of the ascorbate radical/ascorbate couple and of the *o*‐semiquinone/catechol couples of catechol‐ and pyrogallol‐bearing flavonoids. Quantitatively, EPR‐based liposomal kinetics place *k*_reg by ascorbate at ∼2 × 10^5^ M^−1^ s^−1^ at the lipid–water interface (Mertens et al. 2022). Catechol‐ and pyrogallol‐bearing flavonoids (quercetin, myricetin) proceed at comparable rates, whereas saturated 2,3‐bond flavonoids (taxifolin, catechin) cannot regenerate α‐TocO• competitively because their semiquinone resonance is interrupted (Bayram et al. 2023; Robichon et al. 2026). The *k*_TMP for H‐abstraction from polyunsaturated substrates is several orders of magnitude slower under the same conditions; this asymmetry is the kinetic basis for why sub‐stoichiometric reductant pools sustain the chain‐breaking regime. Consequently, in matrices where local co‐antioxidant concentration exceeds the polyunsaturated‐lipid substrate concentration, regeneration outcompetes TMP. As local co‐antioxidant pool collapses (high‐temperature processing, prolonged storage, oxygen‐replete bulk lipid), the same α‐tocopherol pool shifts into TMP. The TMP transition is therefore a matrix‐defined kinetic boundary condition, not an intrinsic property of the tocopherol class (Barouh et al. 2022; Bayram and Decker 2024; Mertens et al. 2022).

This kinetic framework explains why structurally similar flavonoids can show opposite interaction signs with α‐tocopherol under identical emulsion conditions: Taxifolin (saturated 2,3‐bond, no extended *o*‐semiquinone resonance) cannot regenerate α‐TocO• at a rate competitive with TMP, whereas myricetin produces synergism indices of 2.44–3.63 in the same matrix (Bayram et al. 2023). The same regeneration‐versus‐propagation logic applies to carotenoid radical chemistry described in Section 3.2, where Car•^+^ reduction by ascorbate or tocopherols competes with O_2_ addition to a propagating Car‐OO• radical.

Quantitative interfacial‐kinetics data in O/W emulsions corroborate this framework and reveal that the sign of the antioxidant interaction is set by pH and emulsifier identity, not by chemical identity alone. In stripped soybean O/W emulsions at pH 7.0, α‐tocopherol combined with myricetin produced interaction indices of 3.00–3.63 for lipid hydroperoxides and 2.44–3.00 for hexanal at 2:1 and 1:1 ratios, attributable to myricetin‐mediated regeneration of the α‐tocopheroxyl radical; at pH 4.0, the same pair was antagonistic, and the structurally near‐identical taxifolin (saturated 2,3‐bond, no extended *o*‐semiquinone resonance) was antagonistic at both pH values while still contributing to iron‐related pro‐oxidant chemistry (Bayram and Decker 2023). Phospholipid composition is a second, independently controllable lever: Mixed tocopherols (300 µmol/kg oil) with phosphatidylethanolamine‐enriched lecithin (1500 µmol/kg oil) extended hydroperoxide and hexanal lag phases by 3 days at pH 7 in O/W emulsions; in stripped bulk soybean oil, 50 µmol/kg oil α‐tocopherol with 1000 µmol/kg oil phosphatidylethanolamine‐enriched lecithin extended the lag phases by 5 and 4 days, respectively (Culler et al. 2022). The same chemical pair therefore operates as a synergist or antagonist depending on local pH, suggesting that pair‐wise interaction indices reported in the literature must be interpreted as configuration‐specific outputs, not as transferable formulation rules.

Beyond pair‐wise interaction indices, the kinetic signature of α‐tocopherol depletion has recently been developed as an early‐warning shelf‐life indicator. In stripped soybean oil at 50°C, early‐phase α‐tocopherol decay fits first‐order kinetics whose rate constant correlates with the subsequent hexanal lag phase, enabling shelf‐life prediction from accelerated data on a time‐scale of hours rather than weeks (Parra‐Escudero et al. 2025). Machine‐learning‐guided modeling of the α‐tocopherol/myricetin system has since identified a parsimonious autocatalytic differential equation that generalizes across a 10‐fold concentration range (10–100 µM) and reproduces the regeneration–depletion dynamics without a priori assumptions about reaction order (Parra‐Escudero et al. 2025). Consistent with this depletion‐rate framework, in non‐stripped soybean and corn oils, α‐tocopherol is fully depleted by the end of the oxidation lag phase, whereas (γ + β)‐ and δ‐tocopherol concentrations remain at >70% and 65%, respectively, providing a tocopherol‐isoform‐specific kinetic readout that can be used to triangulate shelf‐life predictions in commodity oils (Cantele et al. 2025). Independent validation in a Crocin‐bleaching‐assay–Weibull framework in fish‐oil O/W systems confirmed that α‐tocopherol–carnosic acid and α‐tocopherol–myricetin combinations are concentration‐dependent synergists, α‐tocopherol–dieckol is synergistic at equimolar ratios, and α‐tocopherol–ascorbic acid is antagonistic under those emulsion conditions, a configuration‐specific result that contradicts the often‐generalized claim of universal ascorbate–tocopherol synergy (Robichon et al. 2025).

More recent work coupling cyclic voltammetry with the Weibull‐interactions‐modeling‐CAT spectrophotometric assay has shown that the synergy of α‐tocopherol with quercetin and of γ‐tocopherol with curcumin is dose‐windowed: Synergistic effects are strongest at low tocopherol concentrations (0.2 µM in emulsion, ≈380 ppm in oil) and high PP‐to‐tocopherol molar ratios (3:1) and are attenuated at higher tocopherol levels (0.6 µM in emulsion, ≈1140 ppm in oil), an attenuation that the authors attribute to the emergence of pro‐oxidant activity at higher chromanol load. Acidic conditions reduced tocopherol pro‐oxidation and modified the curcumin and quercetin contributions, and ferrous‐ion supplementation accelerated overall oxidation without altering the sign of the synergy (Robichon et al. 2026). These voltammetric data shift the mechanistic interpretation of α‐tocopherol regeneration from a qualitative “regeneration occurs” to a quantitative dose‐ and ratio‐dependent operating window, defining the concentration envelope within which tocopherol–PP partnerships act as net antioxidants.

### 3.4 Alkaloids and Other Phytochemicals (Tyrosol, Hydroxytyrosol, and Vanillin)

Compounds, such as alkaloids, tyrosol, hydroxytyrosol, and vanillin, represent subclasses of structurally simple phytochemicals that exert antioxidant activity mainly via HAT or SET, with reactivity conditioned by the presence of phenolic, catecholic, or heteroaromatic rings (Bertelli et al. 2020; Martínez et al. 2018; Olatunde et al. 2022). Due to their relatively low molecular weight and structural simplicity, these compounds typically exhibit high solubility and mobility within food matrices, which can favor rapid interaction with reactive species but also increase susceptibility to diffusion, loss, or degradation during processing and storage. Alkaloids form a heterogeneous class of nitrogen‐containing compounds whose redox reactivity depends on conjugation between heterocyclic nitrogen and aromatic systems. Isoquinoline structures, such as berberine, exhibit the ability to stabilize radicals formed via SET, although the absence of catechol motifs limits activity against peroxyl species when compared with PPs (Jiang et al. 2026). In addition, the polarity and structural diversity of alkaloids may result in variable partitioning behavior, influencing their accessibility to oxidation sites depending on the composition and physical state of the food matrix.

Tyrosol (TYR) and hydroxytyrosol (HXT) are C6–C2 phenols, distinguished by the catechol motif present in HXT (Bertelli et al. 2020; Olatunde et al. 2022). This modification reduces the energy required for hydrogen abstraction and stabilizes the phenoxyl radical through electronic delocalization, thereby increasing antioxidant efficiency compared with TYR (Dávalos et al. 2018; Martínez et al. 2018). Their relatively high polarity favors localization in aqueous phases, which may limit their direct effectiveness in lipid oxidation systems; however, this same characteristic allows them to act efficiently as secondary antioxidants or regenerators of other antioxidant species at interfaces or in aqueous domains.

Finally, vanillin is a C6–C1 phenolic aldehyde bearing a phenolic hydroxyl group conjugated to a methoxy and a formyl substituent (Moradi 2022; Olatunde et al. 2022). Its activity derives from hydrogen donation by the phenolic group and radical stabilization by resonance; additionally, vanillin can undergo oxidative dimerization in SET systems, producing redox‐stable intermediates that broaden its radical‐neutralization capacity. The aldehyde carbonyl contributes to moderate metal chelation, although inferior to catecholic phenols (H. Deng et al. 2024; Olatunde et al. 2022). In food systems, vanillin may interact with proteins and other matrix components via its aldehyde group, thereby influencing both its retention and antioxidant functionality. However, its relatively moderate reactivity compared to PPs suggests that its contribution is often complementary rather than dominant in controlling oxidative processes.

### 3.5 Integrative Perspective on Antioxidant Structure–Function Relationships in Food Matrices

A comparative summary of the main structural classes, dominant mechanisms, and matrix‐dependent limitations is presented in Table 1. The classes of plant‐based antioxidants reviewed in Section 3 share a common organizing principle: Their effectiveness in food systems depends on how their intrinsic reactivity (set by hydroxylation pattern, conjugation, and redox potential) is constrained by partitioning, mobility, and interactions inside the matrix. Highly reactive PPs, such as flavonoids and phenolic acids, may be limited by preferential partitioning into aqueous phases or by binding to proteins and PSs, reducing their accessibility to lipid oxidation sites. In contrast, more lipophilic compounds, including carotenoids, tocopherols, and tocotrienols, are better positioned to act within lipid domains or at interfaces but may exhibit lower stability or pro‐oxidant behavior under specific conditions, such as high oxygen availability or elevated concentrations. Polymerization, as observed in tannins, and structural simplicity, as in small phenolics and alkaloids, further illustrate how accessibility, diffusion, and interaction with matrix components can either enhance or restrict antioxidant functionality. Antioxidant effectiveness in food systems is therefore set jointly by intrinsic reactivity and by matrix‐dependent variables: phase distribution, molecular interactions with biopolymers, environmental conditions, and processing history. Predictive use of antioxidants in formulation work consequently requires both chemical characterization of the active compound and explicit description of the food matrix in which it will function.

**TABLE 1:** Comparative overview of major classes of plant‐based antioxidants: structural features, dominant mechanisms, and limitations in food systems.

Class	Key structural features	Dominant antioxidant mechanisms	Typical matrix localization	Main limitations in food systems
**Flavonoids**	C_6_–C_3_–C_6_ skeleton; hydroxylated aromatic rings; Bors criteria (catechol, C2=C3, 4‐oxo)	HAT, SET, SPLET, metal chelation	Mainly aqueous phase; limited presence at lipid interfaces	Limited access to lipid oxidation sites; binding to proteins/polysaccharides reduces reactivity
**Stilbenes**	C_6_–C_2_–C_6_ structure; conjugated ethylenic bridge; planarity (*trans* form)	HAT, SET; radical stabilization via conjugation	Interface or partially lipid phase	Susceptibility to isomerization and oxidation; performance depends on stability and partitioning
**Lignans**	Dimeric C_6_–C_3_–C_3_–C_6_ structure; limited conjugation; often glycosylated	HAT (moderate); weak metal chelation	Predominantly aqueous phase	Low intrinsic reactivity; limited access to lipid radicals; activity often more relevant post‐digestion
**Tannins (CTs and HTs)**	Polymeric phenolics; multiple hydroxyl groups; galloyl or catechol motifs	HAT, SET, strong metal chelation	Interact with proteins; often form aggregates	Reduced accessibility due to aggregation; limited diffusion; precipitation with metals
**Phenolic acids**	C_6_–C_1_ or C_6_–C_3_ structures; varying hydroxylation and conjugation	HAT, SET, metal chelation (catechol/pyrogallol)	Mainly aqueous phase	Limited effectiveness in lipid systems; high mobility may lead to loss or degradation
**Carotenoids**	Polyene chain with conjugated double bonds; lipophilic	^1^O_2_ quenching, peroxyl radical scavenging	Lipid phase or lipid–water interface	Susceptible to oxidation and isomerization; may become pro‐oxidant under high O_2_
**Tocopherols and tocotrienols**	Chromanol ring with phenolic OH; hydrophobic side chain	HAT; chain‐breaking antioxidant; regeneration cycles	Lipid phase and lipid–water interface	Activity depends on regeneration; may exhibit pro‐oxidant behavior under certain conditions
**Alkaloids and simple phenolics (tyrosol, hydroxytyrosol, vanillin)**	Low molecular weight; simple phenolic or heterocyclic structures	HAT, SET (moderate)	Mainly aqueous phase; high mobility	Lower intrinsic activity; rapid diffusion and loss; often act as secondary antioxidants

*Note*: This table focuses on major phytochemical antioxidant classes. Bioactive peptides derived from enzymatic hydrolysis of plant proteins and certain antioxidant polysaccharides are additional contributors to the antioxidant capacity of plant‐based food systems (see Section 6) but are not the primary focus of this review. Rankings in this table reflect intrinsic chemical reactivity under homogeneous‐solution conditions. In real food matrices and in vivo, the ranking can be attenuated, inverted, or inverted‐then‐restored by (i) partitioning between aqueous, interfacial, and lipid pseudophases (Losada‐Barreiro et al. 2024); (ii) pH‐dependent catechol autoxidation generating Fe^2+^ and superoxide at neutral‐to‐alkaline pH (Bayati and Poojary 2025); (iii) redox‐potential mismatches that convert a potential regenerator into an antagonist (Bayram and Decker 2024); and (iv) gut–microbial biotransformation that dissociates the systemic metabolite from the parent compound (Parmenter et al. 2023; Pidgeon et al. 2025). This table should therefore be read as the upper bound of performance under idealized conditions.

Abbreviations: CTs, condensed tannins; HAT, hydrogen‐atom transfer; HTs, hydrolyzable tannins; SET, single‐electron transfer; SPLET, sequential proton loss electron transfer.

Although the SAR rules summarized in Table 1 are robust in homogeneous solution, the translational literature reveals several systematic contradictions that deserve explicit acknowledgment. First, the Bors criteria predict that quercetin should outperform myricetin as a radical scavenger, yet in O/W emulsions myricetin produces interaction indices of 2.44–3.63 with α‐tocopherol at pH 7.0, whereas the closely related taxifolin is antagonistic under the same conditions, because interfacial localization combined with the ability to regenerate the tocopheroxyl radical (not the DPPH value) determines performance (Bayram et al. 2023).

Second, catechol‐bearing phenolics are ranked as potent antioxidants by FRAP/ABTS/DPPH but can become net pro‐oxidants at neutral‐to‐alkaline pH, where catechol autoxidation generates superoxide and regenerates Fe^2+^, accelerating Fenton chemistry and driving protein and lipid oxidation even in model systems (Bayati and Poojary 2025; Parmenter et al. 2023).

Third, and most critically for the readers of this journal, SAR rankings based on parent compounds offer limited predictive power for in vivo outcomes once the gut microbiota catabolizes the molecule: For flavan‐3‐ols, intact parent compounds contribute only marginally to systemic exposure, whereas microbial phenyl‐γ‐valerolactones dominate plasma and urinary metabolite profiles, with two specific PVLs accounting for more than 75% of urinary recovery (Parmenter et al. 2023) and mean flavan‐3‐ol bioavailability of 31% ± 23% across 49 human studies (Di Pede et al. 2023).

For anthocyanins, the clinical response measured in 24‐week randomized trials of adults at dementia risk (LDL *η* ^2^ = 0.078, *p* = 0.015; CRP *η* ^2^ = 0.417, *p* < 0.001) emerged despite limited parent‐compound bioavailability, likely through metabolite‐ or systems‐level mechanisms (Borda et al. 2025). Three classes of well‐documented inconsistencies in the recent food‐chemistry literature delimit the predictive domain of SAR in food systems and warrant explicit enumeration:

- Closely related flavonoids show opposite signs of interaction with α‐tocopherol under identical emulsion conditions. Myricetin produces synergism indices of 2.44–3.63 for hexanal and lipid hydroperoxide formation in stripped soybean O/W emulsions at pH 7.0; taxifolin, differing essentially in its saturated 2,3‐bond and absent extended *o*‐semiquinone resonance, is antagonistic in the same matrix, although DPPH ranking predicts similar performance (Bayram et al. 2023);
- The α‐tocopherol/ascorbate regeneration synergy is configuration‐dependent, not universal. In fish‐oil O/W emulsions, ascorbate becomes antagonistic to α‐tocopherol under conditions where carnosic acid, dieckol, and myricetin remain synergistic, and γ‐tocopherol synergizes with curcumin, whereas α‐tocopherol does not under voltammetrically identical conditions (Robichon et al. 2025, 2026). The sign of the interaction is set by interfacial localization, ferrous‐ion availability, and pH, not by chemical identity;
- Catechol‐bearing PPs can convert from antioxidants to pro‐oxidants with a single pH change. At pH < 6, they form bidentate Fe^3+^ complexes that suppress Fenton chemistry; at neutral‐to‐alkaline pH the same catechols undergo autoxidation, generating O_2_•^−^ via *o*‐semiquinone intermediates and reducing Fe^3+^ to Fe^2+^, accelerating Fenton chemistry and driving protein and lipid oxidation in model systems (Bayati and Poojary 2025; Parmenter et al. 2023), and;
- For ellagitannin‐ and isoflavone‐derived bioactives, in vivo SAR is dictated by host‐microbiota metabotype, not by the parent compound. In controlled trials of pomegranate ellagitannins, urolithin‐metabotype‐B participants, but not metabotype‐A or non‐producer participants, showed significant improvements in apoB and oxidized‐LDL despite identical food intake (García‐Villalba et al. 2022; Iglesias‐Aguirre et al. 2023). The molecular basis has now been resolved at the operon level (*Enterocloster* ucd) for ellagic‐acid catabolism (Pidgeon et al. 2025).

These four classes do not invalidate SAR; they define its boundary conditions. SAR rules derived in homogeneous solution are quantitatively reliable in domain (i) below, drop in predictive power in domains (ii) and (iii), and can fail qualitatively in domain (iv), a hierarchy made explicit in the next paragraph.

In homogeneous solution, SAR rules (Bors criteria, chromanol methylation patterns) capture most of the variance in DPPH/ABTS/FRAP rankings (Bešlo et al. 2023). In oil‐in‐water emulsions, the predictive power of homogeneous‐phase SAR collapses, as illustrated by the three contradictions enumerated above (Bayram et al. 2023; Robichon et al. 2025, 2026). In vivo, the predictive power collapses further because the systemic metabolite (phenyl‐γ‐valerolactone, urolithin A, enterolignan) is often structurally unrelated to the parent compound, and the metabolite‐producing capacity is determined by host microbiota rather than by food composition (Pidgeon et al. 2025).

A rigorous use of SAR in food science therefore requires declaring, for each mechanistic claim, the operating domain. Predictive reliability decreases monotonically across four domains:

- Homogeneous solution: Bors criteria, BDE values, and chromanol methylation rules capture most of the variance in DPPH/ABTS/FRAP rankings;
- Bulk oils and model emulsions: Predictive power drops because partitioning, regeneration competence, and local *p*O_2_ become rate‐limiting, and closely related structural pairs can show opposite interaction signs (myricetin vs. taxifolin with α‐tocopherol);
- Real multicomponent foods: Protein/PS binding, transition‐metal availability and processing history can invert the ranking;
- In vivo: The metabolite that reaches human plasma is often structurally unrelated to the parent (phenyl‐γ‐valerolactones, urolithins, enterolignans), and metabolite‐producing capacity is host‐microbiota‐dependent rather than food‐composition‐dependent (Iglesias‐Aguirre et al. 2023; Pidgeon et al. 2025).

The same SAR rule that holds quantitatively in domain (i) can, therefore, fail qualitatively in domain (iv); reviews and primary papers that conflate domains overstate the translational value of in vitro rankings.

## 4 Impact of Processing Technologies on Antioxidant Stability in Plant‐Based Food Matrices

Plant‐based antioxidants do not behave uniformly in food systems; their stability results from a combination of properties inherent to the compounds themselves and external conditions imposed by the food matrix and storage environment (Suhag et al. 2025; Wijesekara and Xu 2024). Intrinsic factors, such as molecular structure, degree of conjugation, and localization within plant tissues, influence the reactivity and accessibility of these compounds, whereas plant species, ripening stage, and cultivation conditions further modulate their initial profiles (Gulcin 2025). But once incorporated into food systems, stability becomes increasingly dependent on extrinsic conditions. Exposure to oxygen, light, temperature, pH, water activity, and interactions with other molecules can lead to degradation pathways that determine how much of the original antioxidant remains available during processing and storage (Tarlak 2023). It should be noted that thermal processing of protein‐rich plant matrices (legumes, cereals, oilseeds) can also generate bioactive peptides with radical‐scavenging and metal‐chelating properties through heat‐induced protein unfolding and hydrolysis, while simultaneously producing Maillard‐derived melanoidins with documented antioxidant activity (Rebollo‐Hernanz et al. 2023). However, as the present review focuses on phytochemical antioxidants, whose structural diversity and matrix interactions are its central theme, peptide‐derived and PS‐derived antioxidant activities, which are reviewed elsewhere, are not discussed in detail in the processing subsections.

The impact of these factors differs across the production chain. Processing steps impose rapid and intense changes in temperature, pressure, or electric‐field strength, which can either facilitate the release of matrix‐bound antioxidants from disrupted cell compartments or accelerate their degradation through thermal cleavage, oxidation, and isomerization, depending on the applied technology and the physical state of the matrix (Kasote et al. 2021).

During storage, the same physicochemical variables act more gradually, driving slower, cumulative losses governed by first‐ or higher order kinetics, with activation energies (*E_a_*) that are compound‐ and matrix‐specific. For instance, in blackberry extracts, the choline chloride:glycerol deep eutectic solvent (CHGLY) acted as a protective matrix that substantially slowed pigment degradation relative to water, reducing degradation rate constants by 3‐ to 18‐fold during dark storage and extending half‐life values to 341.44–1238.27 h, whereas the corresponding values in aqueous extracts were only 64.58–163.79 h. This matrix effect was also evident in anthocyanin retention after 28 days in darkness, when CHGLY preserved 67.97% of cyanidin‐3‐glucoside, 68.62% of cyanidin‐3‐rutinoside, 25.55% of pelargonidin‐3‐glucoside, and 67.18% of cyanidin chloride, whereas the same compounds in water declined to 0.07%, 2.82%, 1.10%, and 5.82%, respectively. Under light exposure, the same pattern persisted, with half‐life values of 283.59–589.49 h in CHGLY compared with 65.79–170.42 h in water, confirming that the surrounding medium strongly modulates the susceptibility of anthocyanins to cumulative storage losses. Thermodynamic analyses further reinforced this interpretation, as the lower *k* values and more favorable kinetic–thermodynamic parameters observed in CHGLY indicated greater resistance to heat‐induced breakdown than in aqueous solution. Mechanistically, this enhanced preservation has been attributed to the strong hydrogen‐bonding network established between anthocyanins and the deep eutectic solvent components, which stabilizes the flavylium structure and restricts degradation pathways during storage, light exposure, and heating (Zannou et al. 2025). Taken together, these results provide a clear example of matrix‐mediated stabilization: The retention of plant antioxidants during storage depends on compound class and on the ability of the surrounding matrix to limit molecular mobility, protect reactive sites, and attenuate environmentally induced degradation.

**FIGURE 3:**
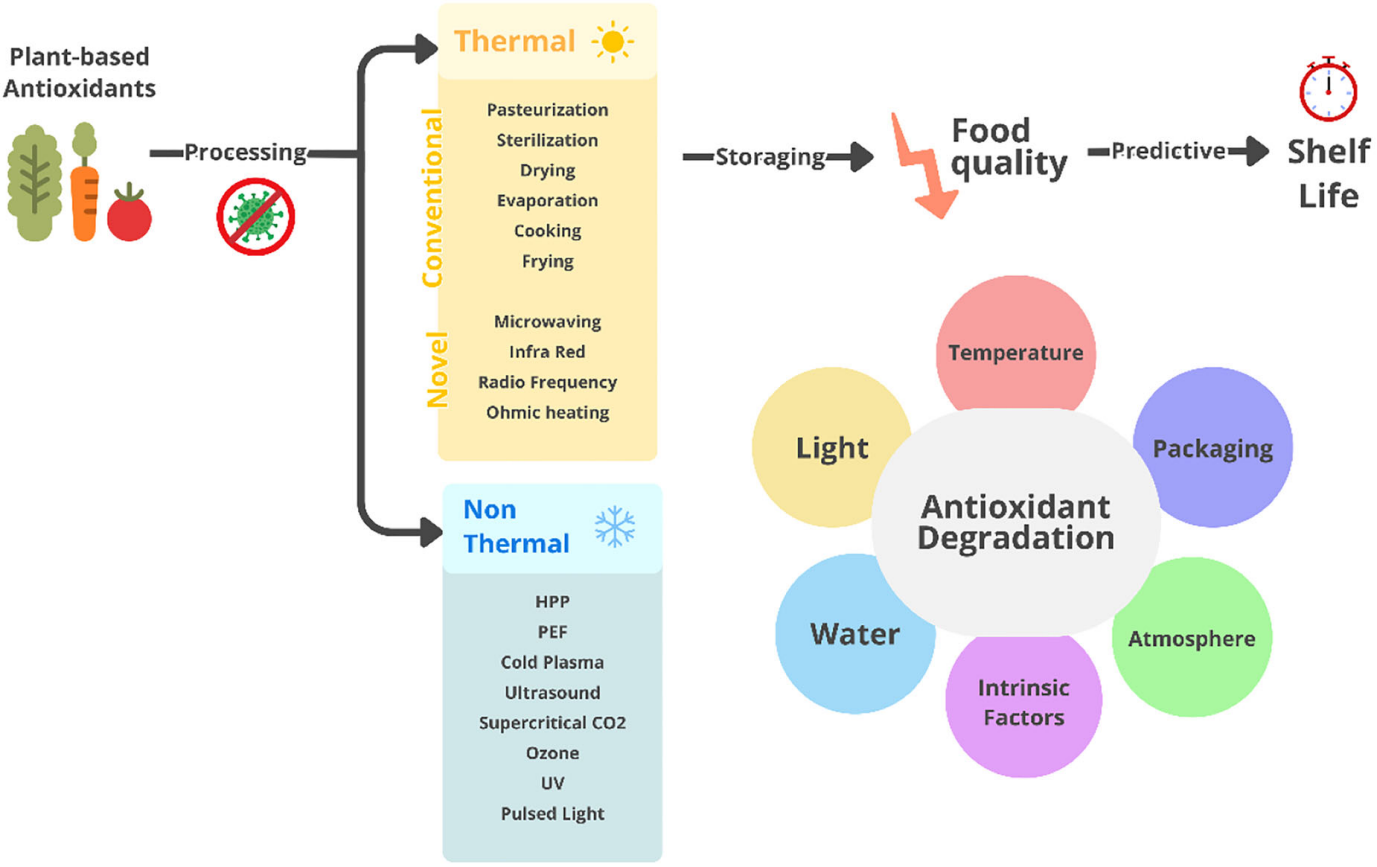
Overview of how thermal and nonthermal food processing, and storage affect the performance of plant‐based antioxidants in foods. HPP, high‐pressure processing; PEF, pulsed electric field.

The following subsections are therefore organized according to the dominant mechanisms through which processing affects antioxidant compounds. In each case, quantitative degradation data, kinetic parameters, and matrix‐dependent effects are prioritized over descriptive technology overviews (Figure 3). Throughout this section, it should be noted that total phenolic content (TPC) values derived from the Folin–Ciocalteu assay reflect total reducing capacity rather than phenolic content exclusively, and may be influenced by non‐phenolic reductants such as ascorbic acid and Maillard reaction products generated during thermal processing (Nastasi 2025). Where available, HPLC‐based quantification is indicated.

### 4.1 Thermal Degradation Pathways and Their Effects on Antioxidant Compounds

Thermal processing remains the most widely applied preservation approach in the food industry, based on the controlled application of heat to inactivate spoilage‐associated microorganisms and enzymes (dos Santos Rocha et al. 2022). In plant‐based systems, the primary mechanisms through which heat degrades antioxidants are four: (i) thermolytic cleavage of covalent bonds in heat‐sensitive chromophores, particularly the pyrylium ring of anthocyanins and the conjugated polyene chain of carotenoids (Y. Zhang et al. 2020); (ii) oxidative degradation catalyzed by transition metals and accelerated by increased molecular mobility at elevated temperatures (Mertens et al. 2022); (iii) nonenzymatic Maillard‐type and condensation reactions that consume phenolic hydroxyl groups (Bork et al. 2024); and (iv) hydrolytic deglycosylation of flavonoid glycosides, producing more reactive aglycones with shorter half‐lives (Tsuruoka et al. 2025).

Simultaneously, heat can increase the net extractable antioxidant pool by inactivating PP oxidase (PPO), disrupting cell‐wall PS matrices, and releasing matrix‐bound phenolics. This phenomenon explains the paradoxical increase in TPC observed, for instance, in steamed broccoli (TPC increased by approximately 29% vs. fresh controls in dried‐basis measurements) despite thermolytic losses of individual compounds (Kim et al. 2021).

The balance between these opposing mechanisms is highly matrix‐dependent: In liquid systems (juices, broths), soluble phenolics are directly exposed to the thermal environment with limited diffusional barriers (Pasquet et al. 2024), whereas in solid or semi‐solid matrices (fruits, vegetables, pastes), tissue structure, water activity, and cell‐wall integrity create microenvironmental heterogeneity that modulates degradation rates (Y. Wu et al. 2024). The following subsections examine each conventional thermal technique from this mechanistic perspective, integrating quantitative data on antioxidant retention and kinetic parameters.

#### 4.1.1 Pasteurization

Pasteurization applies moderate heat (typically 60–95°C) for defined periods to liquid and semi‐liquid plant‐based products, with high‐temperature short‐time (HTST) regimes (e.g., 95°C/8 s) generally associated with superior retention of phenolic compounds and pigments compared to medium‐temperature short‐time (MTST) protocols (e.g., 76°C/19 s), owing to the shorter cumulative thermal exposure (Utpott et al. 2025).

Quantitatively, the impact on antioxidant compounds is well documented: In strawberry juice systems, thermal pasteurization reduced anthocyanin content, with degradation following first‐order kinetics whose rate constants were strongly dependent on the juice system composition and preservation technology applied (Stübler et al. 2021). In orange juice, considering the thermal treatment (80°C/30 min) in comparison to PEF (120 kJ/L–24 kV/cm), a modest but consistent advantage of PEF was previously reported (Ozkan et al. 2025) for several bioactive markers, with total phenolics, total flavonoids, total anthocyanins, and CUPRAC antioxidant capacity being approximately 1.04‐, 1.11‐, 1.06‐, and 1.03‐fold higher, respectively. In contrast, the same study (Ozkan et al. 2025) assessed that HPP (600 MPa/3 min) was largely comparable to thermal treatment for total phenolics and flavonoids but showed lower anthocyanin retention and antioxidant capacity, with thermal treatment being about 1.06‐fold higher for total anthocyanins and 1.07‐fold higher for CUPRAC. Thermal treatment, however, outperformed both nonthermal treatments for vitamin C retention, remaining approximately 1.11‐fold higher than HPP and 1.29‐fold higher than PEF. Overall, these data indicate that PEF was the most effective treatment for preserving phenolic‐related antioxidant quality, whereas thermal treatment provided the greatest advantage for vitamin C stability (Ozkan et al. 2025). The predominant degradation mechanism during pasteurization of phenolic‐rich juices involves oxidative transformation accelerated by dissolved oxygen, rather than direct thermolysis, particularly for ascorbic acid and anthocyanins; this explains why processing under inert atmospheres (N_2_ headspace) has been shown to significantly improve antioxidant retention in pasteurized pineapple juice during 119‐day storage at 7°C (Kopuncová et al. 2025).

From a kinetic standpoint, anthocyanin degradation during pasteurization is typically described by first‐order kinetics, although thermal stability remains matrix dependent. In a maqui–citrus beverage, first‐order models captured anthocyanin losses after thermal pasteurization (85°C/15 s), whereas strawberry juice subjected to 60–80°C thermal sterilization also followed first‐order behavior, with copigments lowering degradation rates and extending half‐life during processing (Hernández‐Prieto et al. 2024; Sun et al. 2025), and 43 kJ/mol for açaí pulp (Marangoni Júnior et al. 2020). In the case of loengo (*Anisophyllea boehmii*) nectar, F. J. da Costa et al. (2026) reported that anthocyanins exhibited high thermal stability under conventional pasteurization conditions (≤85°C), with measurable degradation occurring only under severe heating (100°C), a behavior consistent with matrices characterized by moderate activation energies and strong buffering effects. These differences in thermal sensitivity reflect the protective role of co‐extracted matrix components, such as organic acids, suspended solids, and copigmentation partners, which limit heat‐induced degradation through anthocyanin–copigment and anthocyanin–matrix interactions (Y. Zhu et al. 2020).

These findings indicate that the dominant degradation pathway and the magnitude of losses are compound‐specific and strongly influenced by the matrix physical state: Liquid systems with high water activity and dissolved oxygen are more susceptible than viscous pulps or concentrates where molecular mobility is constrained. Nevertheless, pasteurization, by definition a sub‐100°C treatment, cannot inactivate bacterial endospores or highly heat‐resistant enzymes, which limits its application to refrigerated, acidic, or short‐shelf‐life products and can necessitate the use of sterilization‐grade thermal treatments to fill this aim (Estrella‐Osuna et al. 2024).

#### 4.1.2 Sterilization

Sterilization subjects foods to temperatures exceeding 100°C, conventionally in retorts/autoclaves (115–121°C for 15–60 min) or via UHT processing (130–150°C for 2–10 s), imposing a substantially greater thermal load than pasteurization (Y. Wu et al. 2024).

The impact on antioxidant compounds is correspondingly more severe under sterilization conditions, driven by irreversible thermolytic cleavage and oxidative degradation whose extent depends on both the time–temperature profile and the structural identity of the target molecule. In freshly squeezed lettuce juice, UHT sterilization (115°C, 5 s) caused marked reductions in chlorophyll, β‐carotene, and multiple vitamins, while shifting the color from bright green to light brown due to extensive chromophore degradation (J. Zhang, Cheng, et al. 2024). The magnitude of these losses, however, is strongly compound‐dependent in black carrot juice. TPC declined by only 0.80%–1.48% across thermal sterilization treatments, a resilience attributed to the high thermostability of acylated anthocyanins and intramolecular co‐pigmentation of phenolic acids, whereas UHT processing reduced total flavonoid and anthocyanin contents by 14.16% and 8.55%, respectively (Bao et al. 2023). Similar compound‐selective vulnerability was confirmed in not‐from‐concentrate *Actinidia arguta* juice, where thermal sterilization reduced total phenols by 8.53%–15.98% while significantly degrading ascorbic acid and chlorophyll (Chen et al. 2025).

The kinetic interpretation is straightforward: At sterilization temperatures (>100°C), degradation rate constants increase exponentially according to the Arrhenius relationship, and the extremely short holding times in UHT (2–5 s) limit the integral thermal exposure despite the elevated temperature, reducing total antioxidant loss (Y. Wu et al. 2024). Nevertheless, both approaches alter the redox environment of the matrix: Retort sterilization in sealed containers depletes dissolved oxygen, potentially limiting oxidative degradation during subsequent storage, whereas UHT processing followed by aseptic filling may introduce headspace oxygen that accelerates post‐processing losses if packaging barrier properties are inadequate (Polak et al. 2024).

Because industrial sterilization profiles are inherently non‐isothermal, involving continuous come‐up, holding, and cooling phases, the isothermal rate constants alone underestimate cumulative antioxidant losses during transient heating. Non‐isothermal Arrhenius‐based models, including the Weibullian‐log‐logistic approach, integrate instantaneous degradation rates over the entire thermal history, yielding more accurate predictions of anthocyanin and ascorbic acid retention during pasteurization and sterilization processing (Amodio et al. 2015). Such models show that the full thermal profile, rather than the holding‐phase conditions alone, must be considered. Detailed parameterization of non‐isothermal degradation models, including *E_a_* estimation, model comparison, and the integration of packaging OTR and dynamic headspace composition into degradation kinetics, is discussed in Section 5.

#### 4.1.3 Drying

Thermal drying encompasses a range of methods, from convective tray and tunnel systems to spray drying and freeze drying, that remove water from plant‐based matrices to reduce water activity and retard microbial, enzymatic, and oxidative deterioration (ElGamal et al. 2023). Although widely applied to fruits, vegetables, herbs, and cereals (Asrate and Ali 2025), the primary concern for antioxidant preservation is the cumulative time–temperature integral imposed during moisture removal, which governs the extent of thermolytic, oxidative, and Maillard‐mediated phenolic losses (Vidinamo et al. 2022).

From the standpoint of antioxidant stability, the critical determinant during drying is the time–temperature integral that phenolic compounds experience during moisture removal. Spray drying, commonly applied to fruit juice concentrates, employs inlet temperatures of 150–200°C but outlet temperatures of only 60–90°C, with total thermal exposure of seconds; as a result, PP retention can exceed 80%–90% when maltodextrin or gum arabic serves as wall materials (Mutavski et al. 2025). By contrast, conventional convective drying of fruit matrices (60–80°C for 6–24 h) may degrade 25%–50% of anthocyanins and 30%–60% of ascorbic acid, following first‐order kinetics with rate constants that increase 2‐ to 4‐fold per 20°C increment (Grández‐Yoplac et al. 2021).

In solid matrices, the drying front creates a heterogeneous microenvironment: Surface layers experience higher temperatures and lower water activity, favoring Maillard‐type condensation reactions that consume phenolic hydroxyls, whereas interior zones retain higher moisture and may undergo enzymatic browning until PPO is thermally inactivated (ElGamal et al. 2023). Freeze drying, by contrast, minimizes thermal degradation through sublimation at low temperatures (−40°C to −20°C), achieving PP retentions of >95% in berry matrices, but at substantially higher energy costs and longer processing times (Asrate and Ali 2025). These observations highlight that drying method selection for antioxidant‐rich plant products should be guided by the thermal sensitivity of the target compounds and the product's physical state (liquid concentrate vs. solid piece vs. puree), rather than by microbial inactivation targets alone.

#### 4.1.4 Cooking

Domestic and industrial cooking methods, such as boiling, steaming, grilling, baking, roasting, and microwaving, differ fundamentally in their mode of heat transfer and, critically for antioxidant stability, in the extent of direct contact between the food matrix and a liquid or lipid cooking medium (Razzak et al. 2023). This distinction determines whether leaching, thermal degradation, or enzymatic browning predominates in phenolic losses, and the magnitude of these effects is strongly dependent on the plant tissue's physical structure and water content (Perucini‐Avendaño et al. 2026).

Quantitatively, the effect of domestic cooking on PP retention is highly variable and matrix‐ and microstructure‐dependent. Razzak et al. (2023) assessed six vegetables subjected to boiling, steaming, and microwave cooking, reporting that boiling induced the most pronounced losses of TPC (up to 70.3%) and total flavonoid content (TFC, up to 82.27%), primarily through leaching of hydrophilic phenolics into the cooking water. Steaming consistently preserved the highest phenolic fraction across nearly all matrices, likely because the absence of direct water immersion limits solubilization of water‐soluble phenolics while still permitting heat‐mediated inactivation of PP oxidases and release of cell‐wall‐bound PPs. Microwaving showed intermediate and matrix‐dependent effects on TPC but preserved over 90% of ascorbic acid (Razzak et al. 2023). These patterns were corroborated by Miškec et al. (2025), who demonstrated that steamed and air‐fried broccoli retained the highest TPC (0.72 ± 0.12 and 0.65 ± 0.15 mg GAE/g fw, respectively), whereas boiling caused substantial phenolic migration into the cooking water. Air‐frying also better preserved hydroxycinnamic acids, glucosinolates, and carotenoids than water‐based methods (Miškec et al. 2025). Critically, however, phenolic retention after cooking does not guarantee bioavailability upon ingestion. Abd Allah et al. (2025) showed that fresh broccoli TPC (610 mg GAE/100 g) decreased to 503–515 mg GAE/100 g in refrigerated boiled and steamed samples and more markedly to 368–393 mg GAE/100 g in their frozen counterparts. Subsequent in vitro gastrointestinal digestion caused additional HPLC‐quantified phenolic losses of 64.9% even in uncooked broccoli, rising to 88% in frozen boiled samples, indicating that post‐cooking compositional analysis alone substantially overestimates actual phenolic intake (Abd Allah et al. 2025).

Mechanistically, the competing processes during cooking are (i) cell‐wall disruption and PPO inactivation, which increase extractable phenolics; (ii) thermal and oxidative degradation, which destroy labile compounds; and (iii) leaching into cooking water or oil, which transfers compounds out of the food matrix. The net outcome depends on the cooking method, the physical structure of the plant tissue, and the polarity of the individual antioxidant compounds.

#### 4.1.5 Frying

Frying is a thermal process in which foods are immersed in heated oil, typically at 150–190°C, producing rapid surface dehydration, crust formation, and progressive interior heating by conduction (Valle et al. 2024). Although valued for high thermal efficiency and short processing times, frying simultaneously creates a strongly pro‐oxidant environment: Absorbed oil and its degradation products formed through repeated heating and oxygen exposure favor lipid oxidation, polymerization, and Maillard‐type condensation reactions that directly consume heat‐sensitive phenolics and carotenoids, whereas oil‐derived peroxyl radicals propagate chain oxidation within the food matrix (Ujong et al. 2023). The extent of antioxidant degradation depends critically on the frying medium's intrinsic antioxidant profile and the physical state of the food (surface‐to‐volume ratio, moisture content), as oils with higher natural antioxidant loads show measurably slower degradation kinetics (Tian et al. 2025).

Regarding antioxidant‐related compounds, French‐fry processing caused pronounced losses of hydroxycinnamic acids, especially chlorogenic acid (5‐CQA), with pre‐drying and frying accounting for the greatest reductions. In the final product, only approximately 1%, 3%, and 5% of the initial 5‐CQA content remained in light‐yellow‐, red‐, and purple‐fleshed potatoes, respectively, demonstrating the substantial degradation of endogenous phenolics during high‐temperature processing (Tajner‐Czopek et al. 2023). In olive oil used for repeated frying (180°C, 5 cycles), α‐tocopherol content declined by approximately 40% after the first cycle and was virtually depleted after three cycles, whereas sesamol‐rich sesame oil retained ∼60% of its initial radical scavenging capacity (DPPH assay) over five cycles, demonstrating that the intrinsic antioxidant profile of the frying medium directly governs degradation kinetics (Tian et al. 2025).

Collectively, the kinetic evidence from conventional thermal processes reveals a consistent pattern: Antioxidant degradation in plant‐based matrices follows predominantly first‐order kinetics, with rate constants (*k*) increasing exponentially with temperature according to the Arrhenius equation. Activation energies reported for anthocyanins in fruit matrices range from approximately 43 kJ/mol (açaí pulp) (Marangoni Júnior et al. 2020) to values exceeding 80 kJ/mol in carotenoid‐rich systems (Akonor et al. 2023), indicating compound‐specific thermal sensitivity that must be incorporated into time–temperature optimization models. The superiority of HTST over MTLT protocols for phenolic retention (Utpott et al. 2025), and the reduced losses achievable with UHT versus retort sterilization (Y. Wu et al. 2024), are direct kinetic consequences of this exponential dependence. Non‐isothermal models that integrate instantaneous degradation rates over the entire come‐up/hold/cool‐down profile therefore provide more realistic predictions than isothermal rate constants alone (see Section 5 for detailed parameterization and model comparison).

### 4.2 Volumetric and Alternative Thermal Technologies: Mechanistic Advantages for Antioxidant Retention

The principal advantage of novel thermal technologies for antioxidant retention lies in their ability to achieve volumetric or targeted heating, thereby reducing the thermal gradient between the surface and the core and minimizing over‐processing of heat‐sensitive compounds. However, the extent of these advantages is matrix‐dependent: In highly heterogeneous semi‐solid systems (e.g., vegetable soups, sauces), processing can create localized zones where antioxidant degradation approaches that of conventional thermal processing (H. Zhang, Gao, et al. 2025).

In the following sections, we critically evaluate how the alternative thermal processing technologies, microwave, ohmic heating, RF, and infrared heating (IH) differ in their mechanisms and effectiveness for preserving antioxidants.

#### 4.2.1 Microwave

Microwave heating relies on the interaction of electromagnetic waves at 300 MHz–300 GHz with polar molecules in food, especially water, causing dipolar rotation and ionic conduction that generate heat directly within the matrix (Joardder and Karim 2025). This mechanism characterizes microwave processing as a volumetric heating method, allowing much faster energy transfer than conventional techniques based on conduction or convection. The principle is the same at domestic and industrial scales, but industrial systems offer greater operational control (Kamble et al. 2020).

With respect to antioxidant compounds, microwave heating exhibits a dual, matrix‐ and microstructure‐dependent behavior. In solid plant matrices, microwave treatment (700–900 W, 2–5 min) has been shown to maintain or enhance TPC relative to raw samples in several vegetables: Broccoli showed increased TPC after microwave cooking at 700 W for 2 min, whereas Chinese kale subjected to microwave boiling and steaming at 900 W significantly enhanced TPC from 1605 to 1635–2014 mg GAE/100 g dw and increased antioxidant activity assessed by DPPH and ABTS assays, likely because rapid volumetric heating inactivates PPO before enzymatic browning can proceed, whereas short exposure time limits thermolytic degradation (Chin et al. 2022; Kim et al. 2021). In a systematic comparison of cooking methods applied to multiple vegetables, microwaving generally enhanced or preserved TPC and radical scavenging capacity relative to raw controls, whereas boiling induced the greatest losses, confirming that the absence of a leaching medium is as important as temperature control for phenolic retention (Lisciani et al. 2025).

On the other hand, in liquid and semi‐liquid systems (juices, purees), where hot‐spot formation leads to localized overheating, microwave pasteurization may not consistently outperform conventional HTST protocols: In orange juice pasteurized by continuous‐flow microwave‐assisted heating at equivalent lethality (70–100°C), the model was able to predict pectin methylesterase inactivation and ascorbic acid retention, indicating that microwave pasteurization can be as efficient as conventional heating on enzyme inactivation with comparable or slightly improved impact on vitamin C, suggesting that the advantage of microwave processing for antioxidant retention in liquids is limited primarily to reduced come‐up and cool‐down times rather than to fundamentally different degradation pathways (Amaro et al. 2024).

These findings indicate that microwave heating offers clear advantages for antioxidant preservation in solid, low‐moisture, or particulate matrices where rapid PPO inactivation and minimal leaching dominate. In contrast, its benefits in homogeneous liquid systems depend critically on the elimination of hot spots through uniform field distribution.

#### 4.2.2 Infrared Heating

IH uses electromagnetic radiation in the 0.78–1000 µm range, transferring energy directly to the food surface via vibrational absorption. As a radiative process, IR promotes rapid heat transfer and a rapid rise in surface temperature, making it particularly suitable for operations that require a quick thermal response (Fakayode et al. 2025).

In industrial applications, IH is commonly used for accelerated drying, surface toasting, preheating, and processes involving partial moisture removal or color development, and it can be implemented in continuous systems or enclosed chambers. However, its penetration depth is limited and strongly dependent on wavelength and the optical properties of the food matrix, leading to pronounced temperature gradients between the surface and the interior (Sakare et al. 2020). For this reason, IR heating is often combined with other thermal techniques to improve heat uniformity and to avoid surface overheating, excessive browning, texture deterioration, or localized degradation of heat‐sensitive antioxidants (An et al. 2025).

Regarding antioxidant stability, the shallow penetration depth of IR radiation (<5 mm in most plant matrices) creates steep thermal gradients that preferentially degrade surface‐layer phenolics while leaving interior antioxidants relatively intact. In apple slices subjected to infrared‐assisted hot‐air drying (50–70°C, air velocity 1–3 m/s), total PP retention ranged from approximately 69% to 80% of the fresh‐weight value, with higher drying temperatures yielding paradoxically better retention due to reduced total drying time that limited the cumulative oxidation of phenolic and flavonoid compounds (Huang et al. 2022).

For carotenoid‐rich matrices, IR‐assisted drying has also demonstrated advantages over convective methods, as the rapid surface dehydration can form a low‐*a_w_* crust that slows oxygen diffusion into the interior, partially protecting carotenoids from oxidative degradation (An et al. 2025). However, published kinetic models specifically parameterizing antioxidant degradation under IR heating conditions remain scarce, and most available retention data derive from endpoint comparisons rather than time‐resolved kinetic analysis, limiting the ability to predict IR‐mediated losses across different product geometries and moisture profiles. On the other hand, when applied to thin, high‐surface‐area materials such as herb leaves or tea, IR heating at high intensity can rapidly surpass 100°C at the surface, causing localized pyrolysis of chlorophyll and thermolabile flavonoids; hence, combined IR–convective systems that balance surface temperature control with volumetric moisture removal are preferred for antioxidant‐rich matrices (An et al. 2025; Fakayode et al. 2025).

These results confirm that IR heating is most advantageous for solid, structured matrices where the surface‐concentration effect can be managed and least appropriate for liquid systems where its lack of volumetric penetration provides no advantage over conventional heating.

#### 4.2.3 Radiofrequency

RF heating employs low‐frequency electromagnetic fields, typically at 13.56, 27.12, or 40.68 MHz, to generate heat within food through ionic polarization and charge displacement. Due to their long wavelength, RF allows energy to penetrate deeply into the matrix, producing more uniform temperature distributions than conventional surface‐heating methods and generally fewer hot spots than microwave processing, particularly in thick or dense products (Costa and Marra 2024).

At the industrial scale, RF technology is applied in operations such as accelerated drying, preheating, rapid thawing, and thermal treatment of bulk plant‐based materials, using generators connected to parallel‐plate electrodes through which the product is continuously held or conveyed in free‐running oscillator (FRO) systems (Gao et al. 2023). Its main advantages lie in reduced processing times and improved heating uniformity, which lower surface thermal gradients and the risk of localized overheating. However, RF efficiency is strongly dependent on matrix properties such as moisture content, electrical conductivity, and dielectric behavior, and uneven heating in zones with higher local conductivity requires careful process control (H. Zhang, Gao, et al. 2025).

From the perspective of antioxidant preservation, RF heating offers its principal advantage in thick, dense, or bulk plant matrices where conventional heating requires prolonged exposure to achieve core temperatures sufficient for microbial inactivation, precisely the conditions that maximize cumulative antioxidant degradation. In RF‐assisted post‐harvest treatment of in‐shell walnuts (27.12 MHz, two‐stage strategy with transition temperature 55°C, holding time 5 min), product quality indicators including peroxide value (0.183 meq/kg), free fatty acid content (0.128%), and color parameters remained within industry quality standards and were not significantly affected by the treatments, whereas conventional hot‐air treatment to the same target temperature required substantially longer processing durations that increase cumulative thermal exposure and the risk of quality deterioration (Mao et al. 2021; Mao and Wang 2021). Similarly, in RF thawing of frozen plant‐based products, the rapid and uniform temperature rise characteristic of RF heating minimizes prolonged exposure to moderate temperatures (20–40°C), thereby allowing enzymatic degradation to proceed during conventional slow thawing processes (Gao et al. 2023).

As with IH, published kinetic models explicitly parameterizing antioxidant degradation under RF heating conditions are limited; the available evidence is based primarily on endpoint retention comparisons rather than time‐resolved kinetic analysis, and further work is needed to derive compound‐ and matrix‐specific rate constants for RF‐processed plant foods. Nevertheless, in high‐conductivity liquid matrices such as brines or ionic solutions, RF energy preferential coupling to the ionic phase can create localized hot spots at solid–liquid interfaces, potentially accelerating phenolic oxidation in those microenvironments. For this reason, RF heating performance for antioxidant retention is strongly matrix‐dependent: It excels in low‐moisture solids and particulate systems but requires careful process control in heterogeneous semi‐solid and high‐salt systems (H. Zhang, Gao, et al. 2025).

#### 4.2.4 Ohmic Heating

The ohmic heating technique is based on the passage of alternating electric current directly through the food matrix, where heat is generated internally due to its intrinsic resistance (the Joule effect) (Srivastava and Sit 2025). Because energy conversion occurs within the product itself, heating is rapid, volumetric, and highly uniform, without reliance on external heat transfer gradients. Industrial applications typically involve continuous or semi‐continuous systems equipped with electrodes and controlled voltage and frequency settings, allowing precise adjustment of heating intensity and thermal profiles (dos Santos et al. 2024). This makes ohmic heating particularly suitable for liquid and semi‐liquid plant‐based matrices. The main advantages of this method include reduced processing times, improved temperature homogeneity, and enhanced preservation of heat‐sensitive compounds resulting from minimized thermal exposure (Srivastava and Sit 2025).

However, effective operation depends on the product's adequate electrical conductivity and on heterogeneities, such as large particles, which can compromise heating uniformity (Sain et al. 2024). In terms of antioxidant outcomes, ohmic heating can yield improved retention of heat‐labile compounds compared to conventional thermal processing at equivalent lethality, primarily because its shorter come‐up time reduces cumulative thermal exposure during the non‐isothermal phase. In guava pulp subjected to ohmic heating (21.2 V/m, 60 Hz, 60–80°C), carotenoid content was significantly better preserved at the two lowest temperatures tested compared with conventional water‐bath heating. At the same time, ascorbic acid degradation followed first‐order kinetics under both treatments, and the ohmic system achieved an average thermal efficiency of 40.93% compared with only 2.62% for conventional heating (Giuliangeli et al. 2023). In strawberry nectar processed by pilot‐scale ohmic heating, treatments designed to achieve 5‐log microbial reduction preserved vitamin C with retention levels consistent with kinetic predictions based on the reduced come‐up time characteristic of ohmic heating compared with conventional pasteurization (Pavon‐Vargas et al. 2025).

These kinetic advantages are most pronounced in liquid and semi‐liquid matrices (juices, pulps, sauces) where electrical conductivity is sufficient for efficient Joule heating; in solid particulate systems with large non‐conductive inclusions, heterogeneous heating can occur at particle boundaries, partially negating the kinetic advantage (Srivastava and Sit 2025). Accordingly, ohmic heating is among the thermal technologies with the highest reported antioxidant retention in conductive fluid systems, provided electrical conductivity is sufficient for uniform Joule heating, but its application to structurally heterogeneous foods requires careful electrode design and process validation to ensure uniform treatment (Sain et al. 2024; Srivastava and Sit 2025).

### 4.3 Nonthermal Technologies: Cell Disruption, Oxidative Stress, and Antioxidant Stability Trade‐Offs

Nonthermal technologies have emerged to apply alternative forms of energy or minimally processed foods that maintain quality and nutritional value while ensuring microbiological safety. Unlike heat‐based methods, these approaches use alternative forms of energy such as pressure, electric fields, ionized plasmas, acoustic waves, or supercritical fluids that can modify cellular structures, reduce microbial load, or promote physicochemical stability without causing extensive damage to the plant matrix (White et al. 2025).

The net antioxidant outcome of nonthermal processing, therefore, depends on the balance between compound liberation (positive) and increased oxidative susceptibility (negative). This balance shifts with the matrix's physical state, the intensity and duration of treatment, and the specific chemistry of the target antioxidant class. The following subsections examine each technology through this mechanistic lens, with emphasis on quantitative evidence from plant‐based food systems.

#### 4.3.1 High‐Pressure Processing

HPP subjects packaged foods to 200–700 MPa for 3–10 min, disrupting cellular membranes and supramolecular structures while leaving covalent bonds largely intact (Aganovic et al. 2021). Because the temperature rise during treatment is minimal, heat‐labile chromophores and phenolic structures are not subjected to thermolytic cleavage, unlike in conventional thermal processing, making HPP one of the most effective technologies for preserving anthocyanin, carotenoid, and PP profiles in fruit‐ and vegetable‐based matrices (Y. H. Hu et al. 2020).

The principal mechanisms by which HPP modulates antioxidant stability are two: (i) suppression of endogenous PP oxidase (PPO) and peroxidase activity through pressure‐induced protein unfolding, which prevents enzymatic browning; and (ii) disruption of cell‐wall and vacuolar compartments, releasing matrix‐bound phenolics into the bulk phase and increasing measurable antioxidant content, an effect that simultaneously enhances oxygen diffusion and may therefore promote posttreatment oxidative losses in solid tissues (Stübler et al. 2021).

In plant‐based foods, this translates into improved retention of heat‐sensitive antioxidants, including phenolic compounds, anthocyanins, and carotenoids, and a reduced formation of secondary oxidation products. Despite these advantages, HPP faces significant limitations and variable effectiveness depending on intrinsic factors such as pH, tissue structure, and cellular composition. In addition, pressure‐induced disruption of cellular compartments may increase oxygen availability within the matrix, thereby promoting localized oxidative losses of susceptible antioxidant compounds during storage (Pérez‐Lamela et al. 2021).

Recent HPP studies indicate that antioxidant behavior in plant‐based foods is better interpreted kinetically as the combination of initial retention immediately after pressurization and the subsequent apparent storage‐decay trajectory, rather than by assuming a single universal degradation order across matrices (Ahmad et al. 2024; Ambreen et al. 2023). In orange juice treated at 400–600 MPa for 3–9 min, HPP largely preserved ascorbic acid and maintained physicochemical stability during 60 days of storage, whereas thermal processing caused a marked reduction in ascorbic acid and poorer carotenoid preservation, consistent with a lower apparent storage‐dependent loss rate under HPP (Ambreen et al. 2023). However, HPP‐induced cellular disruption in solid plant tissues (e.g., fresh‐cut strawberries at 500 MPa) has been shown to increase oxygen diffusion into the tissue matrix by compromising cell membrane integrity, leading to paradoxical increases in enzymatic and nonenzymatic oxidation of anthocyanins during posttreatment storage, an effect not observed in liquid systems where oxygen is more uniformly distributed (Stübler et al. 2021). This phenomenon illustrates that the physical state of the food matrix (liquid vs. solid) critically determines whether HPP results in a net positive or negative effect on antioxidant retention.

#### 4.3.2 Pulsed Electric Field

PEF processing applies short, high‐voltage pulses (10–90 kV/cm, microsecond to millisecond duration) to liquid or semi‐solid foods, achieving microbial inactivation through electroporation. It means irreversible permeabilization of cell membranes without thermolytic cleavage, oxidative chain propagation, or Maillard‐mediated condensation reactions that degrade phenolic compounds during conventional heat processing (Arshad et al. 2021). Because temperature rise is minimal and covalent bonds remain intact, PEF preserves the chemical integrity of heat‐sensitive antioxidants, particularly anthocyanins, hydroxycinnamic acids, and ascorbic acid, while simultaneously enhancing their measurable content through the release of phenolics previously compartmentalized within intact cell vacuoles and cell‐wall matrices (Leong et al. 2020).

Recent evidence indicates that the antioxidant outcome of PEF processing in plant‐based matrices is better interpreted as a matrix‐ and dose‐dependent balance between enhanced mass transfer and the risk of electrically induced oxidative reactions, rather than as a single universal field‐strength threshold (Carpentieri et al. 2023; Červinková et al. 2024; Faria and Silva 2026; Jara‐Quijada et al. 2024). Under mild‐to‐moderate treatment conditions, electroporation can increase the accessibility of intracellular phenolics and improve antioxidant performance without detectable degradation of major compounds (Carpentieri et al. 2023; Jara‐Quijada et al. 2024). For example, in red grape pomace, optimization of PEF at 4.6 kV/cm and 20 kJ/kg increased TPC by 15%, flavonoid content by 60%, total anthocyanins by 23%, tannins by 42%, and FRAP values by 31% relative to the untreated control, whereas HPLC‐PDA analysis detected no degradation of the major phenolics (Carpentieri et al. 2023). Likewise, in green tea, PEF‐assisted extraction at 5.88 kV/cm, 200 Hz, and 1000 µs increased extraction yield by 50% compared with maceration and improved DPPH radical inhibition by approximately 40%, with extraction kinetics adequately described by Peleg's model (*R* ^2^ = 0.978) (Jara‐Quijada et al. 2024).

However, recent work also shows that greater membrane permeabilization does not necessarily translate into proportionally higher antioxidant recovery: In fresh pomegranate peel, PEF pretreatment produced a cell disintegration index above 0.9 but resulted in only marginal improvements in phenolic recovery, indicating that in solute‐rich matrices the dominant limitation may be solvent‐driven diffusion rather than structural disruption (Faria and Silva 2026). In parallel, direct mechanistic evidence has demonstrated that PEF can generate H_2_O_2_ and superoxide via interfacial charge‐transfer reactions at the anode, providing a plausible explanation for oxidative losses when electrical input and electrode effects become excessive (Červinková et al. 2024). Collectively, these studies support the use of PEF as a kinetics‐guided formulation and processing tool for antioxidant‐rich plant systems, provided that the electric field strength, specific energy, pulse regime, conductivity, and matrix architecture are jointly optimized for each food material.

#### 4.3.3 Cold Plasma

Cold plasma is produced by ionizing gases, such as air, oxygen, nitrogen, argon, or helium, using high‐energy electric or electromagnetic fields. This process produces a complex mixture of reactive species, including electrons, ions, radicals, and excited molecules, that interact mainly with the food surface at low temperatures, thereby avoiding thermal damage (Harikrishna et al. 2023). Its antimicrobial and enzymatic effects are primarily driven by ROS and reactive nitrogen species (ROS and RNS), which disrupt cellular structures and metabolism. Due to its superficial mode of action, it allows relatively precise control over treatment depth while preserving the overall structural integrity of plant tissues (Farooq et al. 2023).

The impact of cold plasma on antioxidant compounds follows a dose‐dependent threshold pattern: At low intensity and short exposure, the absence of prolonged heating limits thermal degradation, and plasma‐induced membrane disruption releases cell‐wall‐bound phenolics; beyond this threshold, accumulated ROS and RNS directly oxidize susceptible antioxidants, particularly anthocyanins and carotenoids (Zargarchi et al. 2024).

Quantitatively, the impact of cold plasma on antioxidant compounds remains highly dependent on treatment intensity, gas composition, and matrix physical state. In a solid fruit matrix, low‐pressure cold plasma pretreatment of “Heidi” mango before hot‐air drying showed a dose‐dependent response: A 5 min treatment increased total flavanols and produced higher total phenolics than the untreated control, whereas a 10 min treatment resulted in the lowest antioxidant activity, indicating that mild exposure may preserve or enhance phytochemicals, whereas longer exposure becomes detrimental (Yanclo et al. 2024). By contrast, in liquid systems, the outcome appears especially sensitive to plasma gas composition. In cloudy apple and cantaloupe juices treated for 90 s, atmospheric cold plasma did not significantly alter overall physicochemical characteristics, but the antioxidant activity of cantaloupe juice decreased markedly when simulated air (80% N_2_/20% O_2_) was used, whereas a lower oxygen gas mixture (90% N_2_/10% O_2_) better preserved bioactive compounds (Ozen et al. 2024). A similarly optimization‐dependent pattern was also observed in buckwheat, where dielectric‐barrier‐discharge cold plasma increased total phenolics, total flavonoids, rutin, and antioxidant activity under selected voltage–time combinations in both flour and grain (Amiri et al. 2025).

These contrasting findings illustrate a critical threshold effect: At low intensity and short exposure (<120 s), plasma‐generated ROS activate endogenous stress–response pathways and release bound phenolics, effects particularly favorable in solid and semi‐solid matrices with intact cellular machinery. Beyond this threshold, accumulated ROS directly degrades susceptible antioxidants, especially in liquid matrices where phenolic compounds are freely dissolved and maximally exposed to reactive species. PPO and peroxidase activity reductions have been reported under atmospheric DBD plasma treatment, confirming that enzymatic browning suppression is an additional mechanism by which cold plasma indirectly preserves phenolic content in fresh‐cut products (Zargarchi et al. 2024).

#### 4.3.4 Ultrasound

Ultrasound processing employs high‐frequency sound waves, typically 20–100 kHz, transmitted through probes or ultrasonic baths into liquid or semi‐solid food matrices. The propagation of acoustic energy induces cavitation, characterized by the formation, growth, and collapse of microbubbles that generate localized microjets, shear forces, and transient high‐pressure zones capable of disrupting cellular structures without prolonged heating (Chavan et al. 2022). These physical–mechanical effects enhance mass transfer and promote cell wall rupture, facilitating the release of phenolic compounds, flavonoids, and pigments previously bound within the plant matrix while largely preserving sensory attributes due to the minimal thermal load (Meena et al. 2024).

However, the use of high amplitudes, extended treatment times, or insufficient temperature control may lead to the formation of cavitation‐derived reactive species, favoring oxidative reactions and the degradation of sensitive compounds, particularly anthocyanins and carotenoids. Consequently, the impact of ultrasound on antioxidant stability is strongly dependent on the balance between acoustic intensity, exposure time, temperature evolution, and matrix composition (Salehi 2020).

Primary studies confirm the extractive and degradative potential of ultrasound, with matrix‐dependent effects. In blackcurrant juice, thermosonication (480 W, 40 kHz, 50°C for 30 min) increased TPC by 12.6%, total flavonoid content by 20.9%, and total anthocyanin content by 40.4% relative to the untreated control, whereas ascorbic acid loss was significantly lower than that observed after conventional pasteurization (90°C, 1 min); the enhancement was attributed to cavitation‐induced shear forces disrupting cell walls and converting bound phenolics to free forms through hydroxylation of the aromatic ring, thereby increasing net extractable PP concentration (Qiu et al. 2024).

Similarly, in garden cress (*Lepidium sativum*) juice processed under optimized ultrasound conditions (12 min, 80% amplitude), TPC (78.44 mg GAE/mL), ferric reducing antioxidant power (59.80 mg TE/mL), and chlorophyll and β‐carotene concentrations were significantly higher than those in both untreated and pasteurized samples (*p* < 0.05); moreover, simulated gastrointestinal digestion revealed an in vitro TPC recovery rate of 34.96%, indicating that cavitation‐assisted release of phenolics also enhanced their bioaccessibility relative to thermally treated counterparts (Levent et al. 2025). In Amazonian Cubiu (*Solanum sessiliflorum*) and Abricó (*Mammea americana*) juices, moderate ultrasound intensities promoted carotenoid release from ruptured chromoplasts and improved radical‐scavenging capacity (DPPH, ABTS^+^, FRAP); however, β‐carotene bioaccessibility after simulated digestion was strongly matrix‐dependent, declining markedly in Cubiu juice under acidic oxidative conditions while remaining more stable in the lipophilic matrix of Abricó juice, underscoring that the net bioaccessibility benefit of sonication depends on matrix composition and the physicochemical environment during digestion (Macalia et al. 2026).

By contrast, in blackberry juice subjected to thermosonication (20 kHz, central composite design varying amplitude 60%–90%, temperature 64–86°C, and time 114–517 s), the amplitude factor exerted a significant effect on bioactive compound stability: Higher ultrasonic amplitude (90%) promoted degradation of total anthocyanins and total phenolic compounds, whereas the combination of lower amplitude (60%) with moderate temperature (86°C) provided superior retention of bioactive content and antioxidant activity relative to conventional thermal treatment (*p* < 0.05), consistent with a threshold above which cavitation‐generated hydroxyl radicals overwhelm the extractive benefit of cell disruption (C. N. da Silva et al. 2025). A parallel pattern was observed in blueberry juice treated with pulsed thermosonication (60% and 100% amplitude at 45°C and 55°C): Anthocyanin retention was highest at 100% amplitude combined with the lower temperature (45°C), reaching approximately 69%, whereas samples treated at 55°C exhibited substantially greater anthocyanin losses regardless of amplitude, confirming the dominant role of temperature in driving anthocyanin degradation during ultrasound‐assisted processing of clarified berry matrices (Panaro et al. 2024).

These observations collectively reinforce that ultrasound is most beneficial at moderate intensity in solid‐rich or pulp‐based matrices where cell‐wall disruption enhances phenolic and carotenoid release and bioaccessibility. At the same time, caution is warranted in clarified or low‐viscosity systems where dissolved bioactives, particularly anthocyanins, are directly exposed to cavitation‐generated reactive species and thermal synergy.

#### 4.3.5 Supercritical CO_2_

Supercritical CO_2_ (scCO_2_) processing exploits the hybrid solvent–gas properties of carbon dioxide above its critical point (31.1°C, 7.38 MPa) to achieve microbial inactivation in plant‐based foods under conditions that are inherently favorable for antioxidant preservation: The operating temperature rarely exceeds 40°C, far below the threshold for thermolytic cleavage of anthocyanin pyrylium rings, carotenoid polyene chains, or flavonoid glycosidic bonds, and the CO_2_‐saturated environment displaces dissolved oxygen, suppressing the oxidative degradation, enzymatic browning, and Maillard‐type condensation reactions that consume phenolic hydroxyl groups during thermal processing (Andrigo et al. 2026; W. Wang et al. 2021).

The process, conducted in pressurized batch or continuous systems through sequential pressurization, holding, and depressurization stages, additionally disrupts vacuolar membranes and weakens cell‐wall PS matrices through CO_2_ dissolution and carbonic acid formation, releasing matrix‐bound phenolics into the bulk phase. In pomegranate juice, optimized scCO_2_ pasteurization (12.7 MPa, 45°C, 40 min) maintained TPC and color stability comparable to the untreated juice over 28 days of refrigerated storage, outperforming conventional thermal pasteurization (90°C/1 min); however, antioxidant activity declined significantly during storage across all treatments, including scCO_2_, thermal, and HHP, indicating that TPC preservation does not necessarily guarantee functional antioxidant stability (Bertolini et al. 2020).

However, the antioxidant benefits of scCO_2_ processing are strongly dictated by matrix microstructure. In liquid and puree‐type systems where CO_2_ dissolves uniformly, the combination of low temperature, oxygen exclusion, and enhanced extractability delivers consistently high retention of heat‐labile compounds. In solid or highly viscous matrices, by contrast, CO_2_ penetration is limited by diffusional resistance, reducing both antimicrobial efficacy and the extractive advantages for antioxidant compounds. Furthermore, the depressurization step introduces a specific risk for acid‐sensitive chromophores: Rapid CO_2_ expansion generates transient localized pH drops (through carbonic acid formation) that can shift the anthocyanin equilibrium away from the stable flavylium cation form, potentially increasing pigment losses in acidic juice systems (W. Wang et al. 2021). The following quantitative evidence illustrates these matrix‐dependent considerations.

In juice matrices, quantitative outcomes confirm that scCO_2_ can improve microbiological stability while only partially suppressing oxidative enzymes. In sugarcane juice processed at 7.4–35.1 MPa, 33–67°C, for 20–70 min, reductions reached 0.1–3.9 log for aerobic mesophiles and 2.1–4.1 log for molds/yeasts, whereas PPO and POD decreased only by 3.5%–64.2% and 0.3%–41.4%, respectively, with Δ*E* values of 2.0–12.3 (Pimenta et al. 2024). Similarly, scCO_2_ drying of strawberry slices (40°C, 13.3 MPa) maintained total vitamin C content and preserved 95% of total anthocyanins (61.68 mg/100 g) compared with fresh samples, while achieving complete inactivation of foodborne pathogens (*Escherichia coli* O157:H7, *Salmonella*, *Listeria*) (Zambon et al. 2022). These data suggest that scCO_2_ performance is strongly matrix‐dependent: Unlike the earlier strawberry‐juice case, enzyme inactivation in a less acidic system such as sugarcane juice was incomplete, indicating that antioxidant preservation cannot be inferred from pressure–temperature conditions alone but must be interpreted together with residual PPO/POD activity and likely storage behavior.

#### 4.3.6 Ozone

Ozone (O_3_) treatment exploits the high oxidation potential of this triatomic molecule (*E*° ≈ +2.07 V) to inactivate microorganisms and suppress deteriorative enzymes through oxidative reactions with cellular membranes, proteins, and nucleic acids under nonthermal conditions, thereby avoiding the heat‐induced degradation typically associated with conventional thermal processing of thermolabile bioactives; however, treatment intensity must be optimized to prevent oxidative losses of sensitive phytochemicals such as phenolic compounds (Mayookha et al. 2023) and carotenoids (Tanwar et al. 2025).

Applied as gaseous or aqueous ozone at controlled concentrations and exposure times, via direct gasification, immersion in ozonized water, or circulation over packaged products, the technology presents a narrow processing window for antioxidant preservation: At low doses and short exposures, ozone can paradoxically enhance phenolic content by triggering endogenous stress‐response pathways (phenylpropanoid biosynthesis) and by suppressing enzymatic browning through PPO oxidation, while simultaneously reducing residual oxygen that would otherwise promote nonenzymatic oxidation. Beyond a dose‐dependent threshold, however, the same oxidative power that targets microbial membranes also attacks the electron‐rich aromatic rings of flavonoids, particularly at the C2–C3 double bond of flavonols and the C‐ring of anthocyanidins, converting them to hydroxylated or quinone derivatives with diminished radical‐avenging capacity (Botondi et al. 2023). The balance between these beneficial and deleterious effects is governed by ozone concentration, contact time, the physical state of the matrix (solid surface vs. dissolved in liquid), and the intrinsic antioxidant profile of the plant tissue, as demonstrated by the following quantitative studies.

Quantitative studies confirm a narrow processing window for ozone in terms of antioxidant preservation. In fresh‐cut red pitaya, gaseous ozone treatment (10 µL/L, 60 min) significantly increased the extractability of free and bound phenols by 16.7% and 4.9%, respectively, and enhanced DPPH scavenging capacity and FRAP values relative to untreated controls, attributed to ozone‐induced cell wall disruption and upregulation of phenolic metabolism via the phenylpropanoid pathway (C. Li et al. 2022, 2025). However, at prolonged exposure times or higher concentrations, these benefits were reversed; TPC declined, and vitamin C was significantly degraded through direct electrophilic attack of ozone on phenolic aromatic rings, as demonstrated across multiple tropical fruit matrices (Y. Wang, Niu, et al. 2023).

In fresh‐cut fruits stored under continuous low‐concentration ozone exposure at 4°C, ozone treatment effectively suppressed microbial growth and maintained higher TPC relative to untreated controls, which showed TPC declines attributable to enzymatic browning; PPO and POD activities were modulated by ozone treatment in a dose‐dependent manner (C. Li et al. 2022).

These findings indicate that ozone is most effective as a post‐harvest surface decontaminant, applied at low concentrations and for short durations, to solid, intact plant tissues, where the antioxidant stress response counterbalances direct oxidative losses. In liquid systems (juices, beverages), ozone dissolves rapidly and reacts non‐selectively with both target microorganisms and dissolved phenolics; thus, precise dosing and rapid quenching are essential to avoid net antioxidant losses (Dubey et al. 2022).

#### 4.3.7 UV Radiation

UV radiation, primarily UV‐C (200–280 nm), affects antioxidant compounds in plant‐based foods through two opposing photochemical mechanisms whose relative dominance is governed by dose, matrix opacity, and the metabolic status of the target tissue (Mahdavian 2024). At low doses (typically 1.0–4.5 kJ/m^2^), UV‐C photons penetrate only the surface layers (<1–2 mm) of solid and semi‐solid matrices, where they trigger a hormetic stress response, upregulating phenylalanine ammonia‐lyase (PAL), chalcone synthase, and other enzymes of the phenylpropanoid pathway that increase de novo biosynthesis of phenolics and anthocyanins in the underlying tissue while simultaneously providing surface microbial inactivation through photonic damage to nucleic acids (Abdipour et al. 2019). At higher doses (>10 kJ/m^2^) or in optically transparent liquid systems where UV photons reach all dissolved chromophores throughout the product volume, photodegradation dominates: Direct absorption by conjugated aromatic systems causes ring‐opening, hydroxylation, and decarboxylation reactions that progressively diminish radical scavenging capacity, following first‐order photodegradation kinetics whose rate constants are directly proportional to UV fluence and inversely related to the optical density of the matrix (Jafari et al. 2025).

This dual behavior makes the food matrix's physical state and optical properties the primary determinants of whether UV‐C treatment results in a net increase or decrease in antioxidant content. In solid, pigment‐rich matrices with intact cellular metabolism (fresh‐cut fruits, leafy vegetables, berries), the biostimulation response consistently outweighs surface photodegradation, producing measurable increases in total phenolics and anthocyanins. In transparent liquid systems (clarified juices, coconut water), by contrast, UV photons penetrate uniformly, eliminating the protective role of matrix opacity and rendering antioxidant degradation dose‐dependent throughout the product volume. The technology is delivered via low‐ or medium‐pressure mercury lamps, pulsed light systems, or UV‐LEDs installed in tubular or flat‐plate reactors, offering operational advantages such as low energy demand, the absence of chemical residues, and compatibility with UV‐transparent packaging (Delorme et al. 2020; Jafari et al. 2025). The following quantitative evidence illustrates these contrasting outcomes across different plant‐based matrices.

In fresh‐cut apple peel subjected to combined wounding stress and UV‐A radiation, PAL activity increased by up to 1201% and TPC increased by 108%–118% under optimal stress conditions (high‐intensity cutting, 15°C, 48 h), through UV‐induced upregulation of the phenylpropanoid biosynthetic pathway (Villamil‐Galindo et al. 2025). In nectarine fruit, UV‐C treatment at 3 kJ/m^2^ activated the phenylpropanoid metabolic pathway and anthocyanin biosynthesis, increasing total phenolics, flavonoids, anthocyanins, and lignin content while simultaneously reducing brown rot disease incidence (Abdipour et al. 2019). Furthermore, UV‐C irradiation at 5.3–11.4 kJ/m^2^ significantly delayed the onset of both native mycobiota and *Botrytis cinerea* infection in blueberry fruit during 20‐day storage at 8°C (Jaramillo Sánchez et al. 2021).

By contrast, in transparent liquid systems (apple juice, coconut water), UV‐C treatment at the doses required for microbial inactivation (>10 kJ/m^2^) degraded ascorbic acid by 15%–35% and reduced total phenolics by 5%–12%, as reported in comparative processing studies of fruit juices and beverages (Jafari et al. 2025). Quercetin, a representative flavonol, is particularly photolabile: UV radiation attacks the C2–C3 double bond, producing hydroxylated ring‐opening products with reduced radical scavenging capacity (Wijesekara and Xu 2024). The critical variable is thus matrix opacity: In solid and semi‐solid matrices, UV penetration is limited to the surface (<1–2 mm), confining photodegradation to a thin boundary layer while triggering beneficial stress responses in underlying tissue; in optically transparent liquids, photons reach all dissolved chromophores, and degradation becomes dose‐dependent throughout the product volume. This mechanistic distinction positions UV‐C as a complementary technology for surface decontamination and stress‐elicitation in fresh produce, but as a less favorable standalone option for transparent beverages unless coupled with rapid‐flow‐through thin‐film reactors that minimize per‐molecule UV exposure (Jafari et al. 2025).

### 4.4 Hurdle Strategies: Synergistic Approaches and Quantitative Evidence

Although individual thermal and nonthermal technologies achieve measurable antioxidant preservation, their performance is constrained by the compound‐specific, matrix‐dependent trade‐offs documented in the preceding sections. To address these limitations, the combined application of preservation techniques, commonly referred to as hurdle technology, has been systematically evaluated. This approach is based on the strategic use of multiple preservation factors at moderate intensities, rather than relying on a single treatment applied at high severity, to achieve reliable stabilization while minimizing quality losses (Bigi et al. 2023).

In combined systems, different technologies are applied sequentially or simultaneously so that each hurdle targets distinct mechanisms of microbial inactivation or quality deterioration, such as membrane disruption, enzyme inhibition, oxidative stress, or metabolic exhaustion (de Chiara et al. 2026). The effects may be additive or synergistic, allowing lower treatment intensities for each technology while achieving equal or superior preservation outcomes. In plant‐based foods, specific combinations, such as mild heat followed by UV‐C, or cold plasma coupled with modified atmosphere packaging, have achieved antioxidant retention rates higher than the corresponding single‐technology controls in juices, minimally processed fruits, and vegetable products (Jafari et al. 2025; S. Zhou et al. 2024). The following quantitative evidence from primary studies illustrates this potential.

In strawberry juice, equivalent nonthermal pasteurization by HPP (300 MPa, 1 min) and PEF (35 kV/cm, 27 µs), each achieving ≥5‐log *E. coli* inactivation, increased total anthocyanin content by 15% and 17%, respectively, relative to untreated juice, whereas conventional thermal pasteurization (72°C, 15 s) did not; moreover, HPP‐treated juice maintained microbial stability and the highest anthocyanin levels throughout 42 days of refrigerated storage (Yildiz et al. 2021). In blueberry juice, manothermosonication (560 W, 40°C, 350 MPa, 5 min) achieved a 5.85‐log reduction in *E. coli* O157:H7 while retaining 97.49% of initial anthocyanins and reducing PPO residual activity to 10.91%, compared with only 85.25% anthocyanin retention and 2.05% residual PPO under conventional heat treatment at 80°C (J. Zhu et al. 2017). Similarly, in *Aronia melanocarpa* juice, thermosonication (40 kHz, 240 W, 45°C, 15 min) increased total anthocyanin, total phenolic, and total flavonoid contents by 5.27%, 2.47%, and 10.91%, respectively, whereas ultra‐high temperature processing and irradiation decreased these values; high hydrostatic pressure (400–600 MPa, 5 min) also preserved anthocyanins and phenolics while ensuring microbial safety (Lv et al. 2024).

In fresh‐cut produce, in‐package atmospheric cold plasma treatment (100 kV, 5 min) applied to carrot discs reduced total aerobic mesophiles and yeasts/molds by approximately 2 log_10_ CFU/g with minimal changes in total carotenoid content, pH, color, and texture (Mahnot et al. 2020). However, the efficacy of cold plasma is critically dependent on exposure duration: In blueberries treated with dielectric barrier discharge at 25 kV, total anthocyanin content did not differ from untreated controls at 60 and 120 s but declined significantly at 180 s, a threshold confirmed by targeted metabolomic and transcriptomic analyses showing dose‐dependent modulation of anthocyanin biosynthetic genes (Xu et al. 2024).

However, hurdle strategies remain under‐standardized for industrial implementation. Their performance, as well as the previously described techniques, is strongly matrix‐dependent and requires careful selection, sequencing, and fine adjustment of processing parameters, as poorly optimized combinations may lead to overlapping disruptive mechanisms that intensify degradation rather than prevent it. This complexity, together with higher operational demands and limited industrial standardization, has contributed to a degree of skepticism regarding large‐scale adoption (Mondol et al. 2025).

Collectively, the compound‐specific degradation kinetics, activation energies, and matrix‐dependent retention data presented across Sections 4.1–4.3 provide the quantitative foundation for the second and third steps of the integrative framework proposed in this review: deriving process/packaging set points from fitted degradation models and coupling them with matrix‐specific thermal profiles to optimize antioxidant retention during industrial processing.

## 5 Storage Conditions, Shelf Life, and Kinetic Modeling

Shelf life is the time during which a food maintains acceptable quality, safety, and nutritional value under defined storage conditions and is limited by microbial growth, chemical/physical changes, and sensory deterioration (Urugo et al. 2024; Venkatesan and Muniyan 2024). Fruits and vegetables are especially perishable due to ongoing post‐harvest metabolism (respiration, ethylene, enzymatic reactions), which accelerates senescence and oxidative losses (Dou et al. 2023). Processed foods, though physiologically inert, still undergo oxidation, hydrolysis, and physical changes over time. Therefore, defining and consistently controlling key storage variables are central to preserving antioxidants and extending shelf life.

Despite widespread recognition of these variables, most published reviews on plant‐based antioxidants have treated storage factors descriptively, without integrating quantitative kinetic frameworks that link environmental parameters to measurable degradation rate constants and shelf‐life predictions. This section, therefore, addresses this gap by synthesizing compound‐ and matrix‐specific kinetic parameters (rate constants, activation energies, *Q*_10_ values) across antioxidant classes and by connecting degradation formalisms, from isothermal Arrhenius models to non‐isothermal and multicomponent approaches, with practical shelf‐life estimation workflows applicable to the food industry.

Below, we synthesize the principal environmental and manufacturing factors governing antioxidant losses during storage. We then critically examine how kinetic modeling, from classical Arrhenius‐based approaches to non‐isothermal and multicomponent formalisms, translates these factors into predictive tools for antioxidant‐rich food products.

### 5.1 Environmental and Manufacturing Factors: Temperature, Water Content, Light Exposure, Atmosphere (O_2_ and CO_2_), and Packaging

Among environmental and manufacturing factors, temperature is widely recognized as one of the most important factors affecting antioxidant stability during storage. Higher temperatures accelerate reaction rates, oxygen mobility, and antioxidant degradation, whereas lower storage temperatures slow these processes (Tarlak 2023; Venkatesan and Muniyan 2024).

The magnitude of temperature effects on antioxidant degradation in plant‐based food matrices has been quantified in primary storage studies. Y. Zhou et al. (2025) monitored phenolic compound stability in berry mueslis (strawberry, blueberry, and blackcurrant) under real‐time storage at 23°C and accelerated shelf‐life testing at 40°C, demonstrating temperature‐dependent degradation across 29 anthocyanins, 40 flavonols, 16 phenolic acids, and 2 flavan‐3‐ols; all identified anthocyanins decreased significantly, with 54%–66% of total anthocyanins lost within the first 56 days at 40°C, whereas acylated anthocyanins showed enhanced stability. Similarly, predictive kinetic models compiled for olive oil PPs under different storage temperatures yielded activation energies in the range of 62–107 kJ/mol, with secoiridoid phenolics following pseudo‐first‐order degradation kinetics and degradation rates increasing substantially above 25°C, illustrating how Arrhenius‐derived parameters enable shelf‐life extrapolation across temperature scenarios (Chabni et al. 2024; Mancebo‐Campos et al. 2022).

These quantitative studies demonstrate that even modest temperature reductions during distribution and retail can substantially extend the retention of phenolic compounds, a practical consideration that merits integration into cold‐chain design for antioxidant‐rich products. However, the relationship between temperature and antioxidant stability is not strictly monotonic for all plant‐based matrices. In fresh produce, excessively low temperatures can induce chilling injury, manifested as membrane disruption, enzymatic browning, and oxidative stress, which paradoxically accelerates antioxidant losses; optimal storage ranges must therefore balance chemical kinetic favorability with the physiological tolerance window of each commodity (Neri et al. 2020; Sati et al. 2025). This dual constraint illustrates why purely kinetic predictions require commodity‐specific validation, as discussed in Section 5.2.

Water content and its thermodynamic expression, water activity (*a_w_*), exert a complex, nonlinear influence on antioxidant stability during storage. In processed products, higher water activity promotes oxidative and hydrolytic reactions via increased molecular mobility and oxygen diffusion; lowering water content generally retards these reactions, though overly dry conditions may create pores that facilitate oxygen ingress (Lima et al. 2023). At the mechanistic level, water activity (*a_w_*) governs antioxidant degradation through competing phenomena linked to the glass transition of the food matrix. In persimmon peel powder, the critical *a_w_* triggering the glassy‐to‐rubbery transition at 20°C was 0.211; below this threshold, restricted molecular mobility preserved soluble tannin content and antioxidant capacity (FRAP, DPPH), whereas above *a_w_* = 0.520, accelerated reactant diffusion led to significant losses in phenolic compounds and antioxidant activity. Notably, total carotenoid content followed a U‐shaped degradation pattern, with greater losses at both the lowest and highest *a_w_* values, reflecting the dual role of water: a protective monolayer against oxygen at intermediate moisture and reaction medium at higher moisture (Hosseininejad et al. 2024). Complementarily, in yellow cassava flour, β‐carotene degradation during storage followed first‐order kinetics with activation energies of 85.8–124.2 kJ/mol depending on the drying method, and GAB‐modeled moisture sorption isotherms provided the critical *a_w_* thresholds for packaging selection (Akonor et al. 2023). These compound‐ and matrix‐specific nonlinearities indicate that shelf‐life predictions based solely on temperature may misestimate antioxidant retention if moisture is not co‐modeled.

Fresh produce requires high relative humidity to maintain tissue integrity; dehydration accelerates respiration and enzymatic browning, indirectly exposing antioxidants to degradation (Jung et al. 2023). On the other hand, light exposure is also a major driver of antioxidant degradation during storage, especially for compounds sensitive to photochemical reactions. Anthocyanins, carotenoids, and chlorophyll derivatives are photosensitive; UV–visible light triggers isomerization, structural changes, and photooxidation, diminishing color and antioxidant capacity. Even non‐chromophoric phenolics can degrade via photosensitized reactions. Controlling illumination and using light‐barrier packaging are essential, especially for pigment‐rich products (Mahdavian 2024; Wijesekara and Xu 2024). Quantitatively, in extra virgin olive oil enriched with olive leaf PPs, TPC decreased by up to 36% after 6 months under continuous light exposure, whereas dark‐stored controls retained 95%–98% of their initial PP levels, with light and oxygen identified as the primary drivers of oxidative and photosensitized depletion (Safarzadeh Markhali and Teixeira 2024).

Atmosphere (O_2_ and CO_2_) is another factor that can affect phenolic compounds. Oxygen drives autoxidation and enzyme‐mediated losses of phenolics, anthocyanins, carotenoids, and tocopherols; effects depend on both concentration and diffusion into the matrix (Y. Zhou et al. 2025). Carbon dioxide indirectly slows degradation by reducing oxygen availability, inhibiting oxidative enzymes, and altering local pH, but excessive CO_2_ can damage fresh tissues. Vacuum and modified‐atmosphere packagings are effective tools, often as impactful as temperature control, for preserving antioxidants (Andrigo et al. 2026; Pasquet et al. 2024). For example, in lingonberry jams stored at 4°C and 25°C for 180 days under light and dark conditions, the stability of total phenolics and anthocyanins was significantly higher at lower temperatures and in the dark, with first‐order kinetics established for anthocyanin degradation and rate constants increasing with both temperature and light exposure (Scrob et al. 2022).

Lastly, packaging systems serve as a primary interface between the food and its environment, directly modulating all the factors described above. Among packaging properties, the gas barrier characteristics of the packaging film, particularly the OTR, constitute critical determinants of antioxidant degradation kinetics during storage, as they govern the rate of oxygen ingress into the headspace and, consequently, the extent of autoxidation reactions. The practical relevance of film selection has been experimentally demonstrated in plant‐based matrices: Azarpazhooh et al. (2025) showed that sunflower microgreens (*Helianthus annuus*) packaged in polypropylene film under active modified atmosphere (5% O_2_, 5% CO_2_, 90% N_2_) and stored at 4°C exhibited significantly higher retention of ascorbic acid, total phenolic compounds, chlorophyll, and DPPH radical‐scavenging capacity compared with microgreens packaged in low‐density polyethylene or under passive atmosphere conditions (*p* < 0.05), underscoring the joint influence of film permselectivity and headspace gas composition on the preservation of endogenous antioxidants. Earlier, Sonar et al. (2019) quantitatively demonstrated that thermally pasteurized carrot purée stored in high‐barrier pouches (OTR ≈ 1 cm^3^/m^2^/day) retained up to 89% of ascorbic acid and exhibited significantly higher β‐carotene stability over 100 days of storage at 4–13°C compared with low‐barrier pouches (OTR ≈ 81 cm^3^/m^2^/day), with ascorbic acid degradation following first‐order kinetics and activation energies ranging from 20.3 to 72.3 kJ/mol depending on packaging type. Collectively, these data confirm that the choice of packaging barrier properties represents a decisive lever for controlling the oxidative degradation kinetics of antioxidants in plant‐based food matrices. In practice, selecting packaging with an OTR matched to the product's oxygen consumption rate determines whether the headspace becomes sufficiently anoxic to preserve plant antioxidants or remains oxygen‐rich, promoting oxidative degradation.

Beyond static barrier properties, dynamic headspace modeling integrates package oxygen permeation, produce respiration, and oxidative consumption within the food matrix to predict in‐pack O_2_ and CO_2_ trajectories over time. Herrera et al. (2024) applied this approach to perforation‐mediated MAP of purple passion fruit (*Passiflora edulis* Sims), coupling mathematical simulations of fruit respiration kinetics, multilayer film permeability, and perforation geometry to configure packages that reached favorable equilibrium gas levels during refrigerated storage. Complementarily, Steensma et al. (2025) demonstrated that even low‐intensity retail lighting can partially restore normal atmospheric compositions inside MAP‐stored fresh‐cut lettuce through residual photosynthesis, reactivating senescence‐associated transcriptomic and metabolomic pathways, including the phenylpropanoid route central to surface discoloration, showing that in‐package atmosphere is dynamically responsive to environmental stimuli beyond temperature. These findings reinforce that package OTR should be incorporated as a coupled, temperature‐dependent variable in shelf‐life kinetic models rather than treating headspace composition as a fixed boundary condition, particularly under the non‐isothermal conditions of real supply chains, where film permeability and respiration rates shift concurrently with temperature (Herrera et al. 2024; Steensma et al. 2025).

Light‐transmitting materials hasten photodegradation of chromophoric antioxidants, whereas light‐barrier films and UV‐absorbing coatings protect pigments; packaging may also modulate ethylene accumulation and introduce or sequester transition metals depending on material composition. Accordingly, selecting barrier properties and active/functional packaging systems and integrating them into predictive models is essential to slow oxidation processes across diverse food formats (Lisboa et al. 2024; Sharma et al. 2023).

### 5.2 Kinetic Modeling

Because antioxidants delay oxidation, their persistence is directly linked to shelf life. Degradation is quantified via storage studies and interpreted with kinetic models to predict behavior under different scenarios. Three complementary approaches inform shelf‐life estimation:

- Real‐time studies track products through the supply chain, capturing temperature abuse, humidity fluctuations, light exposure, and packaging heterogeneity. Sensor networks, data loggers, and IoT‐enabled time–temperature indicators (TTIs) yield rich datasets that improve predictive models and risk assessment. These studies provide high external validity but are slow, costly, and can be difficult to interpret in the face of occasional yet impactful deviations (J. M. Costa and Marra 2024; Maheshwari et al. 2025).
- Laboratory accelerated studies isolate variables under controlled conditions, applying elevated temperature, oxygen partial pressure, or light intensity to accelerate degradation and obtain kinetic parameters in shorter timeframes (Muniandy et al. 2023; Y. Zhou et al. 2025). These studies are essential for obtaining rate constants and activation energies that can be extrapolated to milder storage conditions; however, care must be taken to ensure that the acceleration factor does not shift the dominant degradation mechanism (e.g., inducing nonenzymatic browning or structural transitions in the matrix that do not occur at ambient temperatures), which would invalidate extrapolation. For example, Biagini et al. (2024) showed that resveratrol in food supplement tablets exhibited super‐Arrhenius behavior across the 25–40°C range, with matrix‐dependent degradation mechanisms at elevated temperature producing nonlinear Arrhenius plots and overestimating the rate of loss at ambient conditions, and showing that accelerated testing does not provide a universally reliable model for PP shelf‐life prediction.
- Kinetic modeling formalisms. Simple kinetic models of zero‐order (*C* = *C*_0_ − *kt*), first‐order (ln *C* = ln *C*_0_ − *kt*), and second‐order (1/*C* = 1/*C*_0_ + *kt*) assume a single dominant degradation pathway and often fit purified or compositionally simple systems well (Bayram and Decker 2024; Suhag et al. 2025). The majority of antioxidant degradation reactions in plant‐based foods, including thermal and oxidative losses of PPs, anthocyanins, carotenoids, and ascorbic acid, follow apparent first‐order kinetics under isothermal conditions, with the rate constant *k* reflecting the combined influence of temperature, pH, oxygen availability, water activity, and matrix composition (Akonor et al. 2023; Modesto Junior et al. 2023; Scrob et al. 2022; Suhag et al. 2025).

The temperature dependence of *k* is classically described by the Arrhenius equation:

$$k=A\;\exp\left(-E_{a}/RT\right)$$

where *A* is the pre‐exponential (frequency) factor, *E_a_* the activation energy (kJ/mol), *R* the universal gas constant (8.314 J/mol/K), and *T* the absolute temperature (K). Linearization of this relationship (ln *k* vs. 1/*T*) enables estimation of *E_a_* from isothermal degradation data at multiple temperatures.

In the context of plant‐based antioxidants, *E_a_* provides an interpretive framework for understanding the thermal sensitivity of different compound classes: Lower *E_a_* values indicate reactions that are relatively insensitive to temperature changes, whereas higher *E_a_* values indicate reactions that accelerate sharply with modest temperature increases. Reported *E_a_* values differ substantially among antioxidant classes and food matrices.

For anthocyanins in fruit juices and extracts, reported *E_a_* values for thermal degradation remain strongly matrix‐dependent. First‐order kinetics applied to grumixama berry (*Eugenia brasiliensis*) extracts yielded an *E_a_* of 52.7 kJ/mol (60–80°C), consistent with the moderate thermolability of its cyanidin‐ and delphinidin‐3‐glucoside profile (Modesto Junior et al. 2023). In blood orange juice, a Weibull–Log‐Logistic model outperformed classical first‐order fitting during pasteurization at 60–90°C, revealing that ascorbic acid fortification improved initial anthocyanin retention yet increased thermal sensitivity over prolonged heating (Remini‐Sahraoui et al. 2024). Y. Zhang, Zheng, et al. (2025) further demonstrated that heat‐transfer mode modulates the apparent *E_a_*: Microwave volumetric heating yielded a higher *E_a_* for anthocyanin degradation in berry purée than convective or conductive heating at equivalent temperatures. This variability reflects the combined influence of pH‐dependent structural equilibria, glycosylation and acylation patterns, endogenous copigmentation, and matrix composition (Idir et al. 2025).

For carotenoid degradation during storage, *E_a_* values are similarly matrix‐dependent and span an even broader range: from as low as 5–21 kJ/mol for lycopene in vacuum‐fried papaya chips to 66–80 kJ/mol for β‐carotene in encapsulated systems (Lavelli and Sereikaitė 2022), and up to 86–124 kJ/mol for β‐carotene in dried cassava flour (Akonor et al. 2023). This order‐of‐magnitude variation arises because crystalline carotenoid deposits in intact chromoplasts resist oxidation far more than molecularly dissolved carotenoids in lipid phases, and because the physical state of the carrier, conjugation length, co‐dissolved antioxidants, and pro‐oxidant metal availability jointly shift the balance between thermal isomerization and oxidative chain cleavage (Meléndez‐Martínez et al. 2023).

Ascorbic acid *E_a_* values span a wide range depending on the food matrix and dominant degradation pathway, from approximately 20 kJ/mol in high‐moisture, oxygen‐permeable systems (Dhakal et al. 2018) to over 120 kJ/mol in low‐moisture matrices where diffusion limitations constrain the reaction (Akonor et al. 2023). Mechanistically, the aerobic pathway, catalyzed by trace Cu^2+^/Fe^3+^ and dominant at high *a_w_*, exhibits a comparatively low activation barrier. In contrast, the anaerobic hydrolytic route prevalent in low‐moisture matrices imposes diffusion‐limited constraints, thereby raising the apparent *E_a_* (Suhag et al. 2025). This wide intra‐ and inter‐class variability in *E_a_* indicates that shelf‐life predictions cannot be generalized across antioxidant classes or food matrices without compound‐ and matrix‐specific kinetic characterization. These ranges are broadly consistent with the kinetic compilations reported by Suhag et al. (2025) and Tarlak (2023). However, neither review disaggregated *E_a_* values by food‐matrix type (e.g., juice vs. dried product vs. emulsion) or by the physical state of the antioxidant (dissolved vs. crystalline vs. encapsulated), a distinction that the present review highlights as mechanistically critical for accurate shelf‐life extrapolation. The *Q*_10_ concept, the factor by which the rate constant increases per 10°C rise, provides a practical rule‐of‐thumb for supply chain management; for most antioxidant degradation reactions in plant‐based foods, *Q*_10_ values typically fall between 1.5 and 4.0, although outliers exist for highly thermosensitive compounds in specific matrices (Chabni et al. 2024; Sonar et al. 2019).

However, real food products seldom experience purely isothermal conditions throughout the supply chain. Non‐isothermal kinetic approaches address this gap by accounting for time‐varying temperature profiles. The most widely applied framework integrates the Arrhenius equation with the recorded temperature history *T*(*t*) of the product to solve the differential degradation rate equation *d*C/*dt* = −*k*[*T*(*t*)]·C numerically over the measured temperature profile. This approach converts a dynamic thermal exposure into an equivalent isothermal shelf‐life estimate and has been applied to quality prediction in stored fresh produce and fruit‐based products (W. Zhang, Luo, et al. 2021). Time–temperature indicators (TTIs), such as enzymatic, chemical, or microbiological devices whose response follows known kinetics with a defined *E_a_*, serve as practical tools for real‐time non‐isothermal shelf‐life estimation by accumulating the thermal history experienced by individual packages throughout the supply chain (Albrecht et al. 2020).

In real foods, multiple reactions, such as oxidation, hydrolysis, isomerization, and polymerization, occur concurrently and respond differently to temperature, oxygen, water activity, pH, and transition‐metal availability; nonlinear behavior is therefore common, and single‐equation models can underperform. In such cases, multicomponent or empirical nonlinear models that incorporate parallel or sequential reactions, matrix effects, or diffusion limitations provide more realistic predictions. Among these, the Weibull model has gained attention for antioxidant degradation in heterogeneous food systems, because its shape parameter accommodates both concave and sigmoidal decay profiles that cannot be captured by simple first‐order fits (L. Z. Deng et al. 2022; Robichon et al. 2025). Furthermore, coupling kinetic models with mass‐transfer equations, for example, incorporating oxygen diffusion through the food matrix and the packaging film simultaneously, enables prediction of spatial gradients of antioxidant loss within packaged products, which is particularly relevant for solid and semi‐solid plant‐based foods where surface layers degrade faster than the bulk interior (Ferreiro et al. 2025; Rashvand et al. 2025).

Importantly, the physical state of the food matrix (i.e., liquid, semi‐solid, or solid) imposes distinct mass‐transfer constraints that fundamentally shape antioxidant degradation kinetics during storage. In liquid systems (juices, beverages, plant milks), homogeneous mixing provides unrestricted access of dissolved oxygen and pro‐oxidant transition metals to antioxidant molecules, and degradation is generally well described by bulk‐phase rate equations where the observed *k* reflects intrinsic chemical reactivity under uniform conditions (Suhag et al. 2025). In semi‐solid matrices (purées, gels, jams, fermented pastes), restricted molecular mobility introduces diffusion gradients in oxygen, substrate, and product concentrations; consequently, surface layers exposed to headspace oxygen degrade substantially faster than the interior, creating spatial heterogeneity that requires coupled reaction–diffusion models for accurate kinetic prediction (Ferreiro et al. 2025). In solid or dried systems (powders, flours, extrudates, freeze‐dried matrices), the glass transition temperature of the amorphous matrix becomes a critical variable: Below *T_g_*, severely restricted molecular mobility preserves antioxidant integrity, whereas above *T_g_*, the glassy‐to‐rubbery transition sharply increases reactant diffusion and degradation rates, often leading to non‐first‐order profiles better captured by Weibull or stretched‐exponential formalisms (Hosseininejad et al. 2024). Furthermore, in porous dried matrices, porosity and pore connectivity determine the rate of oxygen ingress from the headspace to the interior, so that the interplay between film OTR and matrix porosity jointly governs the effective oxygen exposure of embedded antioxidants (Rashvand et al. 2025). These matrix‐state‐dependent kinetic behaviors reinforce that kinetic parameters obtained in model solutions cannot be directly extrapolated to real food products without accounting for the transport limitations imposed by the specific physical state of the matrix.

Artificial intelligence and machine learning approaches are increasingly being integrated with traditional kinetic frameworks to improve shelf‐life prediction. Neural networks, random forests, and support vector regression have been applied to predict quality deterioration and antioxidant losses in stored plant‐based products by training on multi‐variable datasets encompassing temperature, oxygen, humidity, light, and matrix composition. These data‐driven methods can capture complex, nonlinear interactions among degradation drivers that conventional parametric models may not adequately represent, and they can assimilate real‐time sensor data for adaptive prediction along the supply chain (Liang et al. 2026; Parra‐Escudero et al. 2025). However, their predictive accuracy is constrained by the quality and representativeness of training datasets, and the lack of mechanistic interpretability remains a limitation when extrapolating beyond the experimental domain. Hybrid approaches that embed physics‐based kinetic constraints within machine learning architectures offer a promising path toward models that are both flexible and mechanistically grounded (Liang et al. 2026; Parra‐Escudero et al. 2025).

Therefore, a robust workflow for shelf‐life prediction of antioxidant‐rich plant‐based foods combines (i) isothermal controlled‐condition datasets at multiple temperatures for kinetic parameter estimation (*k*, *E_a_*), (ii) accelerated testing under intensified stressors to validate the extrapolation range and identify mechanism shifts, and (iii) real‐time validation with continuous temperature monitoring and TTIs under commercial distribution conditions.

Packaging and storage set points should be selected with direct reference to the fitted degradation kinetics, specifically, the *E_a_* and *Q*_10_ of the rate‐limiting antioxidant compound, so that temperature, oxygen, and light are actively managed based on quantitative predictions rather than assumed fixed values. Integrating packaging OTR into the kinetic framework and employing dynamic headspace models further refines these predictions for MAP and respiring products. This integrated approach improves shelf‐life reliability and supports matrix‐specific decisions that safeguard antioxidant content and overall product quality (Suhag et al. 2025).

Several limitations of the current kinetic literature on plant‐based antioxidant degradation during storage merit acknowledgment. First, the majority of published kinetic studies have been conducted on model systems or single‐commodity matrices under isothermal laboratory conditions, and their extrapolation to multi‐component, commercially packaged foods experiencing dynamic temperature profiles remains insufficiently validated. Second, kinetic parameters are typically derived from TPC or total anthocyanin measurements rather than from individual compound tracking, which obscures compound‐specific degradation pathways and may mask concurrent formation of degradation products with residual antioxidant activity. Third, the interaction between simultaneous degradation mechanisms, such as oxidation, hydrolysis, and polymerization, is rarely explicitly modeled; most studies fit apparent single‐pathway kinetics, potentially biasing *E_a_* estimates. Fourth, water activity and oxygen partial pressure are seldom co‐varied with temperature in factorial experimental designs, limiting the ability to construct multi‐factor predictive models. Finally, standardization of kinetic reporting remains inconsistent across the literature: Units for rate constants, temperature ranges investigated, and statistical goodness‐of‐fit criteria vary substantially among studies, complicating meta‐analytical comparisons. Most importantly for translational relevance, kinetic models calibrated on disappearance of the parent antioxidant do not track the appearance of microbial catabolites (phenyl‐γ‐valerolactones, urolithins, hydroxyphenyl‐propionic acids), which are the species that account for systemic exposure in humans (Di Pede et al. 2023; Parmenter et al. 2023; Pidgeon et al. 2025); shelf‐life metrics derived from parent‐compound *k* and *E_a_* therefore predict food‐quality outcomes but not consumer‐relevant bioactive delivery.

The kinetic descriptors obtained from storage studies (*k*, *E_a_*, and the compound‐specific *Q*_10_) also constrain upstream process design, because they define the thermal budget that each antioxidant class can absorb before unacceptable losses occur (Section 4). Combined with the protocols of isothermal characterization, accelerated testing, and real‐time monitoring with time–temperature integrators, these descriptors convert the analytical profile of an antioxidant‐rich product into quantitative shelf‐life predictions for use in formulation and packaging selection.

## 6 Matrix Interactions in Food Systems

**FIGURE 4:**
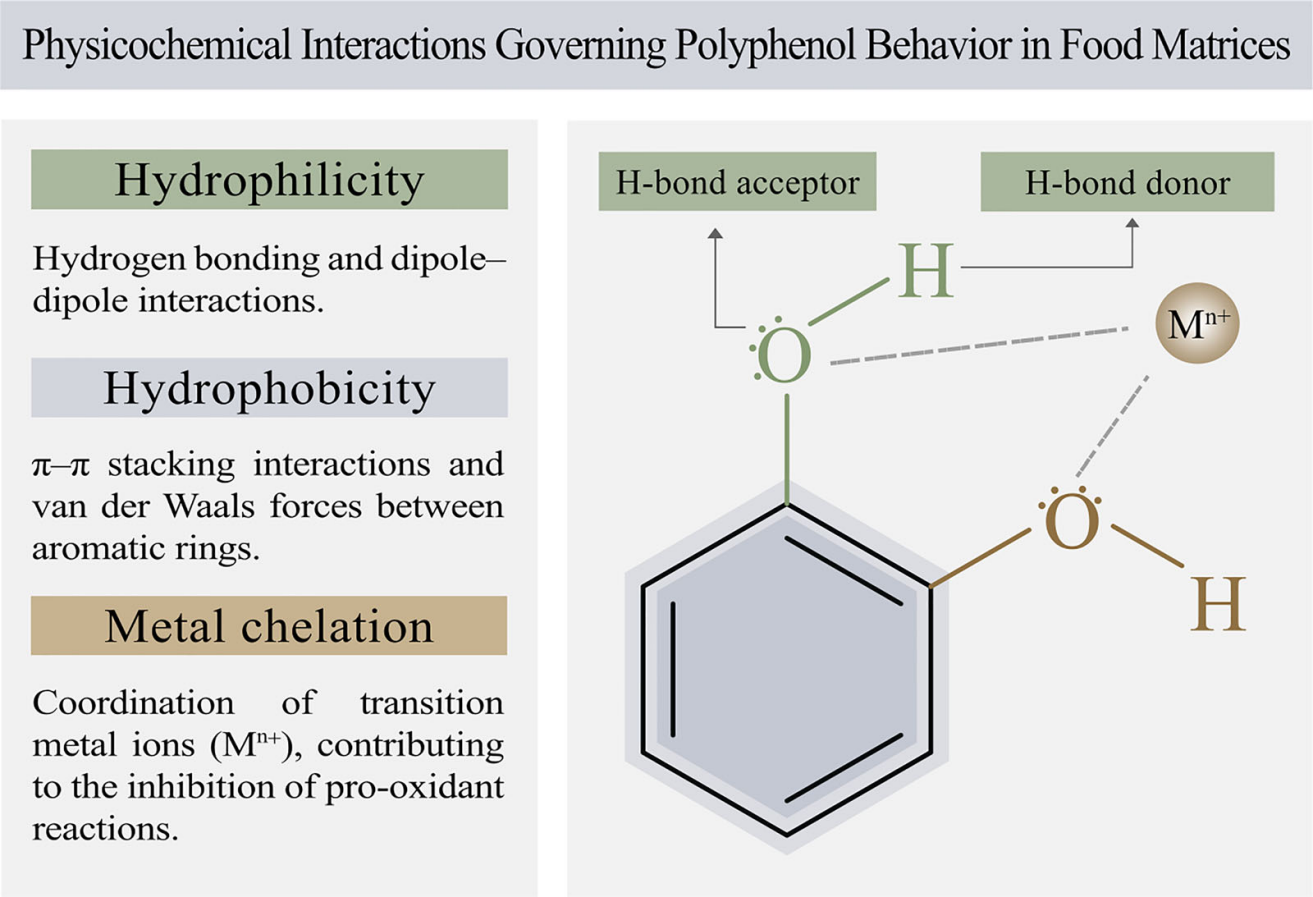
Physicochemical interactions governing polyphenol behavior in food matrices. Representative schematic illustrating the main physicochemical interactions governing the behavior of plant‐based polyphenols in food matrices. The structure shown is a generalized phenolic to highlight interaction sites rather than represent a specific compound.

In multiphase foods, antioxidant performance depends on intrinsic reactivity together with partitioning and residence time within matrix microenvironments (aqueous, lipid, and interface phases). Polarity, pH, ionic strength, and interfacial density modulate ionization, mobility, and accessibility, creating either beneficial retention (e.g., PP–PS complexes) or unintended sequestration that reduces the active fraction (Figure 4) (Manzoor et al. 2024; Xue et al. 2024). PP interactions are primarily classified into four categories: proteins, PSs, lipids (including colloidal structures such as O/W emulsions), and minerals (including transition metals). Throughout the following subsections, it is important to recognize that different analytical methods capture distinct chemical phenomena: Solution‐phase assays (DPPH, ABTS, FRAP) primarily reflect electron‐transfer or hydrogen‐atom‐transfer capacity under homogeneous conditions, whereas in situ measurements of lipid hydroperoxide formation, conjugated‐diene accumulation, or hexanal generation assess antioxidant performance within heterogeneous matrices where partitioning and interfacial dynamics govern efficacy (Losada‐Barreiro et al. 2024). Activity rankings may therefore differ substantially between these approaches for the same antioxidant–matrix combination.

Critically, the structural rules established in Sections 2 and 3, such as the Bors criteria for flavonoids or the polyene conjugation length governing carotenoid quenching efficiency, do not translate linearly into real food matrices, because partitioning between phases, competitive binding to biopolymers, and local physicochemical conditions (dielectric constant, interfacial curvature, and proton availability) impose constraints that can amplify, attenuate, or even invert the activity ranking predicted from homogeneous‐phase assays (Losada‐Barreiro et al. 2024).

Furthermore, although PPs dominate the discussion of matrix interactions due to their mechanistic diversity and the depth of available literature, it should be acknowledged that bioactive peptides derived from enzymatic hydrolysis of plant proteins (e.g., from soybean and hemp seed) (Montserrat‐de la Paz et al. 2023; Rebollo‐Hernanz et al. 2023) and certain antioxidant mono‐ and PSs also contribute to the antioxidant capacity of plant‐based food systems through radical scavenging, metal chelation (Yap and Gan 2024), and synergistic or competitive interactions (Peng et al. 2023) with PPs at interfaces. These additional classes are acknowledged here to provide a comprehensive view; PPs remain the focal class in the subsections below.

The present review focuses on PPs, carotenoids, and tocopherols/tocotrienols, which together account for the majority of endogenous antioxidants in plant‐based foods and have the most developed structure–activity, kinetic, and matrix‐interaction literatures. Antioxidant peptides and PSs, also relevant in some plant‐based matrices, are reviewed in detail elsewhere (Montserrat‐de la Paz et al. 2023; Yap and Gan 2024).

### 6.1 PPs–Proteins Interactions

PPs bind proteins through reversible hydrogen bonding, hydrophobic/van der Waals forces, and electrostatic attraction governed by phenolic hydroxylation, protein conformation, and pH relative to the protein's p*I* (B. Ma et al. 2024; Tomas et al. 2025; Xue et al. 2024). These interactions can lower the free PP pool at oil/water (O/W) interfaces and densify films, thereby hindering pro‐oxidant ingress. Covalent coupling arises when catechols oxidize to quinones, which then react with lysine/cysteine, increasing film viscoelasticity and thermal stability, and altering solubility, isoelectric point, and dispersibility. Functionally, PPs–protein complexes often yield thicker, more elastic interfacial layers that slow lipid oxidation, and the magnitude of the resulting protection scales with binding affinity rather than with bulk antioxidant load. In pea‐protein‐isolate (PPI) emulsions, the strength of noncovalent PPI–PP binding, assessed by intrinsic‐fluorescence quenching and supported by molecular‐dynamics simulations, increased systematically with the number of phenolic hydroxyl groups available for interfacial anchoring across the PPs tested, with concomitant improvements in emulsion stability and reductions in lipid hydroperoxide formation during 10‐day storage (Tang et al. 2025). Comparable structure‐dependent stabilization has been reported for zein‐based systems below the protein's p*I*, where electrostatic and hydrophobic constraints restrict pro‐oxidant metal migration to the interface (Manzoor et al. 2024; Xue et al. 2024).

The functional consequences of protein binding for antioxidant performance in food matrices deserve explicit analysis because they reveal how structural chemistry translates into (or departs from) predicted activity. Protein–PP complexes (both covalent and noncovalent) can exhibit enhanced antioxidant activity compared with either component alone, attributed to conformational changes in the protein that expose additional electron‐donating residues and to stabilization of the PP against autoxidation (Liu et al. 2021).

Quantitative emulsion‐oxidation data in this review illustrate the functional consequence of protein–PP complexation at the interface. In the ovalbumin–procyanidin system, the oxidation degree of the emulsion was reduced from 5.90% to 1.78% at Day‐6 of storage (Wen et al. 2022). Quantitatively analogous protection has been reported for soy protein hydrolysate covalently modified with EGCG, in which the conjugate displayed both higher emulsifying activity and reduced primary and secondary oxidation product formation versus unmodified hydrolysate during accelerated storage (T. Wu et al. 2023). In PPI emulsions over 10 days, the rosmarinic‐acid–PPI complex produced the smallest droplet size, the highest absolute *ζ*‐potential, and the lowest lipid‐hydroperoxide accumulation among five tested PPs, with the rank order tracking the eight phenolic hydroxyls of rosmarinic acid versus the two of caffeic acid; intrinsic‐fluorescence quenching constants (*K*_sv) and binding affinities (*K*_*a*) increased monotonically with hydroxyl number, providing molecular‐level confirmation that interfacial anchoring scales with hydroxylation, not with bulk DPPH ranking (Tang et al. 2025).

The dependence of this protective effect on PP architecture has been further substantiated in PPI emulsions, in which the magnitude of noncovalent PPI–PP binding (assessed by intrinsic‐fluorescence quenching and molecular‐dynamics simulations) increased with the number of phenolic hydroxyl groups available on the PP, with concomitant improvements in emulsion stability and reduced lipid oxidation. The additional hydroxyls were proposed to anchor the PP more firmly within the interfacial protein film (Tang et al. 2025). This molecular‐level evidence consolidates the interpretation that interfacial film density and PP architecture, not bulk PP load, govern oxidative protection in protein‐stabilized emulsions.

PPs can also inhibit digestive enzymes such as pepsin and trypsin, thereby protecting bioactive peptides from premature degradation and enabling targeted intestinal release, a phenomenon that connects matrix interactions to the bioaccessibility considerations discussed in Section 6.6.

### 6.2 PPs–PSs Interactions

PP–PS interactions mirror those above but are typically more diffuse and reversible because of PS flexibility and hydration (Liu et al. 2020). Hydrogen bonding, hydrophobic, and electrostatic forces dominate; PS structure (e.g., degree of methylation in pectin, molecular weight, and branching in arabinogalactans) and PP galloylation/hydroxylation drive affinity. These complexes can increase PP solubility, protect against degradation, and modulate bioaccessibility in fiber‐rich foods (Bermúdez‐Oria et al. 2020).

PP–PS interactions are strongly structure‐dependent and should not be generalized as uniformly protective in plant‐based food matrices (Fernandes et al. 2020; L. Hu et al. 2025). In pectin‐rich systems, degree and pattern of esterification, homogalacturonan content, and matrix conditions such as pH, ionic strength, and thermal history govern noncovalent association and, consequently, modulate color stabilization, oxidation behavior, and processing stability (Fernandes et al. 2020; J. Hu et al. 2023; Morales‐Medina et al. 2022). Available evidence further indicates that low‐methyl‐esterified or homogalacturonan‐rich pectic fractions may favor anthocyanin stabilization, whereas in calcium‐cross‐linked low‐methoxyl matrices, gel texture becomes a key determinant of flavonoid release kinetics (Fernandes et al. 2021; Jantrawut et al. 2013).

In fiber‐rich plant matrices, particularly cereal brans, a substantial proportion of PPs remains in non‐extractable, fiber‐associated forms; consequently, assessments focused mainly on soluble fractions may underrepresent the antioxidant contribution of the intact matrix (Y. Zhang, Bai, et al. 2025; Y. Zhang, Bai, et al. 2024). The PS‐mediated stabilization can be quantitatively decisive: In dark chocolate and red‐skinned onion, INFOGEST whole‐food bioaccessibility of flavan‐3‐ols and flavonols (≈80%) was 1.4–1.7‐fold higher than that of the corresponding phenolic extracts (47.3% chocolate; 57.5% onion), demonstrating that the food matrix protects rather than only restricts release (Cattivelli et al. 2025). In water‐soluble dietary‐fiber matrices (pectin, guar, alginate), by contrast, controlled human studies have shown that β‐carotene plasma response is reduced relative to fiber‐free controls, whereas water‐insoluble fibers exert a smaller restrictive effect, establishing that PS chemistry, not PS mass alone, governs matrix‐antioxidant outcome (Shukla et al. 2025).

Experimental digestion–fermentation models further indicate that release is limited in the upper gastrointestinal tract but increases markedly during colonic fermentation, as shown for bound phenolics from oat bran and barley (Y. Zhang, Bai, et al. 2025; Zhao et al. 2025). Notably, the residual fiber‐bound fraction can retain relevant antioxidant or carbonyl‐scavenging activity during fermentation, suggesting sustained local redox protection in the lower gut (J. Li et al. 2023; Y. Zhang, Bai, et al. 2024). Mechanistically, this delayed release appears to depend on microbiota‐associated hydrolytic activities, providing a direct link to the bioaccessibility framework discussed in Section 6.6.

### 6.3 PPs–Lipids and Colloidal Structures (O/W Emulsions) Interactions

Interfacial localization controls efficacy because lipid oxidation initiates at the oil–water boundary. The polar paradox, less‐polar antioxidants perform better in emulsions and more‐polar in bulk oils, often breaks down in real systems; microstructures (surfactant micelles, protein films, and colloidal aggregates) redistribute antioxidants, so effective interfacial concentration, not simple polarity, predicts performance. Cut‐off effects show that increasing amphiphile chain length enhances efficacy only up to a critical point, after which antioxidants bury in the oil phase and lose interfacial action (Barouh et al. 2022; Berton‐Carabin and Villeneuve 2023; McClements and Decker 2018). Lastly, emulsifier choice matters: Proteins create thick, heterogeneous barriers that retain PPs and slow pro‐oxidant access; dense nonionic surfactants may sequester antioxidants in aqueous micelles, reducing interfacial availability. Pickering particle‐stabilized interfaces can be even more oxidation‐resistant due to irreversible adsorption and restricted diffusion (C. Chang et al. 2023; Hinderink et al. 2022; McClements and Decker 2018).

The mechanistic basis of these phenomena has been elucidated through the pseudophase kinetic model, which quantifies antioxidant partitioning between oil, aqueous, and interfacial pseudophases in intact emulsions and yields effective interfacial concentrations 15‐ to 200‐fold above the stoichiometric (bulk) value, depending on antioxidant chemistry and surfactant volume fraction (M. Costa et al. 2021; Losada‐Barreiro et al. 2024). For α‐ and δ‐tocopherol in 1:9 (v/v) soybean and olive O/W emulsions at acidities below their p*K*_*a*, effective interfacial concentrations are 15‐96‐fold above bulk and δ‐tocopherol partitions more strongly to the interface than α‐tocopherol, a result that inverts the homogeneous‐phase BDE ranking and exemplifies how phase distribution overrides intrinsic reactivity (Fernández‐Ventoso et al. 2022).

Recent evidence indicates that the effective interfacial concentration mainly determines antioxidant efficacy in O/W emulsions. In fish‐oil O/W emulsions and nanoemulsions, the pseudophase kinetic model applied to homologous series of hydroxytyrosol alkyl esters has quantified interfacial enrichment: Effective antioxidant concentration in the interfacial region is 20–200‐fold higher than the stoichiometric (bulk) concentration, and induction times for conjugated‐diene formation correlate linearly with the interfacial concentration rather than with bulk dose (M. Costa et al. 2021; Losada‐Barreiro et al. 2024). For chlorogenic acid esters, the interfacial enrichment factor is lower (20–90‐fold), mechanistically explaining their comparatively modest antioxidant efficiency in fish‐oil nanoemulsions despite high intrinsic reactivity, a direct example in which the pseudophase model corrects the misleading prediction of solution‐phase assays. The interfacial enrichment factor is governed primarily by the surfactant volume fraction and secondarily by the oil/water ratio, whereas droplet size has negligible effect, confirming that antioxidant partitioning is thermodynamic rather than geometric in origin.

By contrast, in protein‐stabilized emulsions, the thick interfacial film provides additional binding sites for PPs at the oil–water interface. In PPI emulsions monitored over 10 days, suppression of primary lipid hydroperoxide accumulation increased with the number of phenolic hydroxyls in the PP and correlated with PPI–PP intrinsic‐fluorescence quenching strength rather than with the PP's bulk DPPH ranking (Tang et al. 2025). These data show that, in emulsified systems, antioxidant performance is set by the effective interfacial concentration rather than by bulk polarity or nominal dose, with direct implications for formulation design. This principle is best supported when interfacial location is interpreted together with regeneration competence. In oil‐in‐water emulsions, α‐tocopherol combined with myricetin generated interaction indices of 2.44–3.63, whereas α‐tocopherol combined with taxifolin was antagonistic despite close structural similarity between the flavonoids. The difference was not explained by nominal concentration alone, but by the ability of myricetin to regenerate the tocopheroxyl radical under near‐neutral conditions. Accordingly, interfacial colocalization should be treated as necessary but not sufficient; regeneration chemistry and local iron redox behavior must also be considered when predicting synergism (Bayram et al. 2023).

### 6.4 PPs–Minerals Interactions

Trace metals (Fe, Cu, Mn, etc.) accelerate oxidation by directly oxidizing lipids, catalyzing hydroperoxide decomposition, and propagating radicals. Their location (especially at interfaces) is as important as valence: Anionic surfactants concentrate cations at the boundary, increasing peroxide breakdown, whereas chelators or non‐adsorbed proteins can displace metals away from the interface (Berton‐Carabin and Villeneuve 2023; Hinderink et al. 2022).

Catecholic PPs form bidentate complexes with Fe^3+^ that are several orders of magnitude more stable than the corresponding monohydroxyphenol complexes, effectively removing iron from Fenton chemistry under acidic‐to‐mildly acidic pH conditions; the same catechols, however, undergo autoxidation at neutral‐to‐alkaline pH that reduces Fe^3+^ to Fe^2+^ via *o*‐semiquinone intermediates and accelerates lipid hydroperoxide breakdown, generating an explicit pH‐dependent transition between protective chelation and pro‐oxidant cycling (Lomozová et al. 2021; Scarano et al. 2023). Even strong chelators (e.g., EDTA) can paradoxically increase oxidation by mobilizing iron from sequestered pools or by facilitating H_2_O_2_ decomposition, and polyanionic PSs can similarly solubilize metals without inactivating them, a quantitative reminder that chelation capacity measured in homogeneous solution does not predict the net oxidation outcome at lipid–water interfaces (Barouh et al. 2022; Mertens et al. 2022).

The pH‐dependent transition between protective chelation and pro‐oxidant cycling already enumerated in Section 3.5 (inconsistency iii) translates directly into formulation rules. At pH <∼6, catechols form bidentate Fe^3+^ complexes (conditional log *K* ≈ 16–20 for the 1:1 catecholate‐Fe^3+^ adduct) that remove iron from Fenton chemistry and reduce lipid hydroperoxide formation in O/W emulsions (Lomozová et al. 2021; Mertens et al. 2022). At neutral‐to‐alkaline pH the same catechols autoxidize, generating O_2_•^−^ via *o*‐semiquinone intermediates and reducing Fe^3+^ to Fe^2+^, which explains why DPPH/FRAP‐positive flavonols can drive net protein and lipid oxidation in dough and plant‐milk matrices (Bayati and Poojary 2025). The formulation consequence is direct: In acidic matrices (juices, fermented beverages, acidified sauces), catechol‐containing PPs act predominantly as protective chelators; in neutral/alkaline matrices (plant milks, doughs, alkaline‐processed grains), the same compounds may exacerbate metal‐catalyzed oxidation unless co‐formulated with ascorbate or other reducing agents that hold iron in the ferrous state (Liu et al. 2025; Mertens et al. 2022). At the interfacial level, multilayer (lecithin–chitosan) or protein‐based emulsifiers carrying a positive surface charge displace cations from the droplet surface and reduce interfacial metal loading (C. Chang et al. 2023; McClements and Decker 2018).

At the interfacial level, transition‐metal management is equally decisive. In O/W emulsions stabilized by anionic surfactants (e.g., SDS, lecithin), electrostatic attraction concentrates Fe^2+^/Fe^3+^ at the negatively charged droplet surface, substantially increasing the local metal concentration relative to the bulk aqueous phase and dramatically accelerating interfacial lipid oxidation (Berton‐Carabin and Villeneuve 2023). Strategies that have proven effective in mitigating this phenomenon include the use of multilayer emulsions (e.g., lecithin primary layer + chitosan secondary layer), which create a positively charged outer barrier that electrostatically repels cations from the oil–water interface, significantly reducing lipid oxidation compared with single‐layer emulsions under identical conditions (C. Chang et al. 2023; McClements and Decker 2018).

### 6.5 Synergy and Antagonism Between Antioxidants in Complex Matrices

To interpret whether antioxidant combinations behave synergistically or antagonistically in foods, it is essential to consider how the actives are retained, released, and allowed to react within their carriers and the surrounding matrix. This subsection briefly examines (i) active biopolymer systems that retain and deliver antioxidants, (ii) stabilization of essential oils and plant extracts via encapsulation, (iii) release kinetics governed by carrier microstructure and environmental conditions, and (iv) antioxidant reactivity toward ROS and lipid radicals, key factors that determine whether combinations manifest synergy or antagonism in foods.

Efficacy in active biopolymer systems arises from the balance among retention, controlled release, and the chemical reactivity of the incorporated antioxidants (Vargas‐Ramella, Echegaray, et al. 2025). In line with this, because many essential oils and plant extracts are volatile, poorly soluble, and thermally/photolabile, nanoencapsulation or incorporation into chitosan, zein, or starch networks increases stability and minimizes losses; notably, nanoencapsulated anthocyanins can raise film thermal resistance via matrix cross‐linking (Hossen et al. 2024). On the other hand, the release kinetics are dictated by film microstructure such as porosity, crystallinity, thickness, and free volume and by environmental factors such as pH, temperature, and humidity (L. Zhang, Yu, et al. 2021).

As a model, PLGA‐chrysin systems exhibit a biphasic profile, with ∼20%–30% of the payload released as an initial burst within the first 24 h followed by sustained Fickian‐diffusion‐controlled release over 7–14 days; the effective diffusion coefficient (*D*_eff) of the sustained phase scales with polymer crystallinity, molecular weight, and the (*T*–*T*_*g*) gap, providing a tunable parameter for synchronizing release kinetics with matrix‐specific radical‐generation rates (Khaledi et al. 2020). This biphasic kinetics is functionally significant: The burst phase delivers an immediate radical‐scavenging response at sites of oxidation initiation, whereas the sustained phase maintains protective concentrations during extended storage (Yoo and Won 2020). The practical implication is that carrier composition must be tuned not only for encapsulation efficiency but also for the temporal synchronization of antioxidant release with the kinetics of radical generation in the specific food matrix, a design parameter that is rarely optimized in current formulation studies but that determines whether multi‐antioxidant systems achieve true synergy or merely additive protection (Losada‐Barreiro et al. 2024).

Ultimately, functional activity depends on the fraction effectively released and its capacity to quench ROS/lipid radicals. Quantitative configuration‐dependence of multi‐antioxidant interactions has been resolved by the WIM‐CAT framework in fish‐oil O/W emulsions: At low tocopherol concentrations (0.2 µM in emulsion, ≈380 ppm in oil) and high PP‐to‐tocopherol molar ratios (3:1), α‐tocopherol–quercetin and γ‐tocopherol–curcumin combinations are clearly synergistic, whereas at threefold higher tocopherol loads the same combinations show attenuated or absent synergy (Robichon et al. 2025, 2026). This dose‐windowed behavior confirms that synergy is not an intrinsic chemical property of the pair but a configuration‐dependent kinetic outcome and provides a quantitative design rule for multi‐antioxidant formulations. By tuning carrier composition and microstructure to synchronize release with sites of radical generation, formulators can steer multi‐antioxidant systems toward true synergy and avoid conditions that manifest as antagonism.

### 6.6 Bioaccessibility, Gastrointestinal Fate, and Translational Relevance

The antioxidant–matrix interactions described in Sections 6.1–6.5 evolve dynamically during digestion, where sequential changes in pH, enzymatic activity, bile salt concentration, and mechanical shear progressively restructure PP–biopolymer complexes and alter antioxidant bioaccessibility. The standardized INFOGEST in vitro digestion protocol (Brodkorb et al. 2019) has become the principal tool for assessing these processes, and its application to PP‐ and carotenoid‐rich foods has been validated in primary studies employing model emulsion systems (Tan, Li, Zhou, et al. 2020).

In the gastric phase (pH 1.5–3.5, pepsin), the strongly acidic environment promotes dissociation of noncovalent PP–protein complexes formed during food processing, increasing the free PP fraction. For anthocyanins, this low pH stabilizes the flavylium cation form, the most redox‐active species, meaning that gastric recovery of anthocyanins from berry and fruit matrices is generally high relative to other flavonoid classes (de Oliveira Sartori et al. 2023). Peptic hydrolysis of food proteins exposes new binding sites that may recapture released PPs, creating a dynamic equilibrium between free and bound forms that complicates predictions based on initial matrix composition alone.

In the intestinal phase (pH 6.5–7.5, pancreatic lipase, bile salts), the critical bottleneck for bioaccessibility of lipophilic antioxidants is micellarization, incorporation into mixed bile salt‐phospholipid‐fatty acid micelles (Tan, Zhang, Muriel Mundo, et al. 2020). Accordingly, in O/W delivery systems, the intestinal bioaccessibility of β‐carotene and related carotenoids is highly formulation‐dependent, with particle‐size reduction and interfacial engineering generally promoting lipolysis and transfer into mixed micelles, whereas carrier‐lipid composition can further modulate micellar solubilization during digestion (Gomes et al. 2023; Lara‐Abia et al. 2023). At 10% oil concentration in a corn oil nanoemulsion system, β‐carotene bioaccessibility reached 93.2%, but it decreased at higher oil concentrations due to precipitation and saturation of the micellar phase (Tan, Zhang, Zhou, et al. 2020). For PPs, the alkaline intestinal pH promotes oxidative degradation of anthocyanins (conversion to chalcone and subsequent ring‐opening products) and favors binding to pancreatic enzymes and bile salts, which can simultaneously reduce the free PP fraction and inhibit enzyme activity (Bešlo et al. 2023; Tomas et al. 2025). This instability should be discussed together with evidence that anthocyanin efficacy in humans is not solely determined by parent‐compound chemistry. In a 24‐week randomized, double‐blind, placebo‐controlled trial in adults at risk of dementia (*n* = 99), supplementation with 320 mg/day anthocyanins improved several systemic biomarkers, including LDL cholesterol (*ηp* ^2^ = 0.078; *p* = 0.015), cardiometabolic score (*ηp* ^2^ = 0.073; *p* = 0.021), CRP (*ηp* ^2^ = 0.417; *p* = 0.0001), IL‐6 (*ηp* ^2^ = 0.085; *p* = 0.015), and IL‐1β (*ηp* ^2^ = 0.058; *p* = 0.037), with stronger CRP reduction in participants who had higher baseline inflammatory burden. These findings indicate that clinically relevant response can emerge despite limited parent‐compound bioavailability, consistent with the broader pattern in which microbial catabolites (protocatechuic acid, gallic acid, syringic acid, and their phenylpropionic derivatives) account for the systemic anthocyanin metabolite pool (Borda et al. 2025; Catalkaya et al. 2020).

Recent evidence also suggests that the efficacy of anthocyanins is phenotype‐dependent. In a separate 12‐week randomized, double‐blind, placebo‐controlled trial in subjects with impaired glucose tolerance (*n* = 68), 160 mg/day anthocyanins did not significantly improve the primary β‐cell endpoint, but they increased the reversion from impaired glucose tolerance to normoglycemia from 29.4% in placebo to 55.9% in the anthocyanin group (*p* = 0.013) and improved the Matsuda insulin‐sensitivity index by an adjusted difference of 4.6 (95% CI 2.5–6.7; *p* = 0.003). This supports a more nuanced translational interpretation: Anthocyanin efficacy is likely to depend on metabolic phenotype, baseline inflammatory status, and host response architecture (Yu et al. 2026).

The colonic phase represents a crucial but often overlooked dimension of PP bioaccessibility. Unabsorbed PPs and PP–fiber complexes undergo microbial metabolism: Gut microbiota cleave glycosidic bonds, ester linkages, and C‐ring structures of flavonoids, generating low‐molecular‐weight phenolic metabolites including hydroxyphenylacetic acids, hydroxyphenylpropionic acids, phenylvalerolactones, and urolithins that are absorbed through the colonic epithelium and exert systemic antioxidant and anti‐inflammatory effects (Catalkaya et al. 2020). The colonic metabolism of PP–fiber complexes is slower and more sustained than that of free PPs, providing prolonged exposure of the colonic mucosa to bioactive metabolites.

From a formulation design perspective, these gastrointestinal dynamics establish three design principles: (i) antioxidant carriers should resist gastric disruption while permitting intestinal release, favoring pH‐responsive systems such as alginate–chitosan or zein–pectin composites; (ii) lipophilic antioxidants (carotenoids, tocopherols) require co‐ingestion with digestible lipids and fine emulsification to maximize micellarization; and (iii) for colon‐targeted delivery, resistant starch and insoluble fiber carriers that resist upper GI digestion are preferred (Liu et al. 2020; Tomas et al. 2025).

However, the majority of current evidence derives from static in vitro digestion models; semi‐dynamic and fully dynamic models (e.g., TIM‐1, SHIME) provide improved physiological relevance but remain limited in reproducing interindividual variability (Brodkorb et al. 2019). Well‐designed human intervention trials that integrate bioaccessibility, bioavailability (plasma/urinary metabolite profiling), and mechanism‐linked biomarkers of oxidative status are ultimately necessary to validate these matrix‐design principles.

A cross‐cutting limitation of the matrix‐interaction literature discussed in previous sections is that the majority of quantitative partitioning and interfacial studies have been conducted in model emulsions or binary biopolymer systems, whose compositional simplicity does not fully replicate the complexity of real multicomponent food matrices. Extrapolation of pseudophase partitioning coefficients or interfacial antioxidant concentrations derived from model systems to commercial foods should therefore be approached with caution, and validation in actual food products under realistic formulation and storage conditions remains a priority for the field.

## 7 Potential Formulations and Synergistic Effects

This section addresses (i) multicomponent antioxidant systems, (ii) the mechanistic basis and molecular interactions that drive synergism, and (iii) structural and concentration‐dependent effects that determine whether mixtures behave synergistically, additively, or antagonistically.

In real food systems, oxidative reactions are spatially heterogeneous processes that occur preferentially at specific sites, such as lipid–water interfaces, dispersed oil droplets, or regions enriched in transition metals. Therefore, the effectiveness of antioxidant combinations depends jointly on intrinsic chemical reactivity and on the ability of the components to partition, localize, and remain active within these microenvironments. This perspective shifts formulation strategies from compound selection to spatially resolved antioxidant design.

Combining plant‐derived antioxidants can outperform single molecules when formulations are tuned to the target food matrix (Vargas‐Ramella et al. 2021). Multicomponent systems help overcome limitations typical of natural extracts, variable composition, poor solubility, interfacial mislocalization, and thermal/photoinstability, by pairing complementary chemistries and using carriers (e.g., protein/PS films, emulsions, and nano/microencapsulation) that protect actives and position them where lipid oxidation initiates (Freitas et al. 2025; Zhong et al. 2025). Although synergy is often reported in complex botanical mixtures, it is not guaranteed; outcomes depend on matrix microstructure, oxygen and transition‐metal availability, dosing, and the analytical readout (Table 2).

**TABLE 2:** Synergistic, additive, and antagonistic interactions among plant‐derived antioxidants in food‐related systems.

Plant‐based antioxidants		Antioxidant synergism	References
*Olea europaea*	*Malus domestica*	Strong enhancement	Manzoor et al. (2024)
*Lycium ruthenicum* Murr.	Epigallocatechin gallate (EGCG)	Strong enhancement	Guan et al. (2025)
Brown propolis	Green propolis	Strong enhancement	J. D. F. Da Silva et al. (2025)
*Cucurbita pepo*	*Sinapis alba*	Strong enhancement	Hawash et al. (2025)
Curcumin	Piperine	Strong enhancement	Tian et al. (2025)
*Camellia sinensis*	*Oryza sativa*	Strong enhancement	Chaisan et al. (2025)
α‐Tocopherol, β‐carotene	Tea polyphenols palmitate (TPP)	Enhancement	Shen et al. (2020)
Epigallocatechin gallate (EGCG)	Perilla	Enhancement	Shang et al. (2025)
*Rosmarinus officinalis*	*Tert*‐butylhydroquinone (TBHQ), citric acid (CA)	Enhancement	P. Ma, Liu, et al. (2025)
*Apium graveolens, Coriandrum sativum*	*Thymus vulgaris*	Enhancement	Bello et al. (2025)
α‐Tocopherol	Myricetin	Enhancement	Parra‐Escudero et al. (2025)
α‐Tocopherol	Gallic acid (GA)	Enhancement	Bayram and Decker (2023)
β‐Carotene (19:1 ratio)	Epicatechin	Enhancement	X. Wang et al. (2025)
Lycopene (1:15 ratio)	Gallic acid (GA)	Enhancement	X. Wang et al. (2025)
*Dillenia excelsa* (isolated compounds)	*Curcuma zedoaria* (isolated compounds)	Mostly enhancement effects	Sauli et al. (2025)
Ellagic acid (EA)	Lignin (lignosulphonate)	No interaction	Evtyugin and Evtuguin (2025)
*Dillenia excelsa* (crude)	*Curcuma zedoaria* (crude)	Antagonistic effect	Sauli et al. (2025)
α‐Tocopherol	Tea polyphenols (TPs)	Antagonistic effect	Bayram and Decker (2023)
β‐Carotene (1:19 ratio)	Epicatechin	Antagonistic effect	X. Wang et al. (2025)
Lycopene (1:9 ratio)	Gallic acid (GA)	Antagonistic effect	X. Wang et al. (2025)

### 7.1 Mechanistic Basis of Antioxidant Synergism

Mechanistically, synergism arises when chain‐breaking pathways (HAT, SET, PCET/SPLET) operate cooperatively with preventive actions (TMC, hydroperoxide decomposition) or when regeneration networks recycle oxidized antioxidants (Zhong et al. 2025). A well‐documented example is the regeneration of α‐tocopherol by PPs such as myricetin, which prolongs protection in both bulk oils and emulsions (Bayram and Decker 2024; Parra‐Escudero et al. 2025). Tea PPs can likewise stabilize and regenerate vitamin E and β‐carotene (Shen et al. 2020; Zhong et al. 2025).

In heterogeneous food systems, however, these mechanisms are strongly modulated by interfacial kinetics and diffusion constraints. Antioxidants must be co‐localized with the site of radical generation to participate effectively in chain‐breaking reactions. If compounds are segregated into different phases (e.g., aqueous vs. lipid), regeneration cycles and cooperative interactions may be kinetically hindered, even when thermodynamically favorable. Thus, synergy depends on both mechanistic compatibility and spatial proximity, requiring that antioxidants are co‐localized at sites of radical generation and able to interact within the same microenvironment.

Carotenoids (e.g., β‐carotene, lycopene) engage in sacrificial or energy‐transfer partnerships with vitamins E/C or tocotrienols, improving oxidative stability (Pan et al. 2025). Beyond small molecules, bioactive peptides and PP–protein interactions can suppress oxidation in protein‐rich foods, provided binding does not excessively reduce the freely active fraction (H. Chang et al. 2025; Rocchetti et al. 2026).

Apparent synergistic effects are method‐dependent. Solution‐phase assays (DPPH, ABTS, FRAP) overestimate cooperative effects by neglecting phase behavior and reaction kinetics; in situ lipid‐oxidation models capture spatial and temporal constraints; neither predicts in vivo synergy, because the systemic species are microbiota‐derived catabolites whose production depends on host metabotype (Iglesias‐Aguirre et al. 2023; Pidgeon et al. 2025). Formulation‐level synergy in food matrices is therefore necessary but not sufficient for human bioefficacy.

### 7.2 Role of Structure, Ratio, and Concentration

Structure and dose determine the sign and magnitude of interactions. Small shifts in the ratio of two antioxidants can flip outcomes from antagonism to synergy, reported, for example, for β‐carotene‐epicatechin and lycopene‐gallic acid in radical‐scavenging assays, highlighting the need for ratio optimization rather than fixed dosing (X. Wang et al. 2025).

These effects arise because antioxidant interactions follow nonlinear kinetics, in which radical flux, regeneration rates, and depletion dynamics are concentration‐dependent. At suboptimal ratios, one antioxidant may act as a pro‐oxidant or quench the activity of another, whereas balanced systems enable continuous radical interception and recycling.

At high concentrations or elevated oxygen partial pressure, several antioxidants (e.g., α‐tocopherol, PP‐rich extracts, β‐carotene) can become pro‐oxidant via radical propagation, Fe/Cu redox cycling, or aggregation‐driven sequestration away from interfaces (Jacobsen 2010; Sekhon‐Loodu et al. 2013). Some naturally coexisting constituents (e.g., lignin with ellagic acid) may show no interaction, underlining the specificity of multi‐compound effects (Evtyugin and Evtuguin 2025).

### 7.3 Matrix‐Driven Formulation Strategies

Effective antioxidant formulations require alignment between compound polarity, matrix structure, and oxidation locus. In emulsified systems, optimal performance is often achieved when antioxidants accumulate at the lipid–water interface, where radical initiation is concentrated. In bulk oils, lipophilic antioxidants dominate, whereas in aqueous or protein‐rich systems, hydrophilic compounds or protein‐bound phenolics may be more effective.

Synergy has also been demonstrated in co‐formulations that enhance stability and delivery, including co‐amorphous curcumin–piperine systems (Tian et al. 2025), β‐carotene embedded in graphene‐oxide hydrogels (Darban et al. 2024), and active‐packaging matrices combining EGCG with perilla extracts (Shang et al. 2025) or alginate coating with CaCl_2_ (Vargas‐Ramella, da Silva, et al. 2025).

However, these systems should be interpreted as case‐specific implementations of broader design principles, rather than universally transferable solutions. Their success depends on controlling release kinetics, protecting antioxidants from degradation, and ensuring their availability at the oxidation front over time.

### 7.4 Integrative Perspective on Synergistic Antioxidant Design

Taken together, synergistic antioxidant systems in food matrices emerge from the coordinated interplay between chemical reactivity, molecular interactions, and spatial distribution within the matrix. Effective combinations are those in which antioxidants are strategically positioned at sites of oxidation, operate through complementary mechanisms, such as radical scavenging, metal chelation, and regeneration, and maintain sufficient mobility and availability to sustain their activity over time. Conversely, mismatches between antioxidant polarity, localization, and matrix structure can lead to antagonism or apparent inactivity, even when individual components exhibit high intrinsic reactivity. Rational formulation, therefore, requires aligning physicochemical properties, interfacial behavior, and concentration windows with the target matrix, with validation under realistic storage conditions; the residual gaps in this approach, translational, methodological, and matrix–technology alignment, are addressed in Section 8.

## 8 Challenges and Future Directions

Progress in using plant‐based antioxidants is constrained by four interconnected gaps that span the chemical, food‐matrix, and human‐relevance domains introduced in Section 1. These gaps are not independent; each is conditioned on and aggravates the others. First, evidence linking intake to long‐term human outcomes remains limited. Although in vitro and short‐term in vivo findings are promising, translational relevance requires well‐designed human interventions that integrate bioaccessibility, bioavailability, and mechanism‐linked biomarkers. Importantly, digestion‐induced transformations, such as micellarization, enzymatic hydrolysis, and interactions with food macromolecules, can significantly alter antioxidant structure and functionality, further complicating the extrapolation of in vitro results to in vivo conditions. Daily‐intake data and validated exposure biomarkers are not equally mature across antioxidant classes. The EPIC biomarker study reported flavan‐3‐ol intakes spanning roughly threefold across European adult cohorts, with urinary (+)‐catechin and (−)‐epicatechin tracking acute rather than habitual intake; complementarily, urinary phenyl‐γ‐valerolactones show dose‐dependent associations with flavan‐3‐ol exposure, with two principal phenyl‐γ‐valerolactones (PVLs) accounting for >75% of measured urinary PVLs (Almanza‐Aguilera et al. 2021; Parmenter et al. 2023). Future work should therefore move from nominal‐composition and food‐frequency estimates toward biomarker‐anchored exposure frameworks when discussing the biological relevance of plant antioxidants.

Microbiota‐dependent biotransformation adds a further layer: The characterization of the *Enterocloster* uroC‐dehydroxylase (ucd) operon now provides a molecular mechanism for the three urolithin metabotypes (UM‐A, UM‐B, UM‐0) that have been reported in cohort studies, and ex vivo data demonstrate that only microbiota actively transcribing ucd can produce urolithin A from ellagitannin/ellagic‐acid substrates (Pidgeon et al. 2025). In observational cohorts, urolithin metabotype A (UM‐A; producing only urolithin A) is the most prevalent phenotype, whereas UM‐B (additionally producing isourolithin A and urolithin B) and UM‐0 (urolithin non‐producers) account for the remainder, with UM‐0 frequencies varying widely across populations, reported between approximately 7% and 25% in Spanish cohorts and substantially higher in some non‐European populations, and shifts in metabotype frequency have been documented with age and metabolic status (García‐Villalba et al. 2022; Iglesias‐Aguirre et al. 2023). Because pomegranate ellagitannins improved cardiovascular risk biomarkers (LDL‐c, apoB, oxidized LDL) selectively in UM‐B participants in controlled trials, population‐level dose recommendations cannot adequately describe the expected response to ellagitannin‐rich foods, and dose–response studies in antioxidant nutrition should be stratified by metabotype where feasible.

A further translational layer is the modulation of PP bioavailability by co‐ingested food components. Controlled human and in vitro digestion studies have shown that dairy proteins, dietary fibers, and lipid emulsifiers can alter the kinetic profile of flavan‐3‐ol absorption and microbial conversion, with whole‐food matrices sometimes preserving rather than restricting bioaccessibility relative to the corresponding extracts (Cattivelli et al. 2025). The mechanistic basis lies in deglycosylation, hepatic conjugation, and microbial ring‐cleavage steps that are themselves substrate‐specific (Pidgeon et al. 2025). Consequently, the dietary‐relevance evaluation of any antioxidant‐enriched food must consider both the parent‐compound dose and the co‐formulated proteins, lipids, and fibers that govern kinetic delivery and microbial conversion.

Second, methods are not standardized: Variations in extraction, assay choice, and test conditions limit comparability and obscure structure–activity signals across studies. Moreover, many commonly used antioxidant assays fail to capture key aspects governing antioxidant performance in real foods, such as phase distribution, interfacial localization, and reaction kinetics, which contributes to discrepancies between measured activity and actual efficacy in food matrices. Harmonized protocols are needed to guarantee efficacy and guide formulation.

Third, technology–matrix alignment is underexplored. For encapsulated systems, stability, release, and bioaccessibility are determined by interactions with proteins, lipids, fibers, and processing stresses; hence, evaluations in real food matrices should be prioritized over those in model systems. In particular, the effectiveness of delivery systems depends on their ability to control spatial distribution and release kinetics in relation to oxidation‐prone regions, such as lipid–water interfaces or metal‐rich domains, rather than solely on their encapsulation efficiency.

Lastly, consumer acceptance also remains a gap—encapsulation can attenuate bitterness/astringency, but robust, consumer‐based sensory testing is sparse; sensory should be integrated with nutritional, technological, and stability endpoints. The integration of sensory perception with physicochemical and functional data is essential to ensure that technological improvements translate into consumer‐relevant benefits. Methodological advances, such as semi‐dynamic/dynamic digestion models and in silico predictive tools, may help reconcile discrepancies between in vitro and in vivo outcomes by better reflecting physiological conditions.

Three limitations of the present synthesis should be acknowledged. First, the evidence base is heterogeneous: Outputs from solution‐phase chemical assays (DPPH, ABTS, FRAP, ORAC) cannot, by themselves, support claims of physiological efficacy and are used here only as descriptors to be cross‐checked against interfacial kinetics, in‐matrix lipid‐oxidation markers, and biomarker‐validated estimates of human exposure. Second, the depth of mechanistic and kinetic coverage is uneven across antioxidant classes: Flavan‐3‐ols, anthocyanins, and ellagitannins are well supported by intervention trials and metabotype data, whereas equivalent translational evidence for tocotrienols, lignans, and most alkaloids is still scarce, and class‐level conclusions reflect that imbalance. Third, the regulatory dimension (labeling, health claims, efficacy, and bioavailability requirements), although critical for lab‐to‐market translation, is not analyzed here, as it lies outside the chemical, matrix, and physiological scope of the review. The gaps identified above define a research agenda in which chemical reactivity, spatial distribution within the food matrix, and biological relevance need to be addressed in parallel.

## 9 Conclusions

Plant‐based antioxidants perform best in food and physiological systems when their chemistry is matched to the target matrix. Effective protection depends on interfacial localization at oil–water boundaries (where lipid peroxidation initiates), on competitive interactions with biopolymers, and on the control of metal‐catalyzed initiation; the antioxidant dose, although necessary, is not sufficient on its own. Formulation decisions should accordingly address the amount of antioxidant added, its location in the matrix, the partner ingredients with which it interacts, and the time scale over which it must remain functional. In practice, this is achieved by combining matrix mapping with kinetics‐guided degradation models to set processing and packaging windows, and by selecting delivery systems that position and protect the active compound through manufacture, storage, and digestion.

Multi‐antioxidant systems can deliver superior protection when scavenger–chelator or regenerator ratios are tuned, but the same blends may turn antagonistic or pro‐oxidant under high O_2_ or suboptimal dosing, reflecting the interplay between reaction mechanisms, spatial localization, and oxygen availability.

From a practical standpoint, integrating reaction mechanisms, matrix interactions, and degradation kinetics provides a predictive basis for selecting antioxidant systems and defining processing, storage, and packaging conditions in real food applications. Ultimately, in situ validation with oxidation models and physiologically relevant digestion assays is essential to verify that these design rules translate into real shelf life and quality gains in the intended food.

Earlier reviews have addressed plant‐antioxidant chemistry and food processing largely in isolation. The synthesis presented here connects chemical reactivity, food‐matrix interactions, degradation kinetics, and human‐relevance considerations under a common analytical scheme. To support claims about efficacy in humans, this scheme must be combined with biomarker‐validated exposure data, with studies of food‐component interactions during digestion, and with stratification by gut–microbiota metabotype so that formulation decisions are evaluated against the metabolite pool that actually reaches systemic circulation. Validation of matrix‐engineered antioxidant performance in dietary‐exposure cohorts and, where the evidence base permits, in metabotype‐stratified human trials, remains a central research priority.

## Author Contributions

**Márcio Vargas‐Ramella**: conceptualization, data curation, validation, formal analysis, supervision, visualization, funding acquisition, writing – review and editing, writing – original draft. **Carmen Silvia Favaro‐Trindade**: conceptualization, supervision, formal analysis, validation. **Bárbara Miranda‐Vilela**: validation, investigation, writing – original draft. **Mariana Estefanuto‐Costa**: investigation, validation, writing – original draft. **Marina Franco‐Brito**: investigation, validation, writing – original draft. **Bibiana Alves dos Santos**: validation, formal analysis, writing – review and editing. **Paulo Cezar Bastianello Campagnol**: validation, formal analysis, supervision, writing – review and editing, visualization, methodology.

## Funding

This work was supported by Universidade do Estado de Santa Catarina (UDESC), and the Article Processing Charge (APC) for the publication of this research was funded by the Coordenação de Aperfeiçoamento de Pessoal de Nível Superior—Brasil (CAPES).

## Conflicts of Interest

The authors declare no conflicts of interest.

## Data Availability

Data are provided within the manuscript and Supporting Information files. Additional data will be made available on request.
